# Evaluating the performance of spatial indicators of destination accessibility for physical activity research: a comparative international analysis

**DOI:** 10.1016/j.cities.2025.106044

**Published:** 2025-05-03

**Authors:** Ester Cerin, Marc A. Adams, Terry L. Conway, Lawrence D. Frank, Kelli L. Cain, Poh-chin Lai, Scott Duncan, Ana Queralt, Anna Timperio, Jasper Schipperijn, Rodrigo S. Reis, Delfien Van Dyck, Paulina P.Y. Wong, Jan Dygrýn, Erica Hinckson, Javier Molina-Garcia, James F. Sallis

**Affiliations:** a Mary MacKillop Institute for Health Research, Australian Catholic University, Melbourne, Victoria, Australia; b School of Public Health, The University of Hong Kong, Hong Kong, China; c College of Health Solutions, Arizona State University, Phoenix, AZ, USA; d Herbert Wertheim School of Public Health & Human Longevity Science, University of California, San Diego, CA, USA; e Department of Urban Studies and Planning, University of California, San Diego, CA, USA; f Urban Design 4 Health, Inc. Rochester, New York, USA; g Department of Geography, The University of Hong Kong, Hong Kong, China; h School of Sport and Recreation, Auckland University of Technology, Auckland, New Zealand; i AFPIS Research Group, Department of Nursing, University of Valencia, Valencia, Spain; j Epidemiology and Environmental Health Joint Research Unit, FISABIO-UJI-UV, Valencia, Spain; k Institute for Physical Activity and Nutrition, Deakin University, Geelong, Victoria, Australia; l World Playground Research Institute, University of Southern Denmark, Odense, Denmark; m People Health and Place Unit, Washington University in Saint Louis, St. Louis, MO, USA; n Department of Movement and Sports Sciences, Ghent University, Ghent, Belgium; o Science Unit, Lingnan University, Tuen Mun, Hong Kong, China; p Institute of Active Lifestyle, Faculty of Physical Culture, Palacký University Olomouc, Olomouc, Czech Republic; q AFIPS Research Group, Department of Teaching of Physical Education, Arts and Music, University of Valencia, Valencia, Spain

**Keywords:** Destination density, Land use mix, Active transport, Device-assessed physical activity, Multi-site international study

## Abstract

Neighbourhoods with good access to various destinations facilitate engagement in physical activity. Because spatial indicators of destination accessibility can be operationalised in numerous ways, it is important to evaluate their ability to explain physical activity in various geographical and cultural contexts. We evaluated established as well as novel spatial indicators of destination accessibility using data from 12 cities/regions participating in an international study on adolescents’ physical activity. Twelve spatial indicators of destination accessibility were developed using land use data. Five indicators represented measures of destination intensity (density), two were indicators of destination heterogeneity [land use mix (LUM) indices], and the remaining five were novel measures of combined destination intensity and heterogeneity. To evaluate these spatial indicators, we examined their performance as correlates of parent-perceived destination accessibility and adolescents’ physical activity, and the extent to which the findings were generalisable across cities/regions. Substantial differences in ability to explain physical activity and comparability across geographical locations were observed across the indicators. The best performing indicator, defined as being a consistent correlate of parental perceptions of destination accessibility and adolescents’ physical activity in the expected direction and across cities/regions, was a novel hybrid intensity + heterogeneity indicator: gross density of non-residential destinations weighted by a novel parcel-count-based LUM index. Indicators based on ratios of non-residential land/parcels to residential land/dwelling units performed poorly on most criteria, while LUM indices performed well in relation to transport-related physical activity. We provide recommendations regarding the usage of spatial destination accessibility indicators in physical activity research.

## 1. Introduction

Features of the environment in which people perform their daily activities, such as residential neighbourhoods, can influence physical activity patterns (Barnett et al., 2017; McCormack & Shiell, 2011; Nordbø et al., 2020; Omura et al., 2020; Saelens et al., 2003; Transportation Research Board, 2005) and, consequently, health (World Health Organization, 2009). Starting from the 1990s, after urban sprawl was recognised as a public health concern, a plethora of studies have investigated the role of the neighbourhood built environment in shaping travel behaviours, including walking and cycling and, later, other forms of physical activity (Ewing, 2005; Frank & Pivo, 1994). The focus of this research was on three characteristics underpinning the construct of neighbourhood walkability – namely, density (e.g., population or dwelling density), diversity (land-use mix) and design (e.g., street connectivity) (Cervero & Kockelman, 1997). Overall, the accumulation of 30 years of empirical evidence suggests that walkable residential neighbourhoods are associated with more active transport, overall physical activity (Omura et al., 2020; Zhang et al., 2024) and lower odds of overweight/obesity in all age groups (Chandrabose et al., 2019; Malacarne et al., 2022).

Among the components of neighbourhood walkability, the availability of relevant destinations within walking distance from home (here termed “destination accessibility”) is perhaps the most important in relation to physical activity because it facilitates active transport, recreational walking, and, depending on the type of available destinations, other active pursuits (e.g., sports and fitness activities) (Giles-Corti et al., 2009; Saelens et al., 2003; Transportation Research Board, 2005). However, destination accessibility is also the most challenging aspect of neighbourhood walkability to operationalise, as there are many more possible ways of defining destination accessibility than density or street connectivity. Thus, understanding the utility and generalisability of different operationalisations of destination accessibility across different physical activity behaviours, populations and contexts is of paramount importance.

Destination accessibility (e.g., distance, diversity, and availability of destinations) has been measured objectively using Geographic Information Systems (GIS), which leverage existing data sources (Adams et al., 2014; Frank et al., 2017), as well as subjectively via questionnaires (self-reports) (Cerin et al., 2013; Rosenberg et al., 2009). Compared to self-reports, GIS-based measures of destination accessibility have the advantage of providing an objective assessment of residential neighbourhoods independent of individual experiences and biases, making them more appropriate for the estimation of potential causal effects of the environment on physical activity and health (Barnett et al., 2018). GIS-based measures also provide geographically-specific data and metrics that are relevant to city planning decision-making. Hence, this paper focuses on the development and validation of GIS-based measures of destination accessibility.

We first present an overview of the spatial indicators of destination accessibility used in physical activity research, identify challenges posed by international multi-site studies, and use this information to formulate, and provide a rationale for, the study objectives. The Methods section describes the study design and sites (cities/regions), data collection methods, operationalisation of GIS-based measures of destination accessibility, and data analytic steps. The organization of the Results section mirrors the study objectives. Finally, we discuss the study findings by type of spatial indicators of destination accessibility, reflect on and consider the practical implications of the observed differences across cities, and make final recommendations regarding the utility and appropriates of the various spatial indicators for international physical activity research.

## 2. Spatial indicators of destination accessibility in physical activity research: literature review

In the physical activity and health literature, GIS-based measures of destination accessibility have been operationalised in numerous ways. These include the following categories of spatial indicators: (1) percentage or total land area devoted to a specific use (e.g., commercial or institutional) within one’s neighbourhood, typically defined as a sum of specific land areas within a certain walkable distance from home (e.g., 0.5 or 1 km buffer) (Cerin et al., 2021; Frank et al., 2017); (2) count or density of points for specific types of destinations (e.g., parks, shops, public transit) within one’s neighbourhood (Cerin, Barnett, et al., 2020; Frank et al., 2017); (3) areal or street-network distance from home to the nearest destination of a specific type (Sallis et al., 2016; Timperio et al., 2006); (4) total count or density of different types of destinations within one’s neighbourhood (Chaix et al., 2014; Spoelder et al., 2023); and (5) mix of land uses or destinations within one’s neighbourhood (Adams et al., 2014; Frank et al., 2017), which represent measures of heterogeneity rather than density or ‘intensity’ of destinations. The first validated destination-based land use mix index was created in the early 1990s and captured the evenness of distribution (or entropy) of floor space across several different land uses using parcel data in Seattle (Frank & Pivo, 1994).

Using multiple indicators from categories 1 to 3 mentioned above (e. g., point density of shops and public transit stops; percentage of commercial land and percentage of entertainment land) in a single regression model may create computational problems due to multicollinearity (Cervero & Radisch, 1996; Saelens et al., 2003), an issue that led to the establishment of a widely used composite walkability index (Frank et al., 2010) that integrates land use mix with connectivity and density. Composite indicators of destination accessibility based solely on destination / land use data (here represented by spatial indicators under categories 4 and 5 mentioned above) also circumvent multicollinearity problems. However, they remain underexplored and are, thus, the focus of this paper.

### 2.1. Composite indicators of destination accessibility

Total count or density of different types of destinations within a neighbourhood is primarily a composite *intensity* measure of destination accessibility, although it could be argued it may capture destination heterogeneity because the presence of multiple types of destinations in a neighbourhood may result in higher overall density of destinations. In support of the utility of these measures, studies have reported positive associations between total count or density of destinations and physical activity in youth (Prince et al., 2022a), adults (Chaix et al., 2014; Prince et al., 2022b) and older adults (Barnett et al., 2017; Cerin et al., 2017). As neighbourhoods may have a relatively low density but high variety of destinations (e.g., a single shop, recreational facility, park and school) or a high density of a single type of destinations (e.g., 50 shops but no other types of non-residential destinations), in assessing destination accessibility, it is also important to more accurately capture land use or destination heterogeneity.

*Heterogeneity* of land uses or destinations, often called land use mix (LUM), can be defined in different ways (Song et al., 2013). In the physical activity literature, it is usually operationalised as an entropy index (Christian et al., 2011; Frank et al., 2006, 2010; Song et al., 2013) based on Shannon’s algorithm (Shannon, 1948), which was introduced in the early 90s by Frank and Pivo using the distribution of floor space across residential and non-residential uses within a census tract to explain active transport (Frank & Pivo, 1994). Many studies (Christian et al., 2011; Frank et al., 2006, 2010; Song et al., 2013) have since expanded on this concept. The LUM entropy index takes into account the relative proportion of two or more land uses within an area. It ranges from 0 to 1, where 0 indicates a single land use and 1 denotes an even distribution of different land uses within an area (e.g., 20 % of each of five land uses). The concept of “evenness” has also been challenged. One study found it was more important to have a range of walkable destinations in close proximity than having an even distribution of destination types (Brown et al., 2009). Living in a neighbourhood with diverse land uses legally enables a variety of nearby destinations to emerge for daily living and active pursuits, encouraging more walking or cycling. Empirical evidence has, in general, provided support for this hypothesis, with many studies reporting positive associations between LUM indices and physical activity, especially transport-related physical activity, in adults (Prince et al., 2022b) and older adults (Cerin et al., 2017), but not as much in youth (Prince et al., 2022a), possibly due to the general dearth of findings and these indicators being less relevant to physical activity among youth.

Apart from the two common sets of indicators mentioned above, other operationalisations of destination accessibility could prove useful to physical activity research. For example, the ratios of non-residential land or destinations to residential land or dwellings in a neighbourhood represent simple measures of LUM that are linked to the concept of job-housing balance, which grew out of commute related congestion and was the primary land use focused transport policy directive for over a 20-year period (Levine, 1998). The goal of jobs-housing balance was to reduce commute distance and overall air pollution by shortening commute distances. Spatial indicators of job-housing balance represented by, for example, the ratio of non-residential to residential land use provide information on exposure and supply of non-residential destinations to residents and, hence, may be also related to physical activity.

Total destination density has typically been operationalised as total gross density (i.e., relative to the total area of a neighbourhood) rather than total net density (i.e., relative to the area for specific non-residential land uses) (McConville et al., 2011). There are instances where net destination density is more relevant to physical activity. For example, single-storey non-residential destinations scattered around the neighbourhood may be less effective in promoting active transport than destinations conveniently clustered in multistorey buildings that residents can visit for multiple purposes. While net destination density indicators can differentiate between these two scenarios, their gross counterparts cannot.

LUM entropy indices have been operationalised using information on actual floor space per land use type (Frank et al., 2006, 2010; Frank & Pivo, 1994) which captures the 3-dimensional nature of urban development. For example, buildings often have multiple stories captured in the commercial floor space ratio metric. However, floor space data are not usually available everywhere. Multi-country studies and investigations spanning whole nations requiring consistent metrics (Adams et al., 2014; Christian et al., 2011; Song et al., 2013) use land area metrics. There is an important distinction between numbers of activities or businesses versus land area. For example, a 9000 m^2^ commercial area within a neighbourhood could be occupied by a single large parcel hosting a warehouse or, alternatively, a shopping mall with dozens of smaller but diverse parcels and services (e.g., grocery shop, bank, post office, etc.). For studies lacking floor space data, we posit that quantifying the mix of destinations may be better captured through the evenness of distribution of the number of parcels rather than total land area of parcels across use types. We posit that parcel-count-based LUM entropy indices would be more strongly related to physical activity than land area-based counterparts. This is because the number of parcels per use better characterises the diversity of distinct destinations in the neighbourhood. Finally, if we are to use a single GIS-based composite measure of destination accessibility to explain physical activity behaviours, it might be useful to combine indicators of intensity and heterogeneity of land use/destinations into one ‘hybrid’ measure. For example, intensity indicators could be weighted by indicators of heterogeneity. Yet, to our knowledge, no study has examined the explanatory power of such hybrid or combined indicators. We hypothesise hybrid measures may be more strongly positively related to physical activity, especially utilitarian walking, than their simpler counterparts capturing solely intensity or heterogeneity of land uses/destinations.

### 2.2. Destination accessibility in international multi-site studies

Because the variability of urban environmental features within a single location (e.g., a city) can be limited (Adams et al., 2014) and the effects of the environment on physical activity may vary across cultural and geographical contexts (Cerin et al., 2022; Cerin et al., 2020), international multi-site studies of environment and physical activity associations are necessary for understanding similarities and differences across cities and countries (Kerr et al., 2016; Sallis et al., 2016). To illustrate, only through international studies employing comparable measures of the built environment and physical activity, while including a diversity of locations across the whole density spectrum, can we determine the shape of relations between residential density and physical activity, along with optimal thresholds of density (if any). This is because such relations may not be linear, and single cities/regions often cover only a part of the density spectrum (Cerin et al., 2022). Additionally, it is only through such research that we can understand whether the effect of a specific urban feature (e.g., destination accessibility) on physical activity depends on contextual factors, such as climatic conditions or culture, and, hence, requires approaches to urban planning tailored to the local context.

Although desirable, international multi-site studies pose methodological and interpretational challenges. The availability and resolution of spatial data differ across countries and even across regions within the same country. This makes it difficult to develop comparable spatial indicators for pooled analyses. Capturing consistent variation in built environment features across multiple regions was the innovation and focus of the (NQLS) Neighbourhood Quality of Life Study (Frank et al., 2010). NQLS integrated parcel and other land use and transportation data from Seattle and Baltimore into a single walkability index. NQLS was built off previous research (Frank et al., 2006; Frank & Pivo, 1994) and then broadly expanded into the 12-country study of adults, the International Physical Activity and the Environment Network (IPEN) Adult study (Adams et al., 2014). The IPEN Adult study found different cities and countries used widely varying coding schemes and land use categories that made the creation of comparable area-based LUM indices particularly challenging (Adams et al., 2014). The authors concluded that substantial efforts and care were needed to develop comparable spatial indicators for multi-site studies. This body of spatial methodological advancements in GIS evolved over 3 decades and has effectively demonstrated how valid and reliable comparative international work can be achieved.

The between-site comparability of a spatial indicator should be based on the utilisation of comparable data and methods, and also on the indicator having a comparable meaning and interpretation across study sites (e.g., showing similar associations with perceived destination accessibility across study sites). The interpretation of spatial indicators of destination accessibility may sometimes depend on the density and overall urban form of the study location. For example, the ratio of non-residential parcels to dwelling units may not be a particularly useful indicator of LUM for ultra-dense cities (i.e., with extreme levels of population density) where diverse types of destinations are ubiquitous. This is because, in such circumstances, lower ratios of non-residential parcels to dwelling units do not represent poor availability of destinations but, rather, extreme levels of population density. Hence, in developing or recommending spatial indicators of destination accessibility for international research and city-planning policy, it is important to consider both methodological and construct validity comparability.

## 3. This study

The overarching objective of the present study was to develop and evaluate the construct validity of a set of established as well as novel GIS-based indicators of destination accessibility. To enhance international relevance, the study was conducted in diverse geographical and cultural contexts using data from 12 cities/regions from five continents participating in an international study on adolescents’ physical activity (Cain et al., 2021). The ultimate goal was to identify spatial indicators of destination accessibility that are most strongly related to adolescents’ physical activity and their parents’ perceptions of destination accessibility across geographical contexts, permitting pooled analyses in international studies of the built environment and physical activity. We chose to focus on adolescents because they are an understudied age group in this field (Ding et al., 2011).

To achieve the study objective, twelve spatial indicators of destination accessibility were developed using land use data that was commonly available across countries and cities. We used land use rather than more granular destination data (e.g., sport facilities) because the former are directly relevant to urban planning and policy (zoning), more readily available and easier to classify across study sites, more temporally stable, included in standard measures of neighbourhood walkability and pertinent to all age groups. Five of the spatial indicators represent measures of destination intensity (density), two are indicators of destination heterogeneity [land use mix (LUM) indices], and the remaining five are novel measures that combined destination intensity and heterogeneity.

**Fig. 1.**
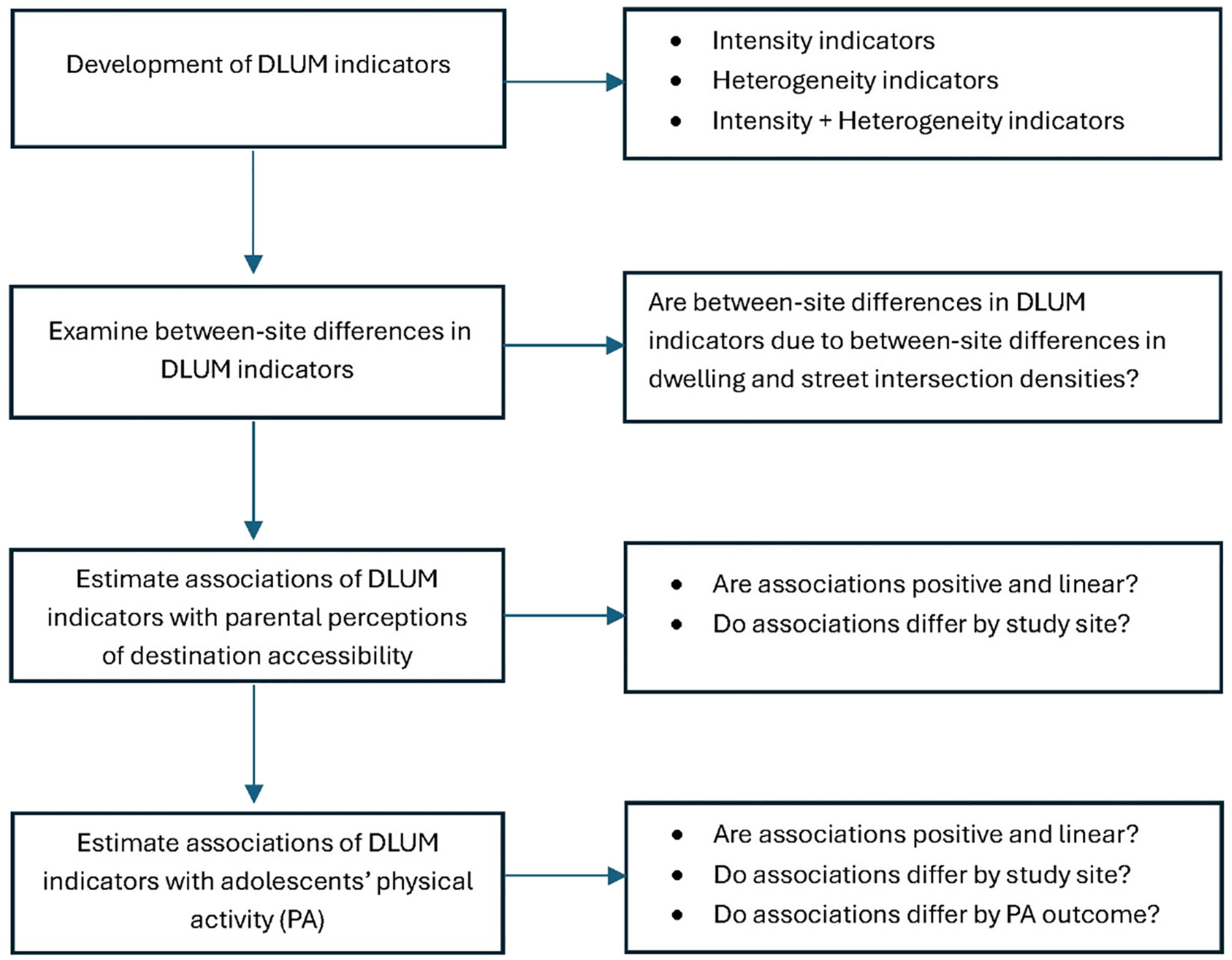
Process of evaluation of spatial indicators of destination accessibility [destination & land use mix (DLUM) indicators].

We evaluate spatial indicators in several steps (Fig. 1), starting from an examination of their within- and between-site distributions, and between-site differences, to understand the extent to which they capture similar aspects of destination accessibility in cities of differing density and connectivity. We then estimate the associations of each spatial indicator with adult residents’ (in this case, adolescents’ parents) perceptions of neighbourhood destination accessibility and the extent to which these associations vary by site. This information is crucial for assessing the utility of low-granularity land-use-based spatial indicators for measuring access to actual destinations (e.g., shops, recreation facilities) supporting activities of daily living and physical activity across various geographical contexts. Lastly, we examine the performance of the 12 spatial indicators of neighbourhood destination accessibility as correlates of adolescents’ physical activity, and the extent to which the findings are generalisable across sites. In doing so, we consider total physical activity, non-school physical activity, active transport to/from school and active transport to other types of destinations. These multiple outcomes allow us to examine whether the performance of spatial indicators of neighbourhood destination accessibility depends on the type and context of physical activity behaviours.

## 4. Methods

We utilised comparable GIS, survey and device-based physical activity data collected in 12 cities/regions across nine countries that participated in the IPEN Adolescent study. The selected study sites varied substantially in net dwelling density, motor vehicles per capita, prevalence of cycling to/from school, and cultural regions (Fig. S1), enabling an evaluation of spatial indicators of destination accessibility in various geographical and cultural contexts. The following sections detail the study design of IPEN Adolescent and the measures used in the present study, including neighbourhood GIS-based spatial indicators of destination accessibility [here named destination and land-use mix (DLUM) indicators] and other attributes, parental perceptions of destination accessibility, and adolescents’ physical activity.

### 4.1. Study design and sample

IPEN Adolescent is an observational, cross-sectional study conducted in 15 countries across six continents (Cain et al., 2021). Nine of these countries, including 12 cities/regions and a total of 5052 participants, had access to comparable GIS data. These were Australia (AUS, Melbourne), Belgium (BEL, Ghent), Brazil (BRA, Curitiba), China (CHN, Hong Kong), Czech Republic (CZE, Hradec Kralove and Olomouc), Denmark (DNK, Odense), New Zealand (NZL, Auckland and Wellington), Spain (ESP, Valencia) and the United States of America (USA, Baltimore and Seattle regions) (Table 1; Table S1; Fig. S1; Supplementary Material).

A stratified two-stage sampling strategy was employed to recruit adolescents (aged 11–19 years) from study sites. All sites, except Auckland and Wellington (NZL), also recruited one of the adolescents’ primary caregivers who in a separate survey self-reported proximity of multiple types of destinations from home (*n* = 4415). The sampling strategy first entailed the selection of census/administrative unit areas stratified into quadrants of higher- vs. lower-walkability by higher- vs. lower-socioeconomic status (Fig. S1; see Supplementary Material for details). The IPEN Adolescent study was approved by the the Institutional Review Boards at San Diego State University and the University of California San Diego. Study protocols and data collection in each country were approved by their Institution’s Ethics Committees. Details on the Ethics Boards and approval numbers are provided in Cain et al. (2021). Written informed consent/assent was obtained from all participants. Detailed information on the study design, sites, and recruitment procedures was previously reported (Cain et al., 2021).

While all 9 countries had relatively complete self-report data on adolescents’ active travel to/from school and outside of school physical education classes, only a subsample of adolescents had valid accelerometer-based physical activity data (*n* = 3481) (Table 2). Brazil and Spain did not collect adolescents’ self-report data on active transport to non-school destinations (Table 2).

### 4.2. Measures

#### 4.2.1. GIS-based neighbourhood characteristics

GIS staff in each country followed a predeveloped common set of 15 GIS templates based on previous work (available at https://ipenproject.org/) that gave guidance on creating GIS variables and specific procedures. The data were iteratively reviewed by two experts. The overall approach ensured a common set of comparable GIS variables and allowed for the documentation of any deviations. (see Supplementary Material for details).

Participants’ residential addresses were geocoded and road network-based buffers of prespecified sizes were created in GIS around each participant’s home (Frank et al., 2017). The present analyses used only 1-km-street network sausage buffer, with 25 m offset, because they capture parcels adjacent to the road and have shown more consistent associations with physical activity (Frank et al., 2017). For each buffer, a wide range of GIS-based environmental attributes were computed. This study utilised the following subset: net residential density (dwellings/km^2^ of residential land); street intersection density (intersections/km^2^ of total buffer area); gross parcel densities of each of five groups of land uses (parcels/km^2^ of total buffer area), i.e., (1) commercial/retail/office, (2) institutional/civic, (3) food-related, (4) entertainment, and (5) parks; and 12 destination and land-use mix (DLUM) indicators. The DLUM indicators are shown in Table 3 and consisted of five indicators of overall availability/intensity of the five groups of non-residential land uses or destination types (DLUM 1 to DLUM 4), two indicators of heterogeneity (entropy) of land uses or destination types (DLUM 5 and 6), and five indicators representing hybrid measures of availability/intensity and heterogeneity of the five groups of non-residential land uses or destinations (DLUM 7 to DLUM 10).

#### 4.2.2. Parent-perceived proximity of destinations from home

For one evaluation of construct validity, we used items from the Neighbourhood Environment Walkability Scale for Youth (NEWS-Y) (Cerin et al., 2019) to assess parental perceived proximity of 15 destinations from home, representing perceived destination accessibility. Caregivers reported the walking time to reach the nearest destination (of a specific type) from home. Responses ranged from 1 to 5-min walking distance (assigned a score of 5, i.e., high accessibility) to 30-min or more walking distance (assigned a score of 1, i.e., low accessibility). Five composite measures were computed to mirror the self-report counterpart GIS-based measures. First, an overall score of the average responses across all 15 types of destinations, named “proximity of destinations from home,” represented the self-report counterpart measure of the GIS-based destination and land-use mix indicators. The other four measures represented average scores for perceived proximity of parks, commercial/office, institutional/civic, and food-related destinations. No NEWS-Y items captured entertainment destinations. Details are provided in the Supplementary Material.

#### 4.2.3. Adolescent’s physical activity

This study examined several self-report and accelerometer-based measures of adolescents’ physical activity, used as a second set of construct validity measures. Self-report measures included: total non-school physical activity [defined as days per week of meeting the physical activity guidelines of 60+ min/day (Cerin et al., 2014; Prochaska et al., 2001; Ridgers et al., 2012)]; regular active transport to/from school (5+ walking plus cycling trips per week); regular walking to/from school (5+ walking plus cycling trips per week), and non-school active transport (total frequency of walking or cycling to/from six destinations in a typical week). Adolescents were also asked how long it would take them to walk to school, as a proxy for distance to school. These items were adapted from the U.S. Centers for Disease Control and Prevention’s Kids-Walk-to-School program (National Center for Chronic Disease Prevention and Health Promotion (U.S.) (CDC), 2000) and showed good test-retest reliability in various populations (Cerin et al., 2014; Joe et al., 2023). All study sites collected data on all self-report measures of physical activity, except non-school active transport was not included in Curitiba (BRA) and Valencia (ESP). Accelerometer-based measures of physical activity were average daily minutes of moderate-to-vigorous physical activity (MVPA) and average daily minutes of MVPA during non-school periods as detailed in the Supplementary Material and elsewhere (Cain et al., 2021).

#### 4.2.4. Socio-demographic characteristics

Participants (adolescents and/or parents) self-reported socio-demographic characteristics, including adolescents’ sex and age, parental marital status, highest education in the household, household composition, and motor vehicles in the household.

### 4.3. Data analysis

Random intercept generalised additive mixed models (GAMMs) accounting for clustering at the administrative unit and school levels were used to: (aim 1) estimate between-site differences in DLUM indicators; (aim 2) establish the extent to which these differences were due to residential and intersection densities; (aims 3 and 4) estimate associations of DLUM indicators with parental perceived proximity of destinations from home and adolescents’ physical activity; and (aim 5) determine whether these associations were generalisable across study sites (Fig. 1). GAMMs can accommodate outcomes with different distributions and model curvilinear associations of unknown form using semiparametric smooth terms (in this case, thin plate splines) (Wood, 2017). Analyses were performed on 20 imputed datasets because >5 % of cases (12.8 % to 18.1 %) had missing data on at least one of the variables included in regression models (van Buuren, 2018). The analytical approach is detailed in the Supplementary Material.

## 5. Results

Parental perceptions of proximity of various destinations, except for parks, were the lowest in the US sites (Table 1). Valencia (ESP) had the highest average ratings across all perceived proximity measures, followed by Hong Kong (CHN) with respect to overall, commercial/office and food-related destinations. Hradec Kralove (CZE) had one of the highest average ratings of proximity of institutional/civic destinations, while Melbourne (AUS) had one of the highest average proximity of parks.

As to adolescents’ physical activity, between-site differences depended on the type of measure (Table 2). Seattle (USA) had the lowest, and Melbourne (AUS) and Hong Kong (CHN) the highest, average weekly frequencies of walking or cycling to/from destinations. In contrast, Curitiba (BRA) and Hong Kong (CHN) had the lowest average days per week with at least 60 min/day of non-school physical activity, while Odense (DNK) and Wellington (NZL) were the best performing sites on this physical activity measure. Active commuting to/from school was the lowest in the US and among the highest in Valencia (ESP). Although Odense (DNK) had one of the lowest frequencies of walking to/from school, it had the highest frequency of overall active commuting to/from school (due to high frequency of biking). Olomouc (CZE) and, to an extent, Wellington (NZL) had consistently higher, and Hong Kong (CHN) lower, average accelerometry-based MVPA (Table 2).

**Fig. 2.**
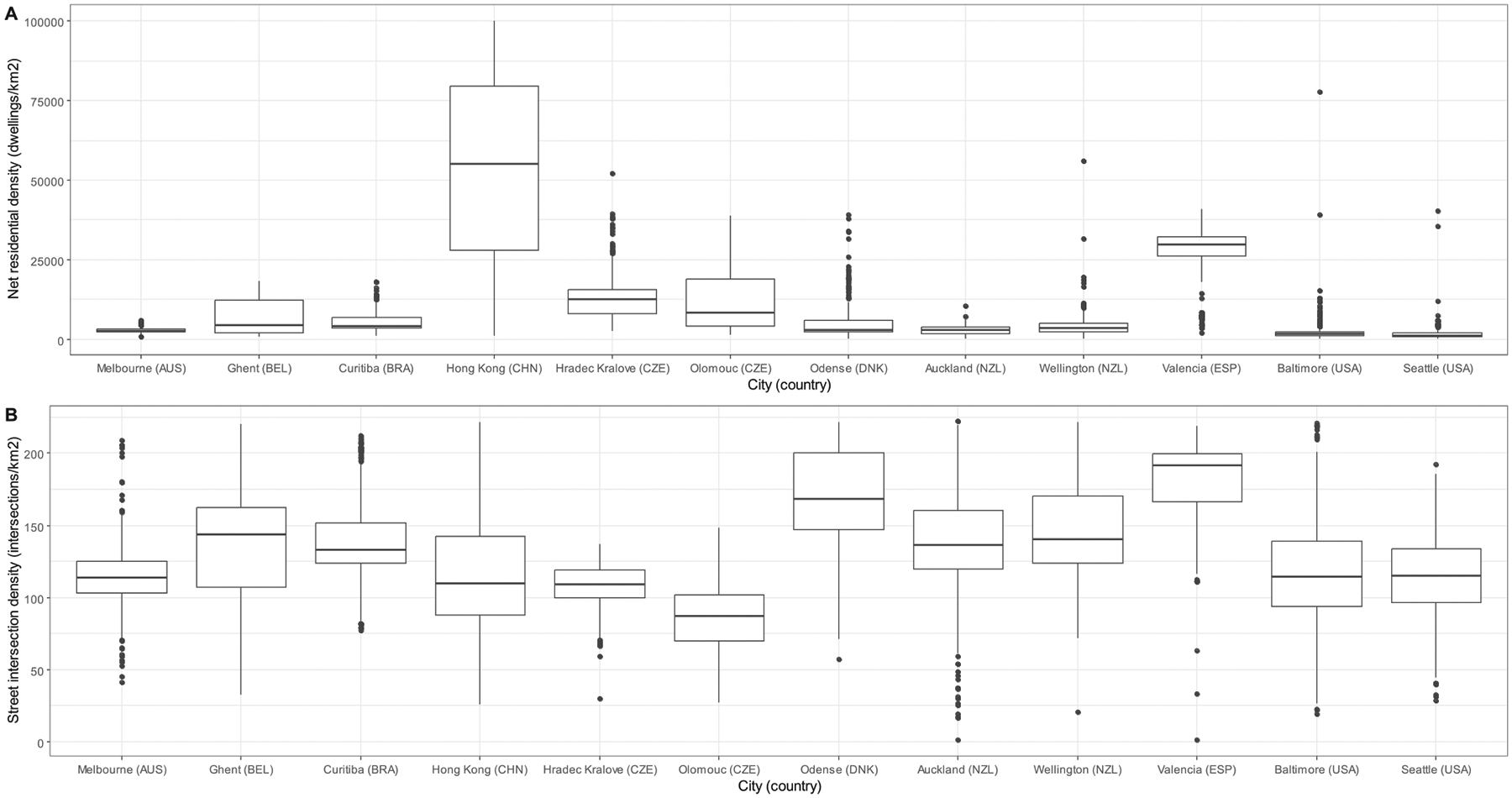
Distribution of net residential density (dwellings/km^2^) (panel A) and street intersection density (intersections/km^2^) (panel B) by study site.

Substantial between-site differences were observed in residential and street intersection densities (Fig. 2; Table S1, Supplementary Material). Melbourne (AUS) and the US sites had the lowest levels (Mdn = 1109.3 to 1622.3 dwellings/km^2^; IQR = 1231.6 to 1148.7 dwellings/km^2^) as well as spread of net residential density, while Hong Kong (CHN) had the highest (Mdn = 68,745.9 dwellings/km^2^; IQR = 57,631.3 dwellings/km^2^). Valencia (ESP) ranked first in street intersection density (Mdn = 194.7 intersections/km^2^; IQR = 32.1 intersections/km^2^) and second in net residential density (Mdn = 29,842.1 dwellings/km^2^; IQR = 6079.5 dwellings/km^2^).

### 5.1. Between-site differences in GIS-based destination and land-use mix indicators

**Fig. 3.**
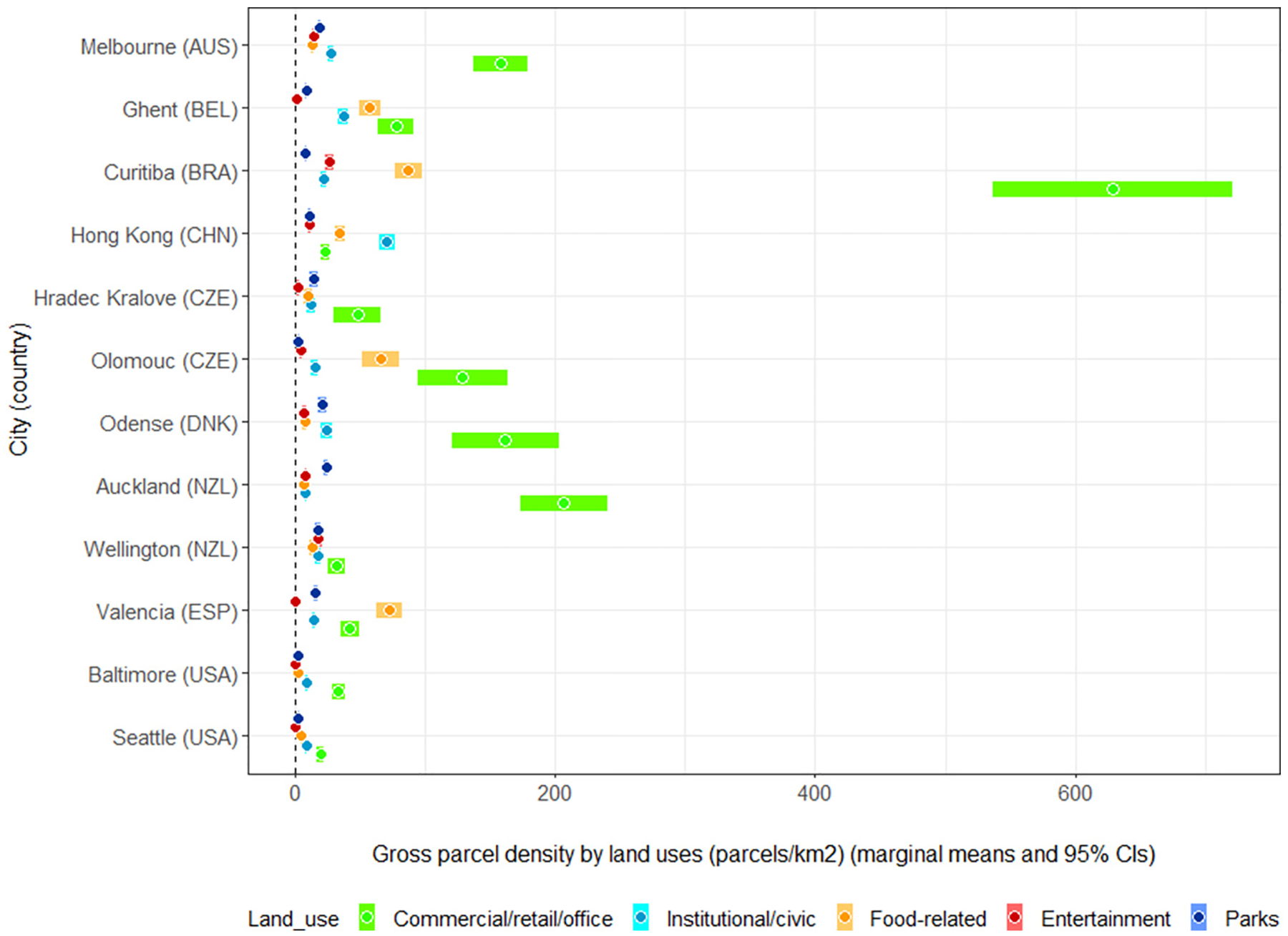
Gross parcel density by land use (parcels/km^2^) and study site.

Fig. 3 shows the marginal means and 95 % CIs of gross parcel density by non-residential land use (parcels/km^2^) for each city, adjusted for neighbourhood type (walkability by SES categories). In all cities, except for Hong Kong (CHN) and Valencia (ESP), parcel density was the highest for commercial/retail/office land use (Fig. 3; Table S1, Supplementary Material). Food-related and institutional/civic land uses were the next most prevalent parcel types. The highest density of food-related parcels was observed in Ghent (BEL), Curitiba (BRA), Olomouc (CZE) and Valencia (ESP), while Hong Kong (CHN) and Ghent (BEL) had relatively high densities of institutional/civic parcels. Entertainment and park parcels had the lowest density in most cities. Park density was the highest in Melbourne (AUS), Hradec Kralove (CZE), Odense (DNK) and Auckland (NZL) (Fig. 3; Table S1, Supplementary Material).

**Fig. 4.**
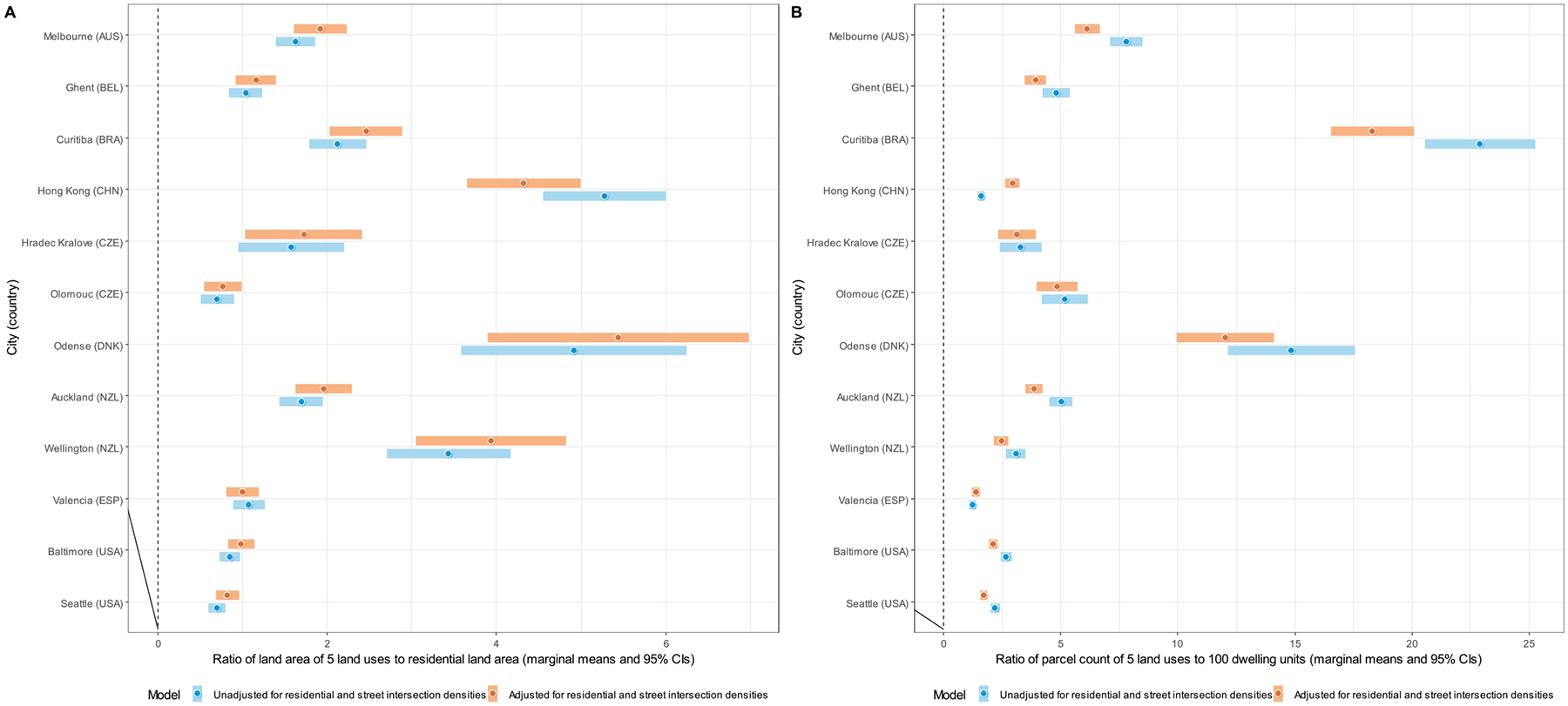
Marginal means of the ratio of land area of 5 non-residential land uses to residential land area (DLUM 1) (panel A) and ratio of parcel count of 5 non-residential land uses to 100 dwelling units (DLUM 2) (panel B) by study site, adjusted and unadjusted for net residential density and street intersection density.

**Fig. 5.**
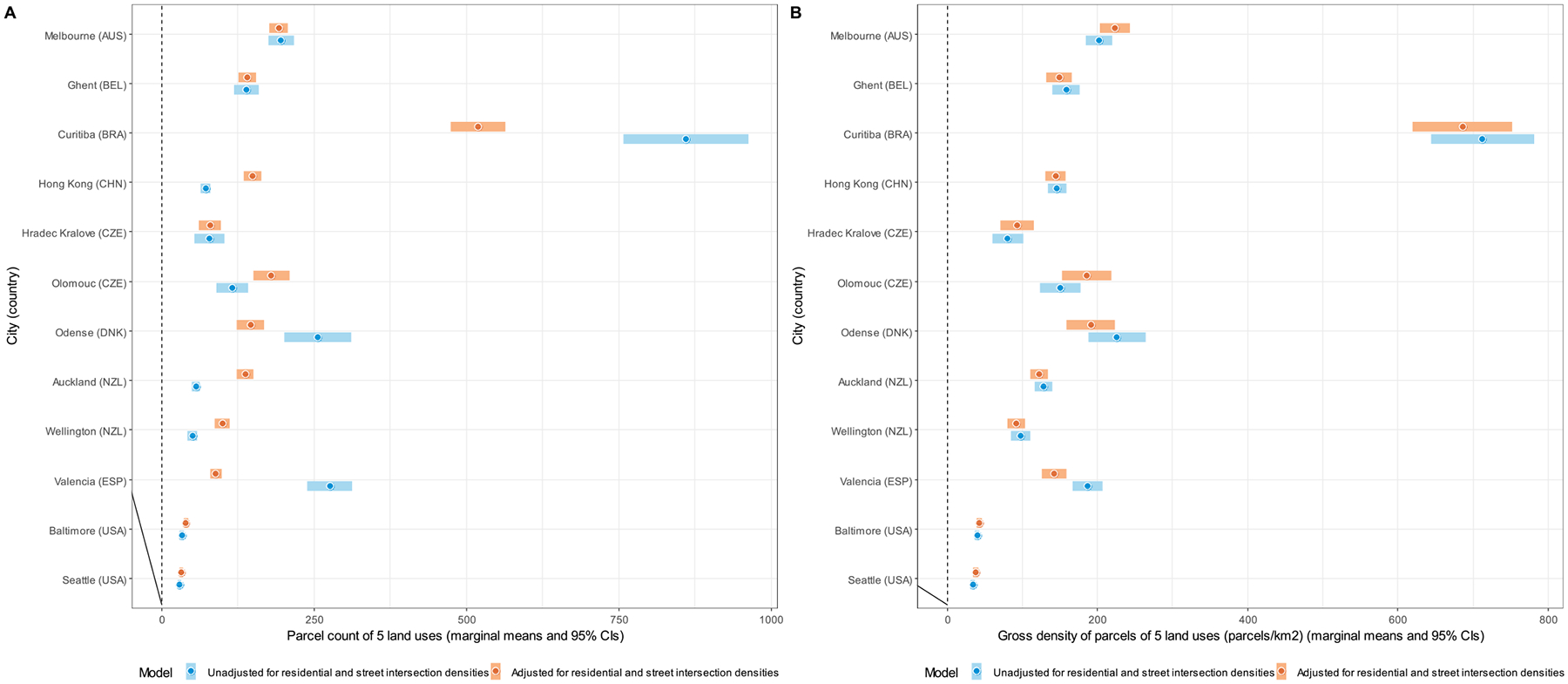
Marginal means of parcel counts of 5 non-residential land uses (DLUM 3a) (panel A) and gross density of parcels of 5 non-residential and uses (parcels/km^2^) (DLUM 3b) (panel B) by study site, adjusted and unadjusted for net residential density and street intersection density.

**Fig. 6.**
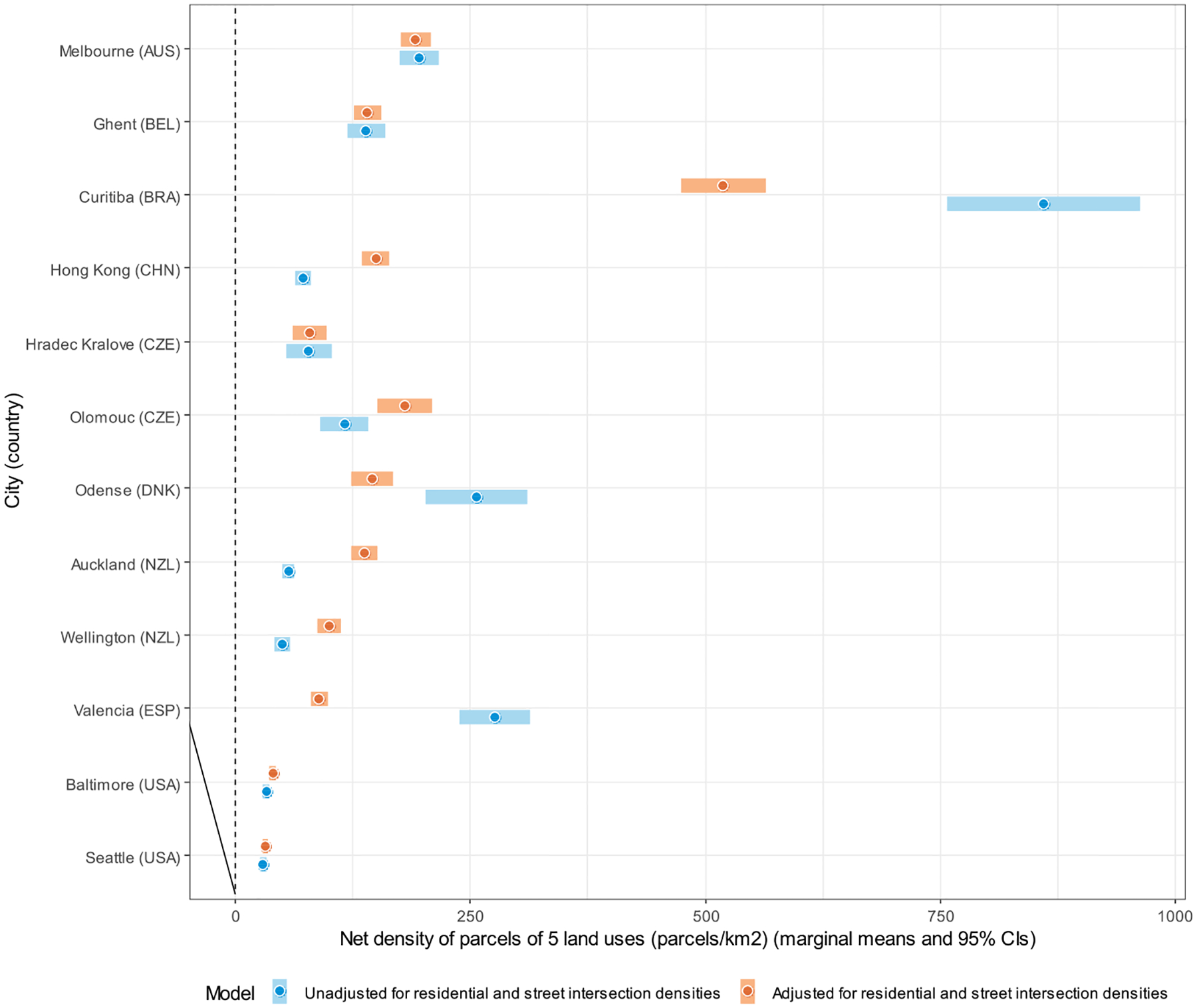
Marginal means of net density of parcels of 5 non-residential land uses (parcels/km^2^) (DLUM 4) by study site, adjusted and unadjusted for net residential density and street intersection density.

**Fig. 7.**
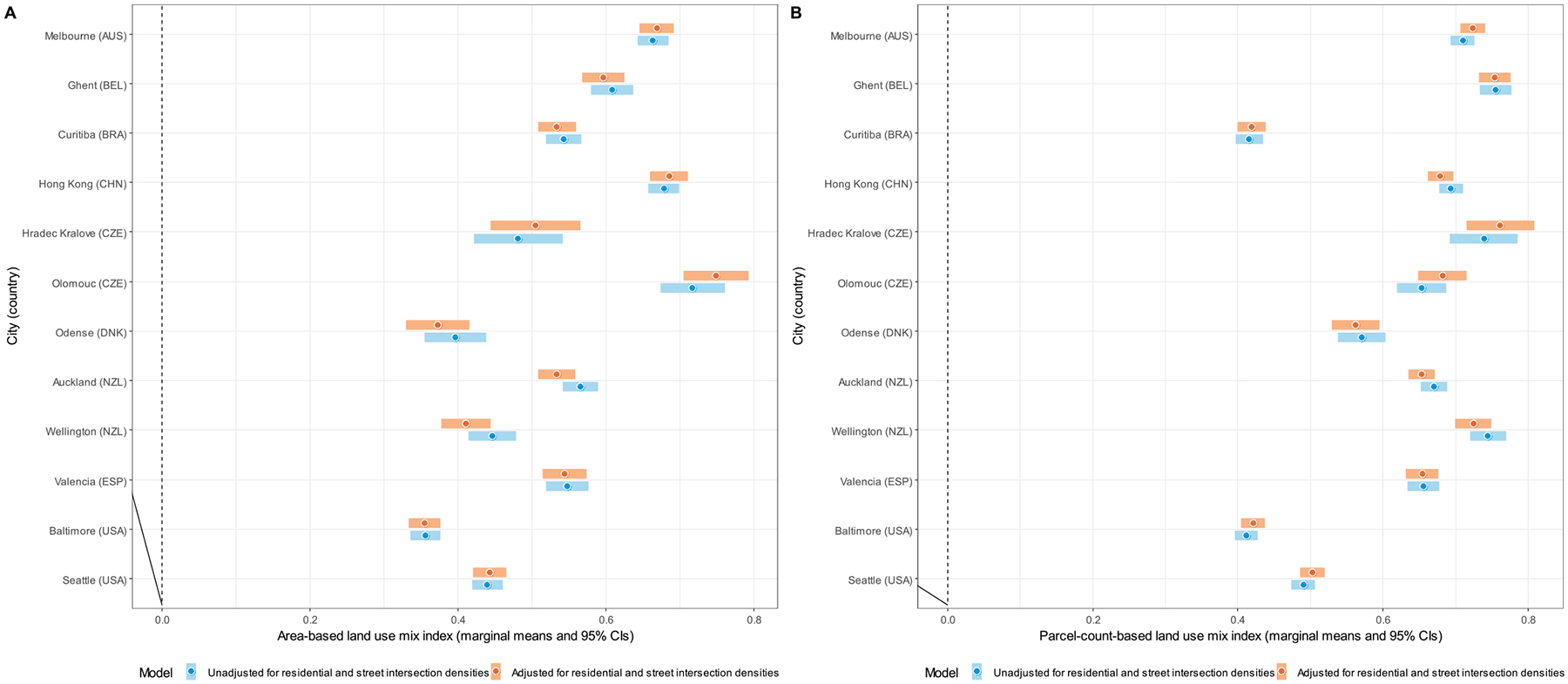
Marginal means of area-based (DLUM 5) (panel A) and parcel-count-based land use mix index (DLUM 6) (panel B) by study site.

**Fig. 8.**
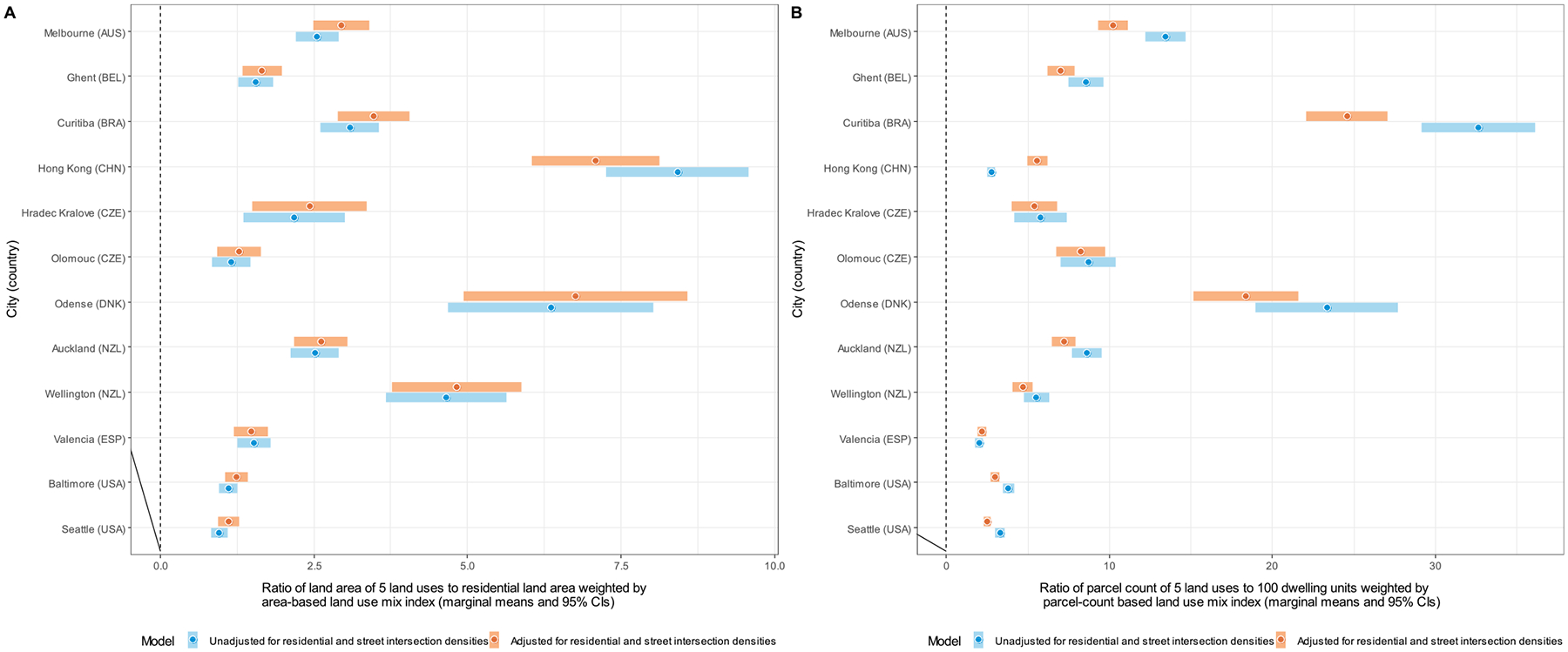
Marginal means of the ratio of land area of 5 non-residential land uses to residential land area weighted by the area-based land use mix index (DLUM 7) (panel A); and ratio of parcel count of 5 non-residential land uses to 100 dwelling units weighted by the parcel-count based land use mix index (DLUM 8) (panel B) by study site, adjusted and unadjusted for net residential density and street intersection density.

**Fig. 9.**
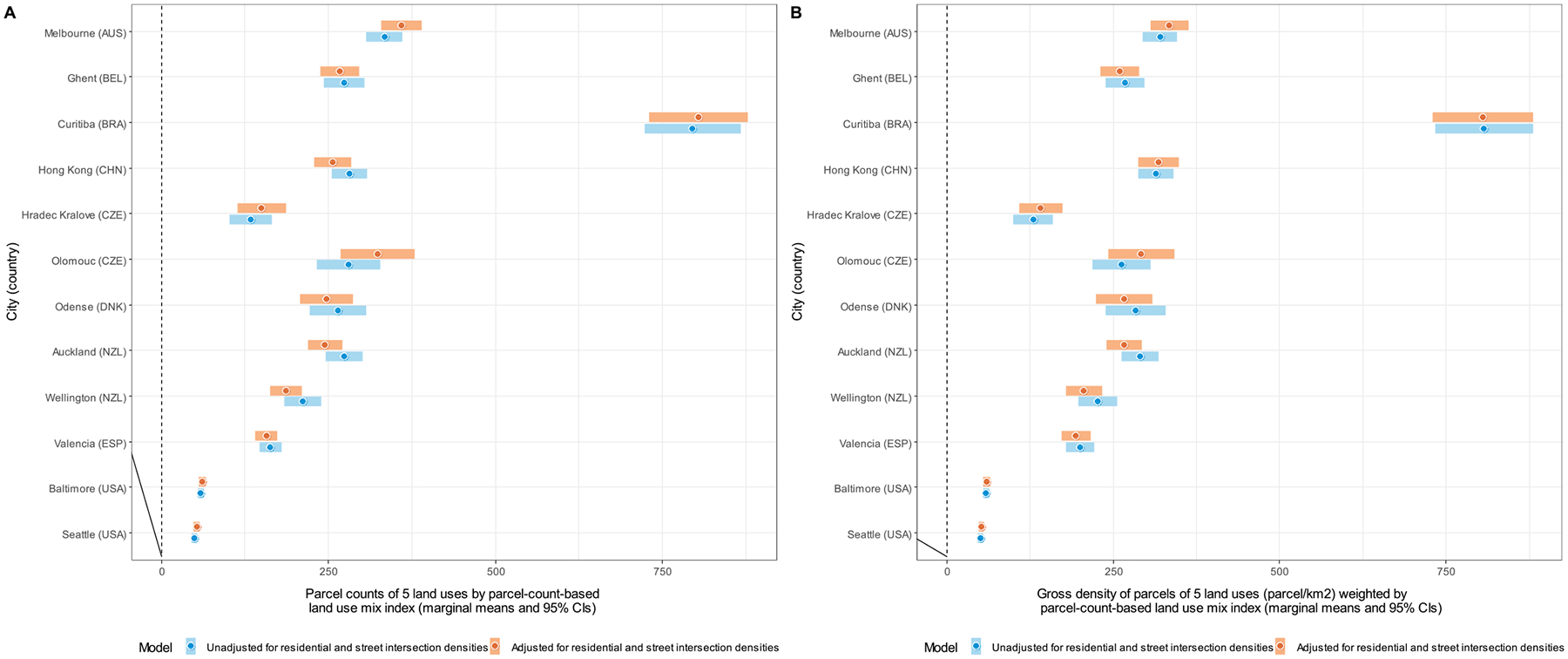
Marginal means of parcel counts of 5 non-residential land uses (DLUM 9a) (panel A) and gross density of parcels of 5 non-residential land uses (parcels/km^2^) (DLUM 9b) (panel B) weighted by parcel-count-based land use mix index by study site, adjusted and unadjusted for net residential density and street intersection density.

**Fig. 10.**
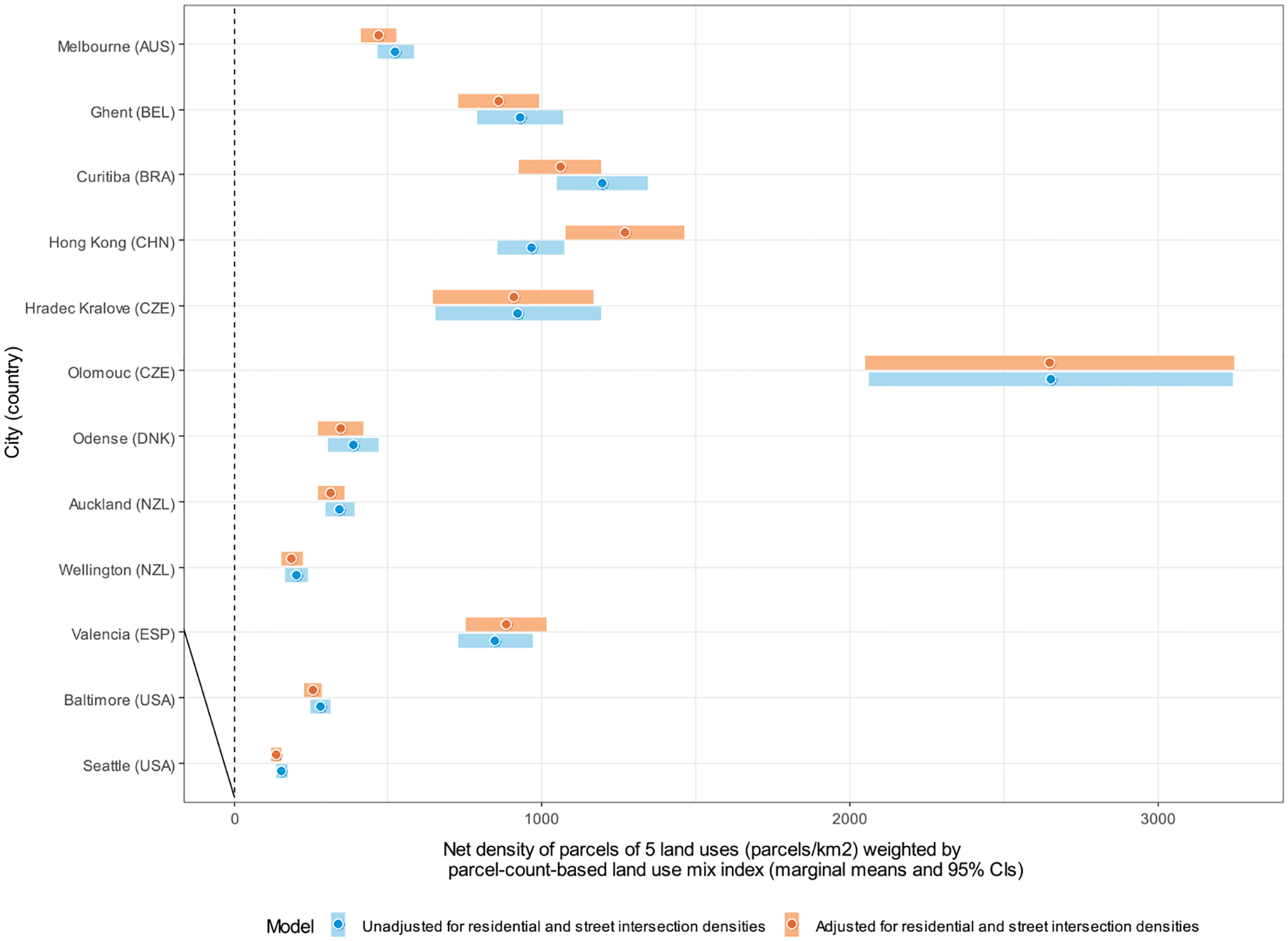
Marginal means of net density of parcels of 5 non-residential land uses (parcels/km^2^) weighted by parcel-count-based land use mix index (DLUM 10) by study site, adjusted and unadjusted for net residential density and street intersection density.

Site-specific marginal means of DLUM indicators unadjusted and adjusted for residential and street intersection densities are depicted in Figs. 4–10, and site rankings for each indicator (unadjusted for residential and street intersection densities) are reported in Table 4. Correlations between the indicators can be found in Fig. S2 (Supplementary Material). We observed significant between-site differences across all indicators (Figs. 4–10) and substantial between-indicator differences in study site rankings (Table 4). The three best performing sites based on average rankings were Melbourne (AUS), Curitiba (BRA) and Hong Kong (CHN), although the ranking of the latter two varied substantially across indicators. Baltimore and Seattle (USA) ranked low on all indicators. Curitiba (BRA) performed well on indicators based on parcel counts but performed poorly on indicators of heterogeneity of land uses (land use mix indices; DLUM 5 and 6). Melbourne (AUS) performed well on indicators of heterogeneity of land uses (DLUM 5 and 6) as well as those based on parcel counts, with the exception of net density of parcels of 5 land uses (DLUM 4 and 10). Hong Kong (CHN) performed relatively well on all indicators except for those solely based on number of parcels (DLUM 3a and 3b) and ratio of non-residential parcels to dwelling units (DLUM 2 and 8). Hong Kong (CHN) outperformed other sites on indicators of ratio of non-residential to residential land area (DLUM 1 and 7). Ghent (BEL), Hradec Kralove (CZE) and Olomouc (CZE) had relatively high values on land use mix indices (DLUM 5 or 6) but lower average ratios of non-residential to residential land area (DLUM 1 and 7). Odense (DNK) performed well on ratio measures of non-residential to residential areas or units (DLUM 1–4, 7 and 8) but underperformed on measures of land use heterogeneity (DLUM 5 and 6). Wellington (NZL) had high average parcel-count based land use mix (DLUM 6) and ratios of non-residential to residential area (DLUM1 and 7). However, it performed poorly on indicators based on non-residential parcel counts (DLUM 2–4, 8–10). In contrast, Valencia (ESP) performed well on indicators based on non-residential parcel counts except for the ratios of non-residential parcel counts to dwelling units (DLUM 2 and 8). Of note is that weighting by a parcel-count-based land use mix index resulted in Melbourne (AUS), Hong Kong (CHN), Olomouc (CZE) and Wellington (NZL) improving their rankings on the parcel counts or density of 5 land uses indicators. The opposite was observed for Odense (DNK) and Valencia (ESP).

Net residential and street intersection densities explained between-site differences in eight and nine of 12 DLUM indicators, respectively (Table S2, Supplementary Material). Sites with higher street intersection density tended to have higher values on all indicators except ratio of land area of 5 land uses to residential land area (DLUM 1), ratio of parcel counts of 5 land uses to dwelling units (DLUM 2) and net density of parcels of 5 land uses unweighted and weighted by parcel-count-based land use mix index (DLUM 4 and 10). Sites with higher net residential density tended to have higher values on only four indicators (DLUMs 1, 6, 7 and 9a) and lower values on four other indicators (DLUMs 2, 4, 8 and 10). Figs. 4–10 show the changes in marginal means and 95 % CIs of the indicators after adjustment for net residential and street intersection densities across study sites.

### 5.2. Associations of GIS-based destination and land-use mix indicators with parental perceived proximity of destinations from home

Associations of ratios of land area of 5 non-residential land uses to residential area indicators unweighted and weighted by area-based land use mix (DLUM 1 and 7) with parental perceived proximity of destinations (overall score) varied by study site. They were mostly nil, tended to be negative in Melbourne (AUS) and positive in Ghent (BEL), Olomouc (CZE) and Odense (DNK) (see Tables S3 and S4, Supplementary Material). Between-site differences were also found in associations of net density of parcels of 5 land uses with parental perceived proximity to destinations irrespective of weighting by land use mix (DLUM 4 and 10) (Table S3; Figs. S3 and S4; Supplementary Material). These associations varied in shape as well as direction (Figs. S3 and S4; Supplementary Material).

**Fig. 11.**
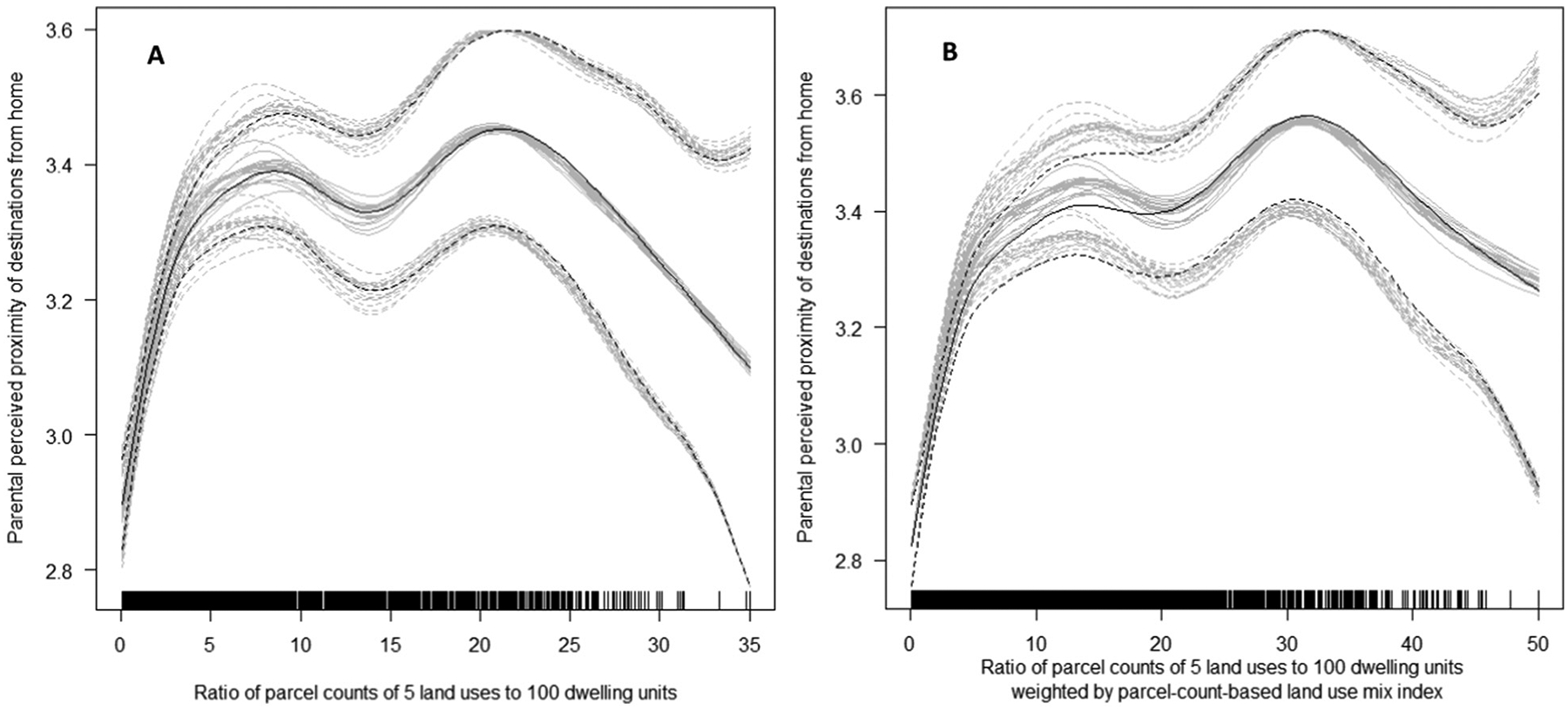
Associations of ratio of parcel count of 5 non-residential land uses to 100 dwelling units unweighted (DLUM 2) (panel A) and weighted (DLUM 8) (panel B) by parcel-count based land use mix index with parental perceived proximities of corresponding destinations.

**Fig. 12.**
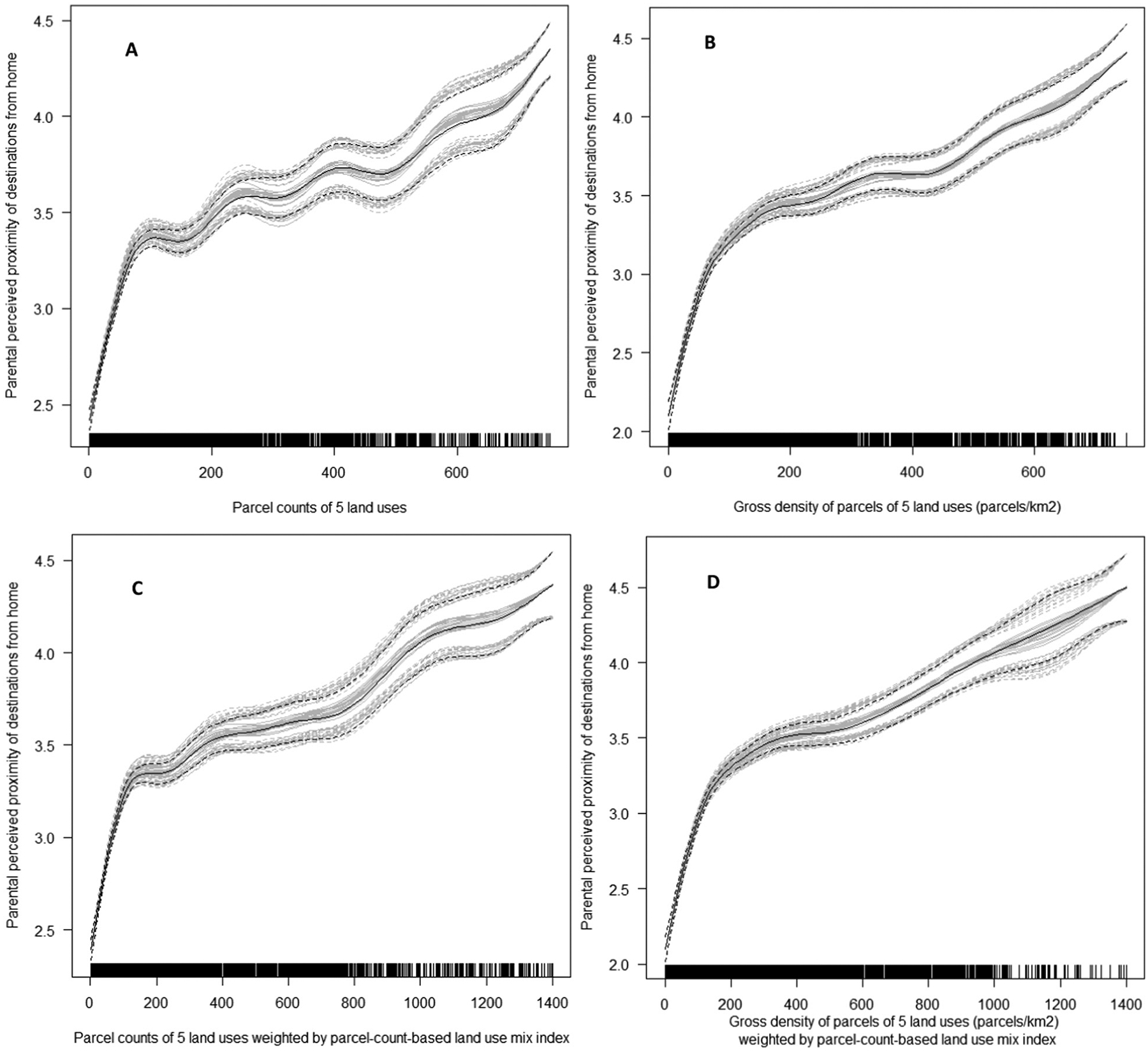
Associations of parcel count and gross density of 5 non-residential land uses unweighted (DLUM 3a and 3b) (panels A and C) and weighted (DLUM 9a and 9b) (panels B and D) by parcel-count based land use mix index with parental perceived proximities of corresponding destinations.

For the remaining GIS-based DLUM indicators, the direction and shape of associations with parental perceived proximity of destinations did not significantly differ by site. Inverted-U and inverted-J associations were found for the ratio of parcel count of 5 land uses to dwelling units indicators (Table S3, Supplementary Material; DLUM 2 and 8) (Fig. 11), while the associations for parcel count and gross parcel density indicators were also curvilinear but positive (Table S3, Supplementary Material; Fig. 12). Associations of land use mix indices (DLUM 5 and DLUM 6) with parental perceived proximity of destinations were linear and positive but differed in strength across sites (Table S5; Supplementary Material). GIS-based gross densities of parks and commercial/retail/office, institutional/civic and food-related land use parcels were positively related to their respective parent-reported proximity measures, although associations were curvilinear and stronger at lower levels of density (Fig. S5; Supplementary Material).

### 5.3. Associations of GIS-based destination and land-use mix indicators with adolescents’ physical activity

The ratios of land area of 5 non-residential land uses to residential land area weighted and unweighted for area-based land use mix index (DLUM 1 and 7) were negatively associated with accelerometer-based total MVPA, regular walking and overall active transport to/from school (Table 5). The unweighted ratio of parcel counts of 5 land uses to dwelling unit count (DLUM 2) showed more heterogeneous associations with adolescents’ physical activity, ranging from positive with respect to self-reported total non-school physical activity (Table 5) to negative in relation to active transport to school in adolescents reporting 2–4 days per week of 60 min of non-school physical activity (Table 6). Associations with non-school active transport varied by study sites (Table S6; Supplementary Material). Fewer associations were observed for the weighted version of this indicator (DLUM 8) (Table 5 and Table S6; Supplementary Material).

**Fig. 13.**
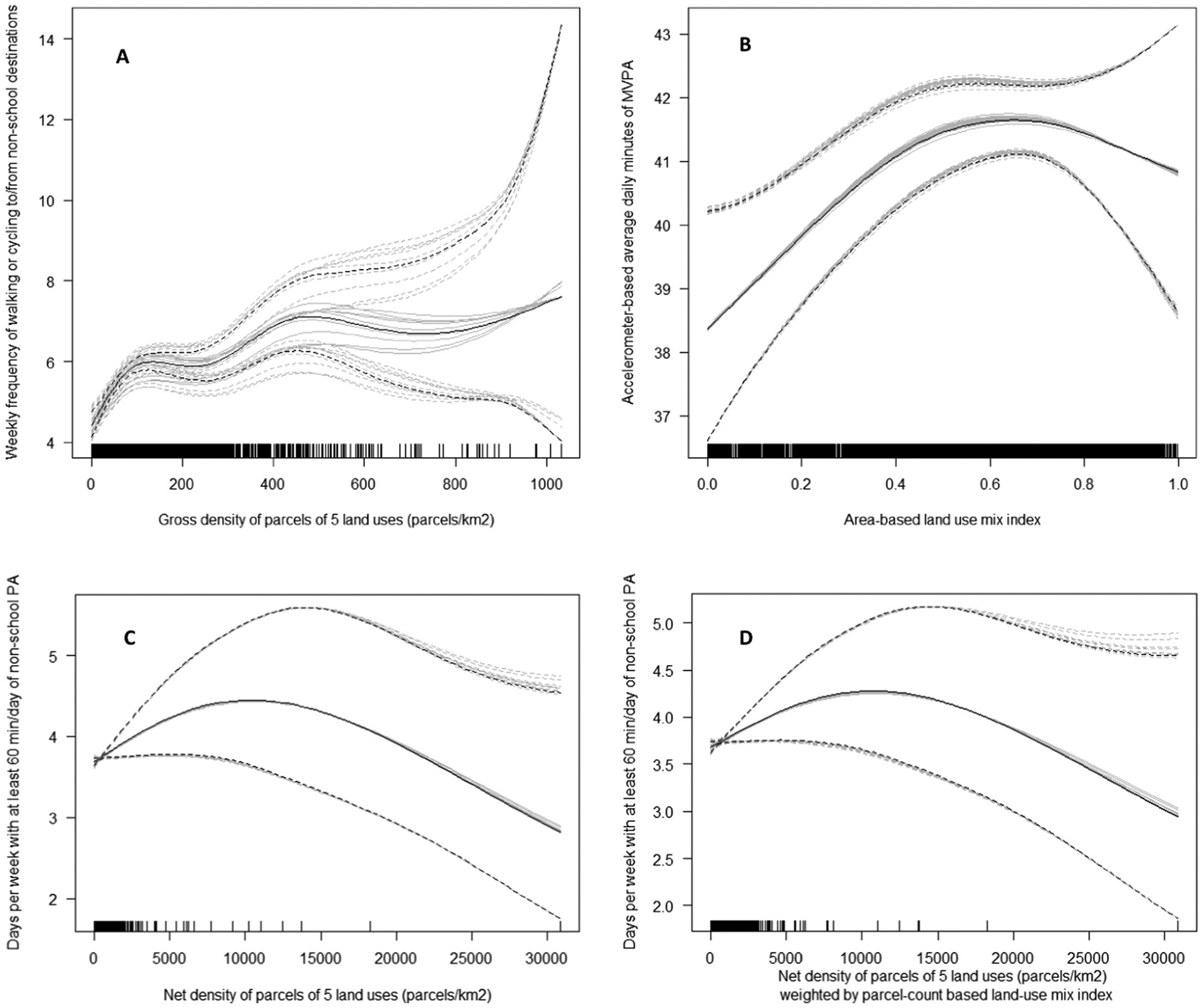
Curvilinear associations of destination and land-use mix (DLUM) indicators with adolescents’ physical activity (PA). Gross density of parcels of 5 non-residential land uses (DLUM 3b) and non-school active transport (panel A); area-based land use mix index (DLUM 5) and accelerometer-based total moderate-to-vigorous PA (panel B); net density of parcels of 5 non-residential land uses unweighted (DLUM 4) and weighted (DLUM 10) by parcel-count based land use mix index and self-reported total non-school PA (panels C and D).

The land-use-mix unweighted and weighted counts and density of parcels of 5 land uses (DLUM 3a, 3b, 9a and 9b) showed similar positive associations with measures of active transport and accelerometer-based MVPA (Tables 5 and 6). The density of parcels indicators (DLUM 3b and 9b) differed from their parcel count counterparts (DLUM 3a and 9a) in associations with accelerometer-based MVPA during non-school periods, with the former (b versions) but not the latter (a versions) showing significant positive associations (Table 5). Land-use-mix weighted density of parcels (DLUM9 b) showed more parsimonious (simpler) effects with physical activity outcomes than its unweighted counterpart (DLUM 3b). Specifically, the weighted indicator showed a linear rather than curvilinear relation with non-school active transport (Table 5 and Fig. 13A), and its association with regular walking to/from school was not moderated by self-reported total non-school physical activity (Tables 5 and 6).

Indicators of net density of parcels of 5 land uses (DLUM 4 and DLUM 10) were unrelated to accelerometer-based MVPA outcomes as well as walking and overall active transport to/from school (Table 5). They were linearly positively related to non-school active transport in Seattle (USA) only (see Table S6; Supplementary Material) and displayed inverted-U relations with total non-school physical activity (Fig. 13; panels C and D). The two land use mix indices (DLUM 5 and 6) were positively related to all measures of active transport (Table 5) irrespective of the study locations. However, the association between the area-based land use mix index and the odds of regular active transport to/from school depended on the distance between the adolescents’ home and their school. The association was significant only if their school was within a 30-min walk from home (Table S8, Supplementary Material). The area-based LUM index (DLUM 5) was positively curvilinearly related to total accelerometer-based MVPA (Fig. 13B), but the parcel-count-based counterpart (DLUM 6) was negatively associated with adolescent-reported total non-school physical activity.

Associations of gross densities of specific land use parcels (e.g., parks or commercial/retail/office parcels) with physical activity outcomes are reported in Tables S9–S11 and Fig. S6 (Supplementary Material). Gross density of commercial/retail/office parcels was positively associated with accelerometer-based total MVPA and non-school active transport (Table S9; Supplementary Material). Density of institutional/civic parcels was positively associated with the same physical activity outcomes (Table S9 and Fig. S6A; Supplementary Material) and self-reported non-school total physical activity (Table S9; Supplementary Material). Density of food-related parcels was curvilinearly positively related to non-school active transport (Fig. S6B; Supplementary Material) and both regular walking and active transport to/from school outcomes, but only if the school was within a 10-to-20-min walk from home (Table S10; Supplementary Material). Density of parks was negatively related to regular walking to/from school (Table S9; Supplementary Material). Density of parks also showed mixed associations with non-school active transport across study sites (Table S11; Supplementary Material). Finally, density of entertainment land use parcels was unrelated to physical activity outcomes (Table S9; Supplementary Material).

## 6. Discussion

Using data from 12 study sites across nine geographically and culturally diverse countries, we examined the cross-site comparability and utility of 12 GIS-based indicators of destination accessibility in explaining adolescents’ physical activity. We considered indicators capturing either, as well as both, intensity and heterogeneity of destinations/land uses. Unless representing the LUM weighted version of the same intensity indicator, most DLUM indicators were only weakly to moderately intercorrelated and yielded substantially different rankings of study sites, suggesting the various GIS-based indicators may capture different aspects of destination accessibility. The best performing DLUM indicators, defined as being consistent correlates of parental perceptions of destination accessibility and adolescents’ physical activity in the expected direction, were gross density of non-residential destinations weighted by the parcel-count-based LUM index (DLUM 9b) and the two LUM indices (DLUM 5 and 6). Below we provide a detailed discussion of the findings by type of DLUM indicator and recommendations for their implementation.

### 6.1. Ratios of non-residential to residential land/destinations

DLUM indicators based on ratios of non-residential land/parcels to residential land/dwelling units (DLUM 1, 2, 7 and 8) showed sometimes counterintuitive, contrasting, and/or site-dependent associations with adolescents’ physical activity and parental perceptions of destination accessibility. The ratios of non-residential to residential land (DLUM 1 and 7) performed particularly poorly. They showed positive associations with parent-perceived destination accessibility in only three out of 10 study sites and were consistently negatively related to adolescents’ active transport to/from school and total accelerometer-based MVPA. The inclusion of parks in non-residential land may have contributed to the observed findings for two reasons. Firstly, parks tended to be negatively associated with both active transport and total accelerometer-based MVPA. Secondly, if present in the neighbourhood, parks may take up a substantial proportion of the total area yielding a relatively large non-residential to residential land ratio. While the exclusion of parkland from non-residential land might have resulted in more theoretically plausible findings, we opted for its inclusion to account for the fact that parks have been identified as places that enable physical activity in adolescents (McGrath et al., 2015). Overall, based on the patterns of associations observed in this study, we do not recommend ratios of non-residential to residential land (as operationalised in this study; DLUM 1 and 7) be used as indicators of destination accessibility in an international context and especially in physical activity research in adolescents.

Ratios of non-residential parcels to dwelling units (DLUM 2 and 8) showed slightly more promising results. Although inverted-U shaped, their associations with parent-perceived destination accessibility were consistent across study sites. These DLUM indicators were also consistently positively associated with adolescent total non-school physical activity (i.e., reported number of days/week of ≥60 min of non-school physical activity). However, their relations with active transport outcomes varied either by site or amount of self-reported total non-school physical activity and, contrary to expectations, ranged from negative to positive. It is also noteworthy that these DLUM indicators were negatively rather than positively related to residential density (Table S2, Supplementary Material), which might help explain their positive association with non-school (presumably leisure) physical activity. Systematic reviews, for example, report negative associations between residential density and leisure-time physical activity in adolescents (Lambert et al., 2019; Schüle & Bolte, 2015), which have been attributed to less-dense urban areas having better traffic safety (Lambert et al., 2019) and more space for sports fields and other community-based physical activity destinations (Slater et al., 2010). In line with the latter conjecture and reports of positive associations between physical activity facilities and adolescents’ participation in sports and leisure-time physical activity (Morton et al., 2016; Schüle & Bolte, 2015), we found a positive association of gross density of institutional/civic destinations - some of which would represent sporting facilities within schools, community centres and youth clubs - with non-school physical activity (Table S9; Supplementary Material). As ratios of non-residential parcels to dwelling units (DLUM 2 and 8) appear to be better proxies of low residential density than high destination accessibility, especially in dense urban environments such as those found in some European and Asian cities (Table 4 and Fig. 4B), we do not recommend they be used as indicators of destination accessibility in international research.

### 6.2. Count and density of non-residential destinations

In this study, we examined count (DLUM 3a and 9a), gross density (DLUM 3b and 9b) as well as net density (DLUM 4 and 10) of non-residential parcels as correlates of adolescents’ physical activity. While previous studies had mainly focused on counts and gross density (Prince et al., 2022a), we hypothesised the density of non-residential destinations within non-residential land (i.e., net density) may be a valuable indicator of conveniently clustered multistorey multi-purpose destinations that may promote physical activity, especially non-school active transport. Yet, present analyses did not provide support for our hypothesis as, firstly, indicators of net density of non-residential parcels were inconsistently, and sometimes negatively, associated with parental perceptions of destination accessibility across sites; and, secondly, indicators were positively related to adolescents’ non-school active transport only in one low-density location (Seattle, USA). Weak curvilinear associations were observed between these indicators and adolescent-reported total non-school physical activity, which were positive only at lower values of the indicators. Similarly to the indicators of ratios of non-residential parcels to dwelling units (DLUM 2 and 8), net density of non-residential parcels (DLUM 4 and 10) seemed to misrepresent destination accessibility in denser urban environments. In fact, apart from being negatively associated with both net residential and street intersection densities, these DLUM indicators tended to be inversely related to parental perceptions of destination accessibility in three of the four most-dense cities in this study: Hong Kong (CHN), Hradec Kralove (CZE), and Olomouc (CZE) (Figs. S3 and S4, Supplementary Material).

The more commonly used count (DLUM 3a and 9a) and gross density of non-residential parcels (DLUM 3b and 9b) proved to be better indicators of destination accessibility as well as correlates of adolescents’ physical activity. Unlike net density indicators (DLUM 4 and 10), they were not inversely related to residential density and showed consistent positive associations with parental perceptions of destination accessibility across all sites. This pattern of findings suggests these indicators have the same or similar meaning across different geographical and cultural contexts and are, thus, appropriate measures of destination accessibility for international studies. In terms of their relevance for adolescents’ physical activity, they both showed positive associations with measures of active transport to/from school as well as other destinations, although the former sometimes depended on the level of adolescents’ engagement in self-reported non-school physical activity. Importantly, associations with physical activity outcomes did not differ by study site, except for total accelerometer-based MVPA which was positively related to these DLUM indicators only in Australia and the USA.

Previous studies reported positive associations of similar DLUM indicators with total physical activity (Davison & Lawson, 2006; Nordbø et al., 2020; Prince et al., 2022a), and active transport (Prince et al., 2022a; Timperio et al., 2025) in youth, although the findings specific to adolescents were mixed. It is interesting that active transport to/from school, but not other destinations, showed a positive association with count and gross density of non-residential parcels that depended on the level of participation in non-school physical activity in the present study. Adolescents who engage in non-school physical activity are likely to do so after school and, for this reason, they may not be able to afford the time to walk or cycle back home (Campos-Garzón et al., 2023). Having a variety of destinations along the route from school to home may be irrelevant to them. In contrast, adolescents who do not participate in after-school sports or leisure activities may afford the time to walk or cycle back home and be more likely to do so if they have a variety of destinations they may visit with their peers or friends on their way home (Timperio et al., 2025).

When comparing count vs. gross density DLUM indicators, the latter might be preferred because they provide a measure that is to a lesser extent influenced by features of the street network. A neighbourhood with a more connected street network may have the same gross density but a higher count of non-residential parcels than a less connected neighbourhood due to its street network extending to a larger area within a fixed distance from a residential address. Although it could be argued a measure that captures street connectivity as well as availability of destinations may be a superior measure of destination accessibility, we maintain it might be better to use measures that distinguish destination availability or intensity from street connectivity to better estimate their independent impacts on physical activity. Moreover, in this study, gross density, but not count, of non-residential parcels was positively associated with accelerometer-based MVPA during non-school periods. This association did not differ across study sites, making gross density of non-residential parcels (DLUM 3b and 9b) DLUM indicators particularly suitable for international comparisons of destination accessibility and studies of adolescents’ physical activity.

### 6.3. Heterogeneity of land use/destinations

Both intensity and heterogeneity indicators of destination accessibility are potentially important determinants of an active lifestyle (Giles-Corti et al., 2009; Saelens et al., 2003; Transportation Research Board, 2005). Having diverse types of destinations within walking distance from home makes it more likely that residents will regularly walk for utilitarian purposes. In this study, heterogeneity was operationalised using two LUM entropy indices, one based on land area data (DLUM 5) and the other based on parcel data (DLUM 6). Both area- and parcel-count-based LUM indices were, as expected, positively related to parental perceptions of destination accessibility and the strongest correlates of adolescents’ active transport. The parcel-count-based index outperformed its area-based counterpart in its associations with adolescents’ active transport to/from school and parental perceptions of destination accessibility by displaying more consistent patterns of findings across sites and distances to school. It is easily conceivable that the number and variety of types of non-residential parcels in a neighbourhood would more closely mirror residents’ perceptions of destination diversity and accessibility and, hence, be more strongly related to active transport. In fact, parcels approximate the presence of distinct destinations better than land area does. However, present findings suggest the area-based non-residential LUM entropy index may have some advantages over its parcel-count-based counterpart because it was positively, albeit curvilinearly, related to accelerometer-based total MVPA and was not inversely related to adolescent-reported non-school physical activity. These differences in findings between the two indices may be attributable to the parcel-count-based, but not the area-based, index being positively associated with residential density. As noted earlier, dense, compact neighbourhoods may not be as conducive to participation in sports and other active leisure-time pursuits as low-density neighbourhoods due to traffic safety concerns and the lack of space for recreational facilities (Lambert et al., 2019; Slater et al., 2010).

Previous studies that examined GIS-based indicators of destination heterogeneity as correlates of adolescents’ physical activity used exclusively area-based LUM indices (Ding et al., 2011; Panter et al., 2008). They reported positive associations with total self-reported physical activity (Ding et al., 2011) but mixed findings with device-assessed physical activity (Ding et al., 2011) as well as transport-related physical activity (Panter et al., 2008). However, prior studies were almost exclusively conducted in single geographical locations with limited environmental variability and, therefore, limited power to detect associations (if present). Our findings are more promising and suggest neighbourhoods with mixed land uses/destinations may help adolescents be physically active. Considering the observed patterns of associations, we recommend the newly proposed parcel-count-based LUM index (DLUM 6) be used in research focusing on adolescents’ active transport and the area-based LUM index (DLUM 5) be used in studies of adolescents’ leisure-time or total physical activity.

### 6.4. Hybrid measures of intensity and heterogeneity of land use/destinations

In addition to the newly developed parcel-count-based LUM index, the present study examined a set of novel DLUM indicators that capture both intensity and heterogeneity of land use/destinations. We hypothesised these hybrid intensity + heterogeneity DLUM indicators would yield stronger and/or more consistent associations with adolescents’ physical activity and parent-perceived destination accessibility than simpler DLUM indicators of land use/destination intensity. However, no substantive differences in patterns of associations were found between the heterogeneity-weighted and unweighted ratios of non-residential to residential land (DLUM 1 and 7), counts (DLUM 3a and 9a) and net density (DLUM 4 and 10) of non-residential parcels. In contrast, the heterogeneity-weighted versions of the remaining DLUM indicators performed better than their unweighted versions. Specifically, the weighted ratio of non-residential parcels to dwelling units (DLUM 8) showed a slightly more theoretically plausible association with parent-perceived access to destinations (Fig. 11) than the respective unweighted indicator (DLUM 2). Gross density of non-residential destinations weighted by the parcel-count-based LUM index (DLUM 9b) displayed more similar associations with parent-perceived destination accessibility across study sites than its unweighted version (DLUM 3b). Additionally, the relations of these two heterogeneity-weighted DLUM indicators with physical activity were more parsimonious in terms of shape (linear rather than curvilinear) and sensitivity to moderating factors (less dependent on moderators and generalisable across groups). Overall, these findings suggest that DLUM indicators capturing both intensity and heterogeneity of land use/destinations (e.g., DLUM 9a and 9b) may be slightly more useful measures of destination accessibility than DLUM indicators of intensity (e.g., DLUM 3a and 3b). Thus, we recommend DLUM indicators that combine intensity and heterogeneity for further evaluation and use in studies of the relation of built environments to physical activity and other outcomes.

### 6.5. Differences across cities/regions: reflections and practical implications

Our study was conducted in 12 cities/regions differing in residential density and street connectivity, as well as cultural contexts, providing an opportunity to reflect on how three components of neighbourhood walkability – namely, density, connectivity and destination accessibility - vary across geographical locations and relate to residents’ perceptions and adolescents’ physical activity behaviours in diverse cultural contexts. One important finding was the weak associations among study-site averages of the three components of walkability, suggesting that, at the city level, these attributes are relatively independent. For example, although Hong Kong (China) had by far the highest average net dwelling density among the selected cities/regions, its average street intersection density was lower than that of low-density cities such as Auckland and Wellington (New Zealand). Also, cities differed more on density than connectivity.

As to destination accessibility, we found substantial between-site differences that depended on the type of spatial indicator used. Although the two US locations (Baltimore and Seattle) performed poorly on all indicators and Melbourne (Australia) on none, the performance of the remaining cities ranged from poor (lowest tertile) to good (top tertile) depending on the destination accessibility indicator used. If we consider only the spatial indicators we recommend for international research (DLUM 5, 6 and 3b/9b), Ghent (Belgium) and Hong Kong (China) showed relatively consistent good performance. It is interesting that among the cities excelling in destination accessibility, we found culturally diverse locations across the whole density spectrum – from low density cities (Melbourne, Australia) to medium (Ghent, Belgium) and high density (Hong Kong, China) cities. This finding means that, at the city level, density and destination accessibility, as measured by our recommended indicators, are relatively independent components of walkability. Moreover, our study suggests the intensity and heterogeneity dimensions of destination accessibility are also relatively independent of each other at the within-city as well as between-city level. For example, Curitiba (Brazil) and Odense (Denmark) had high average values on spatial indicators of intensity but low average values on indicators of heterogeneity relative to other locations, and Ghent (Belgium) showed the opposite pattern. It is, thus, unsurprising that the ranking of cities/regions by gross density of non-residential destinations differed from that of the corresponding indicator weighted by a parcel-count-based LUM index (DLUM 9b). Cities that showed a substantial improvement in ranking when using the hybrid intensity + heterogeneity indicator were Hong Kong (China) and Auckland (New Zealand), while Odense (Denmark) and Valencia (Spain) lost, respectively, three and five positions on the ranking list.

Apart from observing anticipated between-site differences in characteristics of the built environment, we also found between-site differences in associations of some spatial indicators of neighbourhood destination accessibility and adults’ (parental) perceptions of destination accessibility. Of note is that the parcel-count-based LUM index (DLUM 6), which is one of the indicators we recommend, was less strongly related to parental perceptions of destination accessibility in Melbourne (Australia) and the two US cities (Baltimore and Seattle), all English-speaking regions with the highest rates of motor vehicles per capita (Fig. S1). The strongest associations were observed in Hong Kong (China) and Valencia (Spain), the two most densely populated cities with warmer climates and low-to-moderate rates of motor vehicles per capita (Fig. S1). Differences in active transport across these geographical locations [40 % of trips in Hong Kong (Hung, 2010), 58 % in Valencia (Phoebe Project, 2024), 14 % in Melbourne (Garrard, 2009), 9.0 % in Seattle (Gilmore Research Group, 2011), and 7.3 % in Baltimore (Westat., 2020)] may be responsible for the observed findings, given that walking and cycling provide an opportunity for individuals to develop a better awareness of their local environments (Kirtland et al., 2003).

As to adolescents’ physical activity, associations of spatial indicators of destination accessibility did not vary by city/region for self-reported non-school leisure physical activity, active transport to/from school, and accelerometry-based MVPA during non-school periods, indicating associations generalised across geographical and cultural locations. Non-school active transport showed site-specific associations with spatial indicators that displayed highly diverse relationships with perceived destination accessibility. Accelerometry-based total MVPA was the only physical activity measure presenting site-specific associations with one set of recommended spatial indicators, gross density of non-residential destinations weighted and unweighted by LUM (DLUM 3b/9b). Associations were positive only in Melbourne and the US sites, suggesting the compensatory processes balancing the amounts of physical activity accrued during school and non-school periods in these locations may differ from those of other locations. This is a topic that would require further investigation in future studies.

### 6.6. Strengths and limitations

The development and validation of novel GIS-based DLUM indicators for international research on adolescents’ physical activity using comparable data from geographically and culturally diverse locations were major study strengths. Another study strength pertains to using diverse physical activity outcomes representing different domains, contexts and measurement methods (e.g., active transport to/from school; active transport to/from non-school destinations; accelerometer-based MVPA during non-school periods), which enabled a more fine-grained assessment of the construct validity of the various DLUM indicators. Study limitations were mainly related to differences in availability and comparability of spatial data across the study sites. For example, adolescents commonly participate in leisure-time physical activity, so accessibility to diverse types of recreation facilities, beyond parks, could be important. However, NEWS-Y inquiries about distance to 12 types of recreation facilities, in addition to large and small parks. However, GIS data on those facilities were not widely available across sites. The DLUM indicators were based on either mainly two-dimensional land area or parcel count data, while it would have been optimal to have floor area and destination count (e.g., number of food outlets) data to capture the three-dimensional nature of destination accessibility, especially in higher walkable neighbourhoods or cities with a high prevalence of mixed-use high-rise buildings, such as Hong Kong.

Other limitations included the lack of data on non-school active transport or parent-perceived destination accessibility in several study sites; the lack of data on parent-perceived proximity of entertainment destinations, which was a type of land use included in the DLUM indicators; having only one non-high-income (Curitiba, BRA) and ultra-dense study site (Hong Kong, CHN), raising concerns about the wider generalisability of the findings.

## 7. Conclusions and recommendations

We examined the cross-site comparability and utility of several established as well as novel GIS-based indicators of destination accessibility in an international study of adolescents’ physical activity conducted in 12 cities/regions varying in residential density, rate of motor vehicles per capita, prevalence of cycling as a mode of transport, and cultural context. We assessed DLUM indicators gauging intensity, heterogeneity, plus new combined intensity and heterogeneity measures of land use and parcels. Substantial differences in variability and average values of all 12 DLUM indicators were observed across cities/regions. Importantly, the ranking of the cities/regions on destination accessibility depended on the type of indicator. While Baltimore and Seattle (USA) performed poorly and Melbourne (Australia) performed relatively well on all indicators, the performance of other cities ranged from poor to excellent depending on the indicator used. DLUM indicators gauging heterogeneity and combined intensity and heterogeneity were only weakly related to measures of density and connectivity, indicating DLUM indicators represent a relatively independent component of neighbourhood walkability.

DLUM indicators based on the ratio of non-residential land area/parcels to residential areas/units (DLUM 1, 2, 7 and 8) and net density of non-residential parcels (DLUM 4 and 10) were not positively related to parental perceptions of destination accessibility across all study sites. Also, they showed negative (DLUM 1 and 7) or mixed associations with adolescents’ physical activity (DLUM 2, 4, 8 and 10) and, hence, are not appropriate indicators of destination accessibility for international research. DLUM indicators of heterogeneity (DLUM 5 and 6), especially those based on parcel counts (DLUM 6), were consistently positively related to parental perceptions of destination accessibility and adolescents’ active transport. Thus, DLUM 5 and 6 are among the recommended indicators.

Gross densities of non-residential parcels unweighted and weighted for a parcel-count-based LUM index were, respectively, the best performing intensity and hybrid (intensity + heterogeneity) indicators with respect to parent-perceived destination accessibility, adolescents’ active transport, and accelerometer-based MVPA, as well as cross-site comparability. Importantly, they were the only DLUM indicators associated with accelerometer-based MVPA during non-school periods. Because gross density of non-residential parcels weighted by the parcel-count based LUM index (DLUM 9b) captures both intensity and heterogeneity of destination accessibility and showed more parsimonious patterns of associations than its unweighted counterpart, it may be the most appropriate *single* DLUM indicator for international studies of various domains of physical activity in adolescents.

For studies focusing on specific physical activity domains, it may be more appropriate to choose the DLUM indicators most strongly associated with the domain of interest. For example, the parcel-count-based LUM index (DLUM 6) was the DLUM indicator most consistently and strongly associated with adolescents’ active transport, making it the preferred option for this particular physical activity domain. The area-based LUM index (DLUM 5) was the only indicator consistently positively related to total accelerometer-based MVPA and, thus, may be used alone or in conjunction with gross density of non-residential destinations (e.g., DLUM 3b/9b) to explain overall physical activity. Finally, to explain adolescents’ participation in sports and other leisure-time structured or unstructured activities, it may be more appropriate to employ spatial indicators of density of sporting and other physical activity facilities rather than overall measures of non-residential land use or destinations. Given that this is the first study to examine the construct validity of a range of newly developed DLUM indicators for international comparisons of destination accessibility and physical activity research, there is a need for future studies to assess their utility in other geographical contexts (e.g., low- and middle-income countries) and age groups, as well as with additional outcomes and land use categories.

## Supplementary Material

1

## Figures and Tables

**Table 1:** Socio-demographic characteristics, neighbourhood types and parental perceptions of proximity of destinations from home (*N* = 5052).

	All sites	Melbourne (AUS)	Ghent (BEL)	Curitiba (BRA)	Hong Kong (CHN)	Hradec Kralove (CZE)	Olomouc (CZE)	Odense (DNK)	Auckland (NZL)	Wellington (NZL)	Valencia (ESP)	Baltimore (USA)	Seattle (USA)
***N***	5052^#^	422	271	493	1291	155	183	207	449	188	465	485	443
***Child’s sex, %***													
Female	53.5	59.5	58.3	51.1	57.2	49.7	55.2	58.4	55.7	14.9	55.1	53.4	47.2
Male	46.5	40.5	41.7	48.9	42.8	50.3	44.8	41.6	44.3	85.1	44.9	46.6	52.8
***Child’s age (yrs)***													
M (SD)	14.4 (1.7)	14.9 (1.6)	13.4 (1.4)	14.1 (1.6)	14.3 (1.7)	14.3 (1.7)	13.7 (1.6)	13.0 (1.2)	14.7 (1.4)	14.5 (1.3)	15.6 (0.8)	14.2 (1.4)	14.0 (1.4)
***Highest education in household, %***													
Less than college degree	41.4	18.3	25.8	59.2	64.8	31.6	30.1	30.4	38.5	23.4	44.3	25.2	23.7
College degree or higher	49.9	34.8	72.7	40.8	35.2	32.3	15.8	66.2	48.8	70.2	55.7	73.8	76.1
Missing data	8.7	46.9	1.5	0.0	0.0	36.1	54.1	3.4	12.7	6.4	0.0	1.0	0.2
***Parental marital status, %***													
Not married or living with partner	16.1	12.1	14.8	28.0	10.3	14.8	12.0	15.9	20.5	17.0	21.3	19.4	12.2
Married or living with partner	76.2	43.3	84.8	72.0	89.7	48.4	36.6	83.6	71.9	78.2	78.7	79.6	87.6
Missing data	7.7	44.6	0.4	0.0	0.0	36.8	51.4	0.5	7.6	4.8	0.0	1.0	0.2
***Number of adults in household***													
M (SD)	2.4 (1.2)	2.2 (0.8)	2.1 (0.7)	2.4 (0.9)	2.5 (1.0)	2.2 (1.0)	2.1 (0.9)	2.0 (0.7)	3.2 (1.0)	3.3 (4.5)	2.3 (0.8)	2.2 (0.7)	2.2 (0.8)
Missing data, %	9.2	47.4	6.6	0.4	0.0	4.5	0.5	3.9	31.5	34.6	3.9	0.8	0.0
***Number of children in household***													
M (SD)	1.8 (0.9)	2.0 (0.9)	2.3 (1.3)	1.7 (1.0)	1.7 (0.7)	1.7 (0.7)	1.8 (0.7)	2.2 (1.1)	1.7 (0.9)	1.5 (0.8)	1.4 (0.6)	2.1 (1.2)	2.0 (1.0)
Missing data, %	8.9	47.2	3.7	0.4	0.0	4.5	0.5	2.9	30.8	34.0	3.9	0.8	0.0
***Motor vehicles in the household***													
M (SD)	1.4 (1.2)^#^	2.0 (1.0)	1.6 (0.9)	1.4 (1.0)	0.4 (0.70)	1.4 (0.8)	1.3 (1.0)	1.5 (1.0)	NA	NA	1.6 (0.9)	2.3 (1.0)	2.6 (1.0)
Missing data, %	8.6^#^	46.2	1.5	0.0	0.0	39.4	57.4	3.4	NA	NA	0.0	1.4	0.2
***Neighbourhood type, %***													
Low walkable / low SES	25.7	23.0	3.3	26.6	27.0	33.5	48.1	19.8	22.3	21.3	32.7	24.3	27.3
Low walkable / high SES	25.2	22.8	36.2	21.9	23.8	25.8	10.4	31.4	28.5	23.9	29.9	25.6	24.2
High walkable / low SES	26.1	31.4	46.5	30.2	26.5	16.8	7.6	25.6	29.6	28.2	13.8	24.7	23.5
High walkable / high SES	23.0	22.8	14.0	21.3	22.7	23.9	33.9	23.2	19.6	26.6	23.6	25.4	25.0
***Parental perceptions of proximity of destinations from home*** ^#^													
***Overall destinations***													
M (SD)	3.2 (0.9)^#^	3.1 (0.8)	3.3 (0.8)	3.0 (0.6)	3.4 (0.8)	3.2 (0.8)	3.0 (0.8)	3.1 (0.9)	NA	NA	4.1 (0.5)	2.7 (0.9)	2.0.8 (0.8)
Missing data, %	9.5^#^	45.7	22.1	0.0	0.0	36.8	57.4	0.0	NA	NA	0.0	1.0	0.2
***Commercial/retail/office destinations***													
M (SD)	3.2 (1.1)^#^	2.7 (1.0)	3.3 (0.9)	3.1 (0.9)	3.5 (1.0)	2.9 (0.9)	2.6 (1.0)	2.9 (1.1)	NA	NA	4.3 (0.7)	2.6 (1.0)	2.6 (1.0)
Missing data, %	8.2^#^	44.5	7.4	0.0	0.0	34.8	53.6	0.0	NA	NA	0.0	0.6	0.0
***Institutional/civic destinations***													
M (SD)	2.9 (0.9)^#^	2.7 (0.8)	3.3 (0.8)	2.8 (0.8)	2.9 (0.8)	3.5 (0.9)	3.2 (1.1)	3.0 (0.9)	NA	NA	3.7 (0.7)	2.5 (0.9)	2.5 (0.8)
Missing data, %	8.1^#^	44.1	5.9	0.0	0.0	35.5	51.4	0.0	NA	NA	0.0	0.8	0.2
***Food-related destinations***													
M (SD)	3.5 (1.0)^#^	3.3 (0.9)	3.5 (0.9)	3.1 (0.8)	3.7 (0.9)	3.4 (1.0)	3.3 (0.9)	3.2 (1.0)	NA	NA	4.4 (0.5)	2.9 (1.0)	2.9 (1.0)
Missing data, %	8.7^#^	44.5	14.8	0.0	0.0	34.2	53.6	0.0	NA	NA	0.0	0.8	0.0
***Parks***													
M (SD)	3.3 (1.2)^#^	3.8 (1.0)	3.1 (1.3)	2.7 (1.0)	3.4 (1.1)	3.0 (1.1)	2.3 (1.2)	3.3 (1.3)	NA	NA	4.0 (0.8)	2.9 (1.2)	3.1 (1.1)
Missing data, %	7.9^#^	44.5	3.7	0.0	0.0	34.8	51.9	0.0	NA	NA	0.0	0.4	0.0

Notes. AUS = Australia; BEL = Belgium; BRA = Brazil; CHN = China; CZE = Czech Republic; DNK = Denmark; NZL = New Zealand; ESP = Spain; USA = United States of America; SES =Socio-economic-status; M = mean; SD = standard deviation. NA = not applicable as data not collected.

# total sample size is 4415 for parental perceptions of destinations from home and motor vehicles in the household because New Zealand did not collect such data. The total sample size was 5106. However, 54 participants (1.1 %) did not have complete GIS data and were omitted, giving an analytical sample of 5052.

**Table 2:** Descriptive statistics of physical activity (PA) measures.

	All sites	Melbourne (AUS)	Ghent (BEL)	Curitiba (BRA)	Hong Kong (CHN)	Hradec Kralove (CZE)	Olomouc (CZE)	Odense (DNK)	Auckland (NZL)	Wellington (NZL)	Valencia (ESP)	Baltimore (USA)	Seattle (USA)
**Adolescent self-reported PA measures**													
***N***	5052^#^	422	271	493	1291	155	183	207	449	188	465	485	443
***Total non-school PA: Average number of days per week with at least 60 min/day of PA (excluding PE & gym classes at school)***													
M (SD)	2.8 (2.0)	3.4 (1.8)	2.9 (1.8)	1.7 (1.9)	2.0 (1.7)	3.2 (1.8)	3.3 (1.7)	4.0 (1.8)	3.5 (1.9)	4.0 (1.8)	2.5 (1.9)	3.6 (2.1)	3.7 (2.1)
Missing data, %	2.1	1.2	20.3	0.0	0.0	0.0	0.0	4.8	6.2	2.7	0.0	0.2	0.2
***Walking to/from school (weekly trips)***													
M (SD)	4.1 (4.4)	2.4 (2.3)	2.2 (3.8)	5.1 (4.6)	4.5 (4.5)	6.7 (4.2)	6.9 (4.2)	1.8 (3.3)	4.6 (4.3)	3.4 (3.8)	8.2 (3.4)	1.6 (2.9)	2.0 (3.3)
Missing data	4.3	2.4	12.2	0.2	0.0	29.0	30.0	4.8	6.0	2.7	0.0	3.9	5.2
***Regular walking to/from school (5 weekly trips) %***													
Yes	41.5	33.4	19.9	54.4	46.1	48.4	53.6	15.5	45.4	36.2	83.9	16.1	20.5
Missing data, %	4.3	2.4	12.2	0.2	0.0	29.0	30.0	4.8	6.0	2.7	0.0	3.9	5.2
***Active transport to/from school (weekly trips)***													
M (SD)	4.9 (4.6)	2.9 (3.0)	7.5 (4.7)	5.3 (4.7)	4.6 (4.6)	7.3 (4.1)	7.0 (4.2)	9.1 (3.0)	4.8 (4.3)	4.3 (4.1)	8.7 (3.1)	1.7 (3.0)	2.3 (3.5)
Missing data	4.1	2.4	9.2	0.2	0.0	29.0	30.0	4.8	6.0	2.7	0.0	3.9	5.2
***Regular active transport to/from school (5 weekly trips)***													
Yes	49.1	36.5	66.4	56.0	48.0	51.6	55.7	85.5	47.9	45.7	88.2	16.5	22.8
Missing data, %	4.1	2.4	9.2	0.2	0.0	29.0	30.0	4.8	6.0	2.7	0.0	3.9	5.2
***Non-school active transport: Weekly frequency of walking or cycling to/from destinations***													
M (SD)	5.0 (4.2)^#^	6.1 (4.1)	5.0 (3.5)	NA	6.3 (4.5)	5.6 (4.6)	5.6 (4.3)	4.9 (3.3)	3.5 (3.3)	4.9 (3.3)	NA	3.7 (4.2)	3.2 (3.3)
Missing data, %	1.8^#^	0.7	9.2	NA	0.0	0.0	0.0	4.8	6.2	2.7	NA	0.2	0.2
**Accelerometer-based measures**													
***N***	3481	371	206	419	549	49	56	124	337	154	373	438	405
***Average daily minutes of MVPA***													
M (SD)	42.7 (21.8)	47.4 (22.1)	38.4 (16.5)	37.3 (23.2)	36.4 (18.0)	55.9 (19.8)	60.0 (25.6)	45.0 (19.2)	52.3 (23.6)	60.9 (23.4)	46.2 (20.2)	37.2 (19.6)	37.8 (19.0)
***Average daily minutes of MVPA during non-school periods***													
M (SD)	29.9 (17.6)	29.7 (16.4)	23.5 (12.7)	28.3 (19.6)	24.5 (13.7)	43.9 (16.7)	46.9 (23.2)	25.8 (14.5)	35.4 (18.7)	42.0 (20.0)	36.2 (18.3)	27.5 (16.2)	27.1 (15.3)
***Total valid days of accelerometer wear***													
M (SD)	6.9 (1.4)	6.9 (1.2)	7.2 (1.5)	6.7 (1.1)	7.0 (1.8)	6.5 (1.2)	6.2 (1.2)	6.2 (1.7)	7.3 (0.9)	7.1 (1.0)	6.3 (1.3)	7.2 (1.3)	7.3 (1.5)
***Average wear time minutes per valid day***													
M (SD)	803.8 (77.2)	786.0 (74.2)	797.3 (71.7)	806.8 (85.1)	776.3 (79.0)	804.9 (92.9)	794.6 (79.9)	772.7 (71.4)	808.9 (60.6)	807.9 (61.0)	860.3 (63.3)	813.6 (76.6)	799.3 (70.8)
***Average wear time minutes during non-school periods per valid day***													
M (SD)	528.6 (99.3)	497.4 (86.8)	488.1 (87.1)	601.0 (108.4)	452.0 (93.1)	572.2 (89.5)	532.7 (95.6)	470.0 (90.2)	517.4 (67.7)	521.6 (65.9)	590.0 (68.2)	553.0 (86.2)	547.3 (82.0)

*Notes*. AUS = Australia; BEL = Belgium; BRA = Brazil; CHN = China; CZE = Czech Republic; DNK = Denmark; NZL = New Zealand; ESP = Spain; USA = United States of America; M = mean; SD = standard deviation; PE = physical education. NA = not applicable as data not collected; MVPA = moderate-to-vigorous physical activity.

# total sample size is 4094 for weekly frequency of walking or cycling to/from destinations because Brazil and Spain did not collect such data.

**Table 3:** GIS-based destination and land-use mix indicators (DLUM).

Indicators		Operationalisation	Remarks
DLUM 1	Ratio of land area of 5 non-residential land uses to residential land area	Sum of the land areas of 5 land uses in a participant buffer, divided by the residential area of the buffer	Level of potential exposure to 1–5 non-residential land uses. However, heterogeneity of land uses is not explicitly captured by this indicator.
DLUM 2	Ratio of parcel counts of 5 non-residential land uses to dwelling unit count	Sum of # of parcels for 5 land uses in a participant buffer, divided by the number of dwelling units in the buffer	Level of supply of 1–5 destination types. Higher values denoting higher supply of destinations. However, heterogeneity of destination types is not explicitly captured by this indicator.
DLUM 3a	Parcel counts of 5 non-residential land uses	Sum of # of parcels for 5 land uses in a participant buffer	Intensity/density of 1–5 destinations in participant buffers. However, heterogeneity of destination types is not explicitly captured by this indicator.
DLUM 3b	Gross density of parcels of 5 non-residential land uses (parcels/km^2^)	Sum of # of parcels for 5 land uses in a participant buffer, divided by the buffer area	As above.
DLUM 4	Net density of parcels of 5 non-residential land uses (parcels/km^2^)	Sum of # of parcels for 5 land uses in a participant buffer, divided by the sum of the areas of 5 land uses	Average number of 5-land-use parcels per 5-land-use area unit. Higher values denoting higher overall destination density in non-residential land. However, heterogeneity of destination types is not explicitly captured by this indicator.
DLUM 5	Area-based non-residential land use mix index (5 land uses)	Entropy score based on information on proportion of land (p.land) per type of land use (k) and number of non-residential land uses (N). $-\frac{\sum_{k}p{\text{.land}}_{k}\times \text{ln}\left(p{\text{.land}}_{k}\right)}{\text{ln}(N)}$	Captures heterogeneity of non-residential land use area but not density of non-residential destinations or heterogeneity of non-residential destination units.
DLUM 6	Parcel-count-based non-residential land use mix index (5 land uses)	Entropy score based on information on proportion of parcels (p.parcel.count) per type of land use (k) and number of non-residential land uses (N). $-\frac{\sum_{k}\text{p.parcel}{\text{.count}}_{k}\times \text{ln}\left(\text{p.parcel}{\text{.count}}_{k}\right)}{\text{ln}(N)}$	Captures heterogeneity but not density of non-residential destinations.
DLUM 7	Ratio of land area of 5 non-residential land uses to residential land area weighted by area-based land use mix index	DLUM 1 multiplied by (1 + DLUM 5)	Captures both potential exposure to and heterogeneity of 5 non-residential land uses.
DLUM 8	Ratio of parcel counts of 5 non-residential land uses to dwelling unit count weighted by parcel-count-based land use mix index	DLUM 2 multiplied by (1 + DLUM 6)	Captures both level of supply and heterogeneity of 5 non-residential destinations.
DLUM 9a	Parcel counts of 5 non-residential land uses weighted by parcel-count-based land use mix index	DLUM 3a multiplied by (1 + DLUM 6)	Captures both intensity/density and heterogeneity of non-residential destinations.
DLUM 9b	Gross density of parcels of 5 non-residential land uses weighted by parcel-count-based land use mix index (parcels/km^2^)	DLUM 3b multiplied by (1 + DLUM 6)	As above.
DLUM 10	Net density of parcels of 5 non-residential land uses weighted by parcel-count-based land use mix index (parcels/km^2^)	DLUM 4 multiplied by (1 + DLUM 6)	Captures both density and heterogeneity of destinations within non-residential land.

*Notes*. 5 land uses are: commercial/retail/office; institutional/civic; food-related; entertainment; parks.

**Table 4:** Ranking of study sites by destination and land-use mix (DLUM) indicators (unadjusted for residential and intersection density).

Indicator	Melbourne (AUS)	Ghent (BEL)	Curitiba (BRA)	Hong Kong (CHN)	Hradec Kralove (CZE)	Olomouc (CZE)	Odense (DNK)	Auckland (NZL)	Wellington (NZL)	Valencia (ESP)	Baltimore (USA)	Seattle (USA)
DLUM 1: Ratio of land area of 5 land uses to residential land area	6	9	4	**1**	7	11	**2**	5	**3**	8	10	12
DLUM 2: Ratio of parcel counts of 5 land uses to dwelling unit count	**3**	6	**1**	11	7	4	**2**	5	8	12	9	10
DLUM 3a: Parcel counts of 5 land uses	4	5	**1**	8	7	6	**2**	9	10	**3**	11	12
DLUM 3b: Gross density of parcels of 5 land uses (parcels/km^2^)	**3**	5	**1**	7	10	6	**2**	8	9	4	11	12
DLUM 4: Net density of parcels of 5 land uses (parcels/km^2^)	7	5	**2**	**3**	4	**1**	8	9	11	6	10	12
DLUM 5: Area-based land use mix index (5 land uses)	**3**	4	7	**2**	8	**1**	11	5	9	6	12	10
DLUM 6: Parcel-count-based land use mix index (5 land uses)	4	**1**	11	5	**3**	8	9	6	**2**	7	12	10
DLUM 7: Ratio of land area of 5 land uses to residential land area weighted by area-based land use mix index	5	8	4	**1**	7	10	**2**	6	**3**	9	11	12
DLUM 8: Ratio of parcel counts of 5 land uses to dwelling unit count weighted by parcel-count-based land use mix index	**3**	6	**1**	11	7	4	**2**	5	8	12	9	10
DLUM 9a: Parcel counts of 5 land uses weighted by parcel-count-based land use mix index	**2**	5	**1**	**3**	10	4	7	6	8	9	11	12
DLUM 9b: Gross density of parcels of 5 land uses weighted by parcel-count-based land use mix index (parcels/km^2^)	**2**	6	**1**	**3**	10	7	5	4	8	9	11	12
DLUM 10: Net density of parcels of 5 land uses weighted by parcel-count-based land use mix index (parcels/km^2^)	7	4	**2**	**3**	5	**1**	8	9	11	6	10	12
Mean ranking	**4.1**	5.3	**3.0**	**4.8**	7.1	5.3	5.0	6.4	7.5	7.6	10.6	11.3
Standard deviation of ranking	1.8	2.0	3.1	3.6	2.3	3.4	3.4	1.8	3.1	2.8	1.0	1.0

*Notes*. AUS = Australia; BEL = Belgium; BRA = Brazil; CHN = China; CZE = Czech Republic; DNK = Denmark; NZL = New Zealand; ESP = Spain; USA = United States of America. In bold: study sites ranked 1st, 2nd and 3rd.

**Table 5:** Associations of destination and land-use mix (DLUM) indicators with adolescents’ physical activity (PA).

Indicators	Total non-school PA^1^	Regular walking to/from school (Y/N)^2^	Regular active transport to/from school (Y/N)^2^	Non-school active transport (weekly frequency)	Total MVPA (average min/day)	MVPA during non-school periods (average min/day)
	*e*^*b*^ (95 % CI)	OR (95 % CI)	OR (95 % CI)	e^*b*^ (95 % CI)	e^*b*^ (95 % CI)	e^*b*^ (95 % CI)
DLUM 1: Ratio of land area of 5 land uses to residential land area	1.000 (0.999, 1.000)	0.998* (0.996, 0.999)	0.997** (0.994, 0.998)	0.9998 (0.9993, 1.0002)	0.9995* (0.9992, 0.9999)	1.000 (0.999, 1.000)
DLUM 2: Ratio of parcel counts of 5 land uses to 100 dwelling unit count	1.004** (1.001, 1.006)	Moderated by TNSPA (Table 6)	Moderated by TNSPA (Table 6)	Moderated by site (Table S6)	1.000 (0.997, 1.002)	1.0000 (0.9998, 1.0002)
DLUM 3a: Parcel counts of 5 land uses	1.000 (0.999, 1.000)	1.001** (1.0003, 1.002)	Moderated by TNSPA (Table 6)	1.0004*** (1.0002, 1.0006)	Moderated by site (Table S7)	1.000 (0.999, 1.000)
DLUM 3b: Gross density of parcels of 5 land uses (parcels/km^2^)	1.000 (0.999, 1.000)	Moderated by TNSPA (Table 6)	Moderated by TNSPA (Table 6)	Curvilinear*** (Fig. 13A)	Moderated by site (Table S7)	1.001* (1.000, 1.002)
DLUM 4: Net density of parcels of 5 land uses (parcels/km^2^)	Curvilinear* (Fig. 13C)	1.000 (0.999, 1.000)	1.000 (0.999, 1.000)	Moderated by site (Table S6)	1.000 (0.999, 1.000)	1.000 (0.999, 1.000)
DLUM 5: Area-based land use mix index (5 land uses)	0.945 (0.886, 1.009)	2.013*** (1.386, 2.922)	Moderated by DTS (Table S8)	1.291*** (1.175, 1.418)	Curvilinear* (Fig. 13B)	1.001 (0.995, 1.009)
DLUM 6: Parcel-count-based land use mix index (5 land uses)	0.880*** (0.806, 0.961)	2.424*** (1.438, 4.084)	2.452*** (1.466, 4.099)	1.373*** (1.214, 1.552)	1.032 (0.938, 1.134)	1.002 (0.995, 1.009)
DLUM 7: Ratio of land area of 5 land uses to residential land area weighted by area-based land use mix index	1.000 (0.999, 1.000)	0.998* (0.996, 0.999)	0.997** (0.994, 0.999)	0.9998 (0.9994, 1.0003)	0.9996* (0.9992, 0.9999)	1.000 (0.999, 1.000)
DLUM 8: Ratio of parcel counts of 5 land uses to 100 dwelling unit count weighted by parcel-count-based land use mix index	1.002** (1.001, 1.004)	0.996 (0.987, 1.005)	1.000 (0.991, 1.009)	Moderated by site (Table S6)	1.000 (0.998, 1.002)	1.000 (0.999, 1.000)
DLUM 9a: Parcel counts of 5 land uses weighted by parcel-count-based land use mix index	1.000 (0.999, 1.000)	1.001** (1.0002, 1.001)	Moderated by TNSPA (Table 6)	1.0002*** (1.0001, 1.0004)	Moderated by site (Table S7)	1.000 (0.999, 1.000)
DLUM 9b: Gross density of parcels of 5 land uses weighted by parcel-count-based land use mix index (parcels/km^2^)	1.000 (0.999, 1.000)	1.0005* (1.0000, 1.0009)	Moderated by TNSPA (Table 6)	1.0003*** (1.0002, 1.0005)	Moderated by site (Table S7)	1.0007* (1.000, 1.001)
DLUM 10: Net density of parcels of 5 land uses weighted by parcel-count-based land use mix index (parcels/km^2^)	Curvilinear* (Fig. 13D)	1.000 (0.999, 1.000)	1.000 (0.999, 1.000)	Moderated by site (Table S6)	1.000 (0.999, 1.000)	1.000 (0.999, 1.000)

*Notes*. TNSPA = total non-school physical activity (days per week of meeting the PA guidelines via non-school PA); MVPA = moderate-to-vigorous PA; DTS = distance to school (from home); OR = odds ratio; CI = confidence intervals; e^*b*^ = exponentiated value of regression coefficient.

1 Average number of days per week with at least 60 min/day of PA (excluding PE & gym classes at school).

2 5+ trips per week.

* *p* < .05;

** *p* < .01;

*** *p* < .001.

All models included cities, neighbourhood types, adolescent sex and age, parental marital status, persons in the household and highest education in the household as covariates and accounted for neighbourhood and school level clustering effects arising from the participant recruitment strategies. Models of MVPA were also adjusted for valid days and time of accelerometer wear. Tables S6 and S7 are in Supplementary Material.

**Table 6:** Associations of destination and land-use mix (DLUM) indicators with adolescents’ physical activity (PA) moderated by self-reported non-school PA.

DLUM indicator	Self-reported non-school PA (60+ minutes a day)	Regular walking to/from school (Y/N)^1^		Regular active transport to/from school (Y/N)^1^	
		OR	95 % CI	OR	95 % CI
DLUM 2: Ratio of parcel counts of 5 land uses to 100 dwelling unit count	0 days	0.997	(0.972, 1.024)	1.000	(0.976, 1.024)
	2 days	0.979*	(0.962, 0.997)	0.984*	(0.968, 0.999)
	4 days	0.977*	(0.959, 0.996)	0.983*	(0.966, 0.999)
	6 days	0.980	(0.963, 0.998)	0.977	(0.982, 1.013)
DLUM 3a: Parcel counts of 5 land uses	0 days	–	–	1.001*	(1.000, 1.002)
	2 days	–	–	1.000	(0.999, 1.001)
	4 days	–	–	1.000	(0.999, 1.001)
	6 days	–	–	1.001	(0.999, 1.002)
DLUM 3b: Gross density of parcels of 5 land uses (parcels/km^2^)	0 days	1.001*	(1.000, 1.002)	1.001*	(1.000, 1.002)
	2 days	1.000	(0.999, 1.001)	0.999	(0.998, 1.001)
	4 days	1.000	(0.999, 1.001)	1.000	(0.999, 1.001)
	6 days	1.001*	(1.001, 1.002)	1.001	(0.999, 1.002)
DLUM 9a: Parcel counts of 5 land uses weighted by parcel-count-based land use mix index	0 days	–	–	1.001*	(1.000, 1.002)
	2 days	–	–	1.000	(0.999, 1.001)
	4 days	–	–	1.000	(0.999, 1.001)
	6 days	–	–	1.000	(0.999, 1.001)
DLUM 9b: Gross density of parcels of 5 land uses weighted by parcel-count-based land use mix index (parcels/km^2^)	0 days	–	–	1.001*	(1.000, 1.002)
	2 days	–	–	0.999	(0.998, 1.001)
	4 days	–	–	1.000	(0.999, 1.001)
	6 days	–	–	1.000	(0.999, 1.001)

*Notes*. All models included cities, neighbourhood types, adolescent sex and age, parental marital status, persons in the household and highest education in the household as covariates and accounted for neighbourhood and school level clustering effects arising from the participant recruitment strategies. Models used binomial variance and logit link functions. OR = odds ratios; CI = confidence intervals (in brackets). – = not applicable.

* *p* < .05;

1 5+ trips per week.

## Data Availability

Data sharing is not currently available because multiple manuscripts examining IPEN Adolescent data are in progress. IPEN study methods, surveys, protocols, and publications are available on the IPEN website: https://ipenproject.org/the-international-physical-activity-and-the-environment-network-about-us/ipen-studies/ipen-adolescent-study/.

## Appendix A. Supplementary data

Supplementary data to this article can be found online at https://doi.org/10.1016/j.cities.2025.106044.
