# Supplementary material for: Evaluating the performance of spatial indicators of destination accessibility for physical activity research: a comparative international analysis
Source: Cities. Author manuscript; Available in PMC 2026 May 29. (PMC13218646; doi:10.1016/j.cities.2025.106044)
Supplement: 1 [file NIHMS2174968-supplement-1.docx]

**Supplementary Material**

**Content**

**Detailed Methods**

**Table S1.** Descriptive statistics of GIS-based destination and land-use mix (DLUM) indicators and other neighbourhood characteristics

**Table S2.** Net residential and street intersection densities as mediators of between-site differences in destination and land-use mix indicators

**Table S3.** Associations between GIS-based destination and land-use mix (DLUM) indicators and parental perceived proximity of (overall) destinations from home

**Table S4.** Associations of ratio of land area of 5 land uses to residential land area indicators with parental perceived proximity of destinations from home by study site

**Table S5.** Associations of ratio of area-based and parcel-count-based land use mix indices with parental perceived proximity of destinations from home by study site

**Table S6.** Associations of GIS-based destination and land-use mix (DLUM) indicators with adolescents’ non-school active transport (weekly frequency) by study site (only significant moderation effects reported)

**Table S7.** Associations of GIS-based destination and land-use mix (DLUM) indicators with adolescents’ accelerometer-based total moderate-to-vigorous physical activity (MVPA) (average min/day) by study site (only significant moderation effects reported)

**Table S8.** Associations between area-based land-use mix index (DLUM 5) and regular active transport to/from school by distance from home to school

**Table S9.** Associations of gross parcel densities of specific land uses (parcels/km^2^) with adolescents’ physical activity (PA)

**Table S10.** Associations of gross density of food-related parcels (parcels/km^2^) with adolescents’ active transport to/from school outcomes by distance from home to school

**Table S11.** Associations of gross density of parks (parks/km^2^) with adolescents’ non-school active transport (weekly frequency) by study site

**Figure S1.** Overview of study sites, design and aims

**Figure S2.** Correlation matrix of destination and land-use mix indicators (Pearson’s correlation coefficients)

**Figure S3.** Associations of net density of parcels of 5 land uses indicators (parcels/km^2^) unweighted for parcel-count-based land use mix index (DLUM 4) with parental perceived proximity of destinations from home by study site

**Figure S4.** Associations of net density of parcels of 5 land uses indicators (parcels/km^2^) weighted for parcel-count-based land use mix index (DLUM 10) with parental perceived proximity of destinations from home by study site

**Figure S5.** Associations of GIS-based gross densities of land use parcels with parental perceived proximities of corresponding destinations

**Figure S6.** Associations of GIS-based gross densities of land use parcels with adolescents’ non-school active transport (weekly frequency)

**Detailed Methods**

We utilised comparable GIS, survey and device-based physical activity data collected in 12 cities/regions across nine countries that participated in the IPEN Adolescent study. The selected study sites varied substantially in net dwelling density, motor vehicles per capita, prevalence of cycling to/from school, and cultural regions (Figure S1), enabling an evaluation of spatial indicators of destination accessibility in various geographical and cultural contexts. The following sections detail the study design of IPEN Adolescent and the measures used in the present study, including neighbourhood GIS-based spatial indicators of destination accessibility [here named destination and land-use mix (DLUM) indicators] and other attributes, parental perceptions of destination accessibility, and adolescents’ physical activity (Figure S1).

***Study design and sample***

IPEN Adolescent is an observational, cross-sectional study conducted in 15 countries across six continents (Cain et al., 2021). Nine of these countries, including 12 cities/regions and a total of 5052 participants, had access to comparable GIS data. These were Australia (AUS, Melbourne), Belgium (BEL, Ghent), Brazil (BRA, Curitiba), China (CHN, Hong Kong), Czech Republic (CZE, Hradec Kralove and Olomouc), Denmark (DNK, Odense), New Zealand (NZL, Auckland and Wellington), Spain (ESP, Valencia) and the United States of America (USA, Baltimore and Seattle regions) (Figure S1; Table 1; Table S1).

A stratified two-stage sampling strategy was employed to recruit adolescents (aged 11-19 years) from study sites. All sites, except Auckland and Wellington (NZL), also recruited one of the adolescents’ primary caregivers who in a separate survey self-reported proximity of multiple types of destinations from home (n = 4415). The sampling strategy first entailed the selection of census/administrative unit areas stratified into quadrants of higher- vs. lower-walkability (using a composite GIS-based measure of residential density, street intersection density, and LUM) by higher- vs. lower-socioeconomic status (SES, based on country-specific demographic data, primarily area-level income or educational attainment) (Figure S1). Some countries then recruited participants via schools and others recruited directly based on home addresses in the pre-selected areas. Purposive recruitment was done to ensure variability and balance of built environments, SES, and participants’ characteristics (e.g., sex and age range) within cities. The IPEN Adolescent study was approved by the Institutional Review Boards at San Diego State University and the University of California San Diego. Study protocols and data collection in each country were approved by their Institution’s Ethics Committees. Details on the Ethics Boards and approval numbers are provided in Cain et al. (2021). Written informed consent/assent was obtained from all participants. Detailed information on the study design, sites, and recruitment procedures was previously reported (Cain et al., 2021).

While all 9 countries had relatively complete self-report data on adolescents’ active travel to/from school and outside of school physical education classes, only a subsample of adolescents had valid accelerometer-based physical activity data (n = 3481) (Table 2). Brazil and Spain did not collect adolescents’ self-report data on active transport to non-school destinations (Table 2).

***Measures***

*GIS-based neighbourhood characteristics*

GIS staff in each country followed a predeveloped common set of 15 GIS templates based on previous work (available at https://ipenproject.org/) that operationalized constructs, provided greater specificity to common concepts, offered clearer definitions, and gave guidance on creating GIS variables and specific procedures. Each template also included a set of questions to evaluate the degree of match to the definitions and to the required approach for the current study. Two experts reviewed each country's responses to these questions and requested clarifications or refinements of variables, as appropriate. This overall approach ensured a common set of comparable GIS variables and allowed for the documentation of any deviations.

Participants’ residential addresses were geocoded and road network-based buffers of prespecified sizes were created in GIS around each participant’s home (Frank et al., 2017). The present analyses used only 1-km-street network sausage buffer, with 25 meter offset, because they capture parcels adjacent to the road and have shown more consistent associations with physical activity (Frank et al., 2017). For each buffer, a wide range of GIS-based environmental attributes were computed. This study utilised the following subset: net residential density (dwellings/km2 of residential land); street intersection density (intersections/km2 of total buffer area); gross parcel densities of each of five groups of land uses (parcels/km2 of total buffer area), i.e., (1) commercial/retail/office, (2) institutional/civic, (3) food-related, (4) entertainment, and (5) parks; and 12 destination and land-use mix (DLUM) indicators. The DLUM indicators are shown in Table 3 and consisted of five indicators of overall availability/intensity of the five groups of non-residential land uses or destination types (DLUM 1 to DLUM 4), two indicators of heterogeneity (entropy) of land uses or destination types (DLUM 5 and 6), and five indicators representing hybrid measures of availability/intensity and heterogeneity of the five groups of non-residential land uses or destinations (DLUM 7 to DLUM 10).

*Parent-perceived proximity of destinations from home*

For one evaluation of construct validity, we used items from the Neighborhood Environment Walkability Scale for Youth (NEWS-Y) (Cerin et al., 2019) to assess parental perceived proximity of 15 destinations from home, representing perceived destination accessibility. Caregivers reported the walking time to reach the nearest destination (of a specific type) from home. Responses ranged from 1 to 5-minute walking distance (assigned a score of 5, i.e., high accessibility) to 30-minutes or more walking distance (assigned a score of 1, i.e., low accessibility). For the purpose of the present study, five composite measures were computed to mirror the self-report counterpart measures of GIS-based measures of gross densities of commercial/office, institutional/civic, food-related, and park parcels. First, an overall score of the average responses across all 15 types of destinations, named “proximity of destinations from home,” represented the self-report counterpart measure of the GIS-based destination and land-use mix indicators. The other four measures represented average scores for perceived proximity of parks (2 items: small and large public parks), commercial/office (4 items: bank/credit union, pharmacy/drug store, laundry or dry cleaners, clothing store), institutional/civic (4 items: library, post office, any school, your child school), and food-related destinations (5 items: supermarket, convenience/small grocery, coffee place, fast food restaurant, non-fast food restaurant). No NEWS-Y items captured entertainment destinations.

*Adolescent’s physical activity*

This study examined several self-report and accelerometer-based measures of adolescents’ physical activity, used as a second set of construct validity measures. Self-report measures included total non-school physical activity, active transport to/from school, walking to/from school, and non-school active transport. Accelerometer-based measures of physical activity were average daily minutes of moderate-to-vigorous physical activity (MVPA) and average daily minutes of MVPA during non-school periods.

Self-reported total non-school physical activity was measured using two items asking adolescents to report the number of days per week they were physically active for at least 60 minutes in the last week and during a typical week, excluding physical education or gym classes at school (Prochaska et al., 2001). These items had acceptable test-retest reliability (ICC>0.70) and criterion validity compared to accelerometers (Cerin et al., 2014; Prochaska et al., 2001; Ridgers et al., 2012). Responses on the two items were averaged to represent days of meeting physical activity guidelines (60+ min/day) (range: 0 – 7). Active transport to/from school was defined as the number of walking plus cycling trips to and from school in an average school week (range: 0 – 20). Walking to/from school was operationalised as the weekly number of walking trips to and from school (range: 0 – 10). Adolescents were also asked how long it would take them to walk to school, as a proxy for distance to school. These items were adapted from the U.S. Centers for Disease Control and Prevention’s Kids-Walk-to-School program (National Center for Chronic Disease Prevention and Health Promotion, 2000) and showed good test-retest reliability in various populations (Cerin et al., 2014; Jo et al., 2024). Regular walking and active transport to/from school were defined as accumulating 5+ trips per week (i.e., at least one trip per school day). Non-school active transport was defined as the total frequency of walking or cycling to/from six destinations (e.g., indoor or outdoor recreation places, stores or food outlets, social activities, transit stops) in a typical week. Responses were recorded on a 6-point frequency scale ranging from 0 (never) to 5 (4+ times/week) and recoded as follows: never = 0; once a month or less = 0.25; every other week = 0.50; once a week = 1; 2-3 times a week = 2.5; 4 or more times a week = 4 (range = 0 - 24). Test-retest reliability of these items was moderate to high (Cerin et al., 2014; Jo et al., 2024). All study sites collected data on all self-report measures of physical activity, except non-school active transport was not included in Curitiba (BRA) and Valencia (ESP).

Adolescents were asked to wear an ActiGraph accelerometer on a belt over the right hip for at least seven days during waking hours, excluding water activities. Various ActiGraph models were used (GT1M, GT3X, GT3X+ and 7164 models) across study sites. All data were collected with, or converted to, a 30-second epoch. Non-wear time was defined as ≥60 minutes of consecutive zero counts (Cain et al., 2018), and a valid wearing day consisted of at least 8 hours of wear time during waking hours of 6:00 AM to 12:00 PM. Only participants with at least 4 valid wearing days were included in the analyses. Evenson’s cut points for physical activity (≥ 2296 counts per minute) were used to process the vertical axis accelerometer data (Evenson et al., 2008) and obtain estimates of average daily minutes of MVPA during valid wearing time (a) over the entire monitoring period and (b) during non-school periods. Self-reported school start and end times were used in most countries to determine school days and in-school times. As these data were not available in the USA, the period from 8:15 AM to 2:15 PM was used as an estimate of the school day on weekdays. A more detailed description of the accelerometer-based physical activity measures can be found in Cain et al. (Cain et al., 2021).

*Socio-demographic characteristics*

Participants (adolescents and/or parents) self-reported socio-demographic characteristics, including adolescents’ sex and age, parental marital status, highest education in the household, household composition, and motor vehicles in the household.

***Data Analysis***

Descriptive statistics of all variables included in this study were computed for the whole sample and by study site. Patterns of missing data were examined and sets of 20 multiple imputed datasets were created using chained equations because more than 5% of cases (12.8% to 18.1%) had missing data on at least one of the variables included in regression models (van Buuren, 2018). Random intercept generalised additive mixed models (GAMMs) accounting for clustering at the administrative unit and school levels were used to: (aim 1) estimate between-site differences in DLUM indicators; (aim 2) establish the extent to which these differences were due to residential and intersection densities; (aims 3 and 4) estimate associations of DLUM indicators with parental perceived proximity of destinations from home and adolescents’ physical activity; and (aim 5) determine whether these associations were generalisable across study sites (Figures 1 and S1). GAMMs can accommodate outcomes with different distributions and model curvilinear associations of unknown form using semiparametric smooth terms (in this case, thin plate splines) (Wood, 2017).

To address aims 1 and 2, GIS-based DLUM indicators (i.e., DLUM indicators 1 to 10 in Table 3) and gross parcel densities of specific land uses were first regressed onto study site and confounders (sociodemographic variables and neighbourhood type) to obtain their confounder-adjusted marginal means and 95% CIs for each site. Inter-site variance in marginal means was then computed for each DLUM indicator. To examine the extent to which net residential and street intersection densities explained (i.e., mediated) inter-site differences in DLUM indicators (aim 2), net residential and street intersection densities were added to the previous GAMMs and the inter-site variance in DLUM indicators was re-computed. Net residential and street intersection densities were considered mediators of inter-site differences in DLUM indicators if they themselves differed by site and were significantly associated with DLUM indicators (Cerin & MacKinnon, 2009).

To address aim 3, DLUM indicators and gross parcel densities of specific land uses were regressed onto matching composite measures of parent-perceived proximity to destinations from home. These models were adjusted for study site, area-level SES, and sociodemographic variables. The heterogeneity of associations across sites was examined by adding appropriate interaction terms to the main effect GAMMs (aim 5). To achieve aim 4, each physical activity outcome was regressed onto each DLUM indicator and the set of gross parcel densities of land uses (and confounders/covariates). Separate models were estimated for each DLUM indicator and the set of gross parcel densities of land uses. Apart from being adjusted for study site, area-level SES and sociodemographic characteristics, these GAMMs were adjusted for net residential density and street intersection density because they are potential confounders (Cerin et al., 2020). GAMMs of accelerometer-based MVPA were also adjusted for accelerometer-wear variables (e.g., average wear time on a valid day). Self-reported distance to school was included as a moderator of the relations of the GIS measures with active transport to/from school and accelerometer-based MVPA outcomes in a separate set of GAMMs (Timperio et al., 2025). Additionally, self-reported total non-school physical activity was considered as a potential moderator of the associations of environmental variables with active transport and walking to/from school because regular engagement in non-school leisure physical activity and sports may limit the time available to actively commute to/from school. A separate set of GAMMs examined the heterogeneity of these associations across study sites (aim 5). Multicollinearity was assessed by computing the Variance Inflation Factor (VIF) for each variable included in the models.

**References**

Cain, K. L., Bonilla, E., Conway, T. L., Schipperijn, J., Geremia, C. M., Mignano, A., Kerr, J., & Sallis, J. F. (2018). Defining accelerometer nonwear time to maximize detection of sedentary time in youth. Pediatric Exercise Science, 30(2), 288–295. https://doi.org/10.1123/pes.2017-0132

Cain, K. L., Salmon, J., Conway, T. L., Cerin, E., Hinckson, E., Mitáš, J., Schipperijn, J., Frank, L. D., Anjana, R. M., Barnett, A., Dygrýn, J., Islam, M. Z., Molina-García, J., Moran, M., Wan Muda, W. A. M., Oyeyemi, A. L., Reis, R., Santos, M. P., Schmidt, T., Schofield, G. M., … Sallis, J. F. (2021). International Physical Activity and Built Environment Study of adolescents: IPEN Adolescent design, protocol and measures. BMJ Open, 11(1), e046636. https://doi.org/10.1136/bmjopen-2020-046636

Cerin, E., Barnett, A., Zhang, C. J. P., Lai, P. C., Sit, C. H. P., & Lee, R. S. Y. (2020). How urban densification shapes walking behaviours in older community dwellers: a cross-sectional analysis of potential pathways of influence. International Journal of Health Geographics, 19(1), 14. https://doi.org/10.1186/s12942-020-00210-8

Cerin, E., Conway, T. L., Barnett, A., Smith, M., Veitch, J., Cain, K. L., Salonna, F., Reis, R. S., Molina-García, J., Hinckson, E., Muda, W. A. M. W., Anjana, R. M., van Dyck, D., Oyeyemi, A. L., Timperio, A., Christiansen, L. B., Mitáš, J., Mota, J., Moran, M., Islam, M. Z., … Sallis, J. F. (2019). Development and validation of the neighborhood environment walkability scale for youth across six continents. International Journal of Behavioral Nutrition and Physical Activity, 16(1), 122. https://doi.org/10.1186/s12966-019-0890-6

Cerin, E., & Mackinnon, D. P. (2009). A commentary on current practice in mediating variable analyses in behavioural nutrition and physical activity. Public Health Nutrition, 12(8), 1182–1188. https://doi.org/10.1017/S1368980008003649

Cerin, E., Sit, C. H., Huang, Y. J., Barnett, A., Macfarlane, D. J., & Wong, S. S. (2014). Repeatability of self-report measures of physical activity, sedentary and travel behaviour in Hong Kong adolescents for the iHealt(H) and IPEN - Adolescent studies. BMC Pediatrics, 14, 142. https://doi.org/10.1186/1471-2431-14-142

Evenson, K. R., Catellier, D. J., Gill, K., Ondrak, K. S., & McMurray, R. G. (2008). Calibration of two objective measures of physical activity for children. Journal of Sports Sciences, 26(14), 1557–1565. https://doi.org/10.1080/02640410802334196

Frank, L. D., Fox, E. H., Ulmer, J. M., Chapman, J. E., Kershaw, S. E., Sallis, J. F., Conway, T. L., Cerin, E., Cain, K. L., Adams, M. A., Smith, G. R., Hinckson, E., Mavoa, S., Christiansen, L. B., Hino, A. A., Lopes, A. A., & Schipperijn, J. (2017). International comparison of observation-specific spatial buffers: maximizing the ability to estimate physical activity. International Journal of Health Geographics, 16(1), 4. https://doi.org/10.1186/s12942-017-0077-9

Joe, L., Carlson, J. L., & Sallis, J. F. (2023). Active Where? Individual item reliability: adolescent survey. [https://www.drjimsallis.com/active-where] (accessed 2024 Jul 20)

National Center for Chronic Disease Prevention and Health Promotion (U.S.) (CDC). (2000). KidsWalk-to-School: a guide to promote walking to school [Internet]. CDC. https://stacks.cdc.gov/view/cdc/11316 (accessed 2024 Jul 20)

Prochaska, J. J., Sallis, J. F., & Long, B. (2001). A physical activity screening measure for use with adolescents in primary care. Archives of Pediatrics & Adolescent Medicine, 155(5), 554–559. https://doi.org/10.1001/archpedi.155.5.554

Ridgers, N. D., Timperio, A., Crawford, D., & Salmon, J. (2012). Validity of a brief self-report instrument for assessing compliance with physical activity guidelines amongst adolescents. Journal of Science and Medicine in Sport, 15(2), 136–141. https://doi.org/10.1016/j.jsams.2011.09.003

Timperio, A., Duncan, S., Akram, M., Molina-Garcia, J., Van Dyck, D., Barnett, A., Salonna, F., Anjana, R. M., Sallis, J. F., Vorlícek, M., Hinckson, E., Cain, K., Conway, T., Muda, W. A. W., Moran, M., Oyeyemi, A. L., Pizzaro, A., Reis, R. S., Rezwan, S. M., Schipperijn, J., & Cerin, E. (accepted, 2025). Associations between parental perceptions of neighbourhood environments and active school travel: IPEN Adolescent study. International Journal of Behavioral Nutrition and Physical Activity.

van Buuren S. (2018). Flexible Imputation of Missing Data. 2nd ed. Boca Raton, Florida: Chapman and Hall/CRC. https://doi.org/10.1201/9780429492259

Wood SN. (2017). Generalized Additive Models: An Introduction with R. 2nd ed. Chapman and Hall/CRC. https://doi.org/10.1201/9781315370279

**Table S1.** Descriptive statistics of GIS-based destination and land-use mix (DLUM) indicators and other neighbourhood characteristics

|  | **All sites** | **Melbourne (AUS)** | **Ghent (BEL)** | **Curitiba (BRA)** | **Hong Kong (CHN)** | **Hradec Kralove (CZE)** | **Olomouc (CZE)** | **Odense (DNK)** | **Auckland (NZL)** | **Wellington (NZL)** | **Valencia (ESP)** | **Baltimore (USA)** | **Seattle (USA)** |
| --- | --- | --- | --- | --- | --- | --- | --- | --- | --- | --- | --- | --- | --- |
| ***N*** | 5052 | 422 | 271 | 493 | 1291 | 155 | 183 | 207 | 449 | 188 | 465 | 485 | 443 |
| ***DLUM 1: Ratio of land area of 5 land uses to residential land area*** | | | | |  |  |  |  |  |  |  |  |  |
| M (SD) | 108.7 (1515.9) | 1.30 (2.46) | 0.92 (1.25) | 1.44 (1.47) | 421.2 (2977.5) | 1.85 (2.72) | 0.51 (0.52) | 3.51 (5.34) | 1.14 (1.20) | 3.38 (6.88) | 0.70 (0.80) | 2.06 (9.78) | 0.73 (1.95) |
| Median (IQR) | 0.85 (1.44) | 0.67 (0.62) | 0.54 (0.82) | 1.01 (0.82) | 1.74 (2.67) | 0.97 (1.01) | 0.20 (0.90) | 1.71 (2.95) | 0.81 (0.93) | 1.43 (2.64) | 0.31 (0.77) | 0.31 (0.76) | 0.31 (0.52) |
| ***DLUM 2: Ratio of parcel counts of 5 land uses to dwelling unit count*** | | | | | |  |  |  |  |  |  |  |  |
| M (SD) | 0.05 (0.08) | 0.06 (0.04) | 0.04 (0.01) | 0.17 (0.06) | 0.04 (0.10) | 0.03 (0.02) | 0.05 (0.03) | 0.12 (0.07) | 0.05 (0.09) | 0.04 (0.06) | 0.01 (0.03) | 0.04 (0.08) | 0.02 (0.02) |
| Median (IQR) | 0.03 (0.05) | 0.05 (0.05) | 0.03 (0.02) | 0.15 (0.09) | 0.01 (0.01) | 0.02 (0.02) | 0.05 (0.03) | 0.12 (0.08) | 0.03 (0.02) | 0.02 (0.03) | 0.01 (0.01) | 0.02 (0.04) | 0.02 (0.02) |
| ***DLUM 3a: Parcel counts of 5 land uses*** | | | | |  |  |  |  |  |  |  |  |  |
| M (SD) | 140.2 (206.7) | 168.5 (202.3) | 159.0 (189.1) | 578.8 (235.4) | 70.2 (82.1) | 89.5 (93.2) | 101.6 (116.2) | 235.5 (311.8) | 35.8 (21.6) | 40.9 (44.3) | 163.0 (103.6) | 40.8 (51.9) | 40.1 (66.7) |
| Median (IQR) | 58.0 (138.0) | 109.0 (142.0) | 79.0 (205.0) | 526.0 (337.0) | 41.0 (68.0) | 61.0 (69.0) | 52.0 (106.0) | 122.0 (144.0) | 34.0 (23.0) | 26.0 (35.0) | 152.0 (101.0) | 22.0 (52.0) | 13.0 (51.0) |
| ***DLUM 3b: Gross density of parcels of 5 land uses (parcels/km^2^)*** | | | | | | |  |  |  |  |  |  |  |
| M (SD) | 164.3 (179.0) | 178.6 (144.8) | 159.1 (132.5) | 563.9 (182.2) | 140.6 (112.0) | 89.8 (73.2) | 135.9 (115.8) | 203.1 (190.0) | 101.9 (62.4) | 101.4 (114.2) | 136.8 (53.6) | 51.1 (60.0) | 40.6 (49.7) |
| Median (IQR) | 105.4 (128.4) | 138.9 (131.1) | 113.3 (155.6) | 533.5 (222.5) | 110.6 (102.6) | 68.0 (69.1) | 100.6 (137.5) | 135.3 (102.2) | 94.7 (51.5) | 68.2 (83.6) | 136.3 (53.5) | 32.3 (57.6) | 22.7 (51.6) |
| ***DLUM 4: Net density of parcels of 5 land uses (parcels/km^2^)*** | | | | | | | | |  |  |  |  |  |
| M (SD) | 460.4 (1139.9) | 296.2 (271.9) | 457.5 (310.4) | 784.5 (390.5) | 510.7 (1232.3) | 443.6 (347.6) | 1207.2 (459.8) | 267.8 (237.4) | 138.2 (126.2) | 116.1 (173.1) | 688.7 (494.3) | 473.6 (2854.7) | 117.7 (164.8) |
| Median (IQR) | 256.5 (488.7) | 217.7 (271.2) | 402.3 (422.5) | 753.5 (510.3) | 271.3 (332.5) | 414.3 (286.8) | 1184.9 (547.1) | 192.4 (207.8) | 108.1 (115.8) | 46.3 (109.8) | 621.8 (425.6) | 105.3 (234.4) | 60.3 (127.1) |
| ***DLUM 5: Area-based land use mix index (5 land uses)*** | | | | |  |  |  |  |  |  |  |  |  |
| M (SD) | 0.57 (0.26) | 0.70 (0.22) | 0.64 (0.20) | 0.64 (0.12) | 0.61 (0.27) | 0.49 (0.24) | 0.67 (0.17) | 0.48 (0.19) | 0.49 (0.25) | 0.39 (0.26) | 0.69 (0.20) | 0.35 (0.24) | 0.44 (0.28) |
| Median (IQR) | 0.63) (0.36) | 0.73 (0.11) | 0.69 (0.23) | 0.64 (0.16) | 0.69 (0.32) | 0.57 (0.30) | 0.67 (0.29) | 0.48 (0.34) | 0.52 (0.42) | 0.40 (0.50) | 0.78 (0.22) | 0.39 (0.46) | 0.50 (0.49) |
| ***DLUM 6: Parcel-count-based land use mix index (5 land uses)*** | | | | |  |  |  |  |  |  |  |  |  |
| M (SD) | 0.62 (0.20) | 0.73 (0.11) | 0.77 (0.20) | 0.47 (0.05) | 0.69 (0.20) | 0.75 (0.11) | 0.61 (0.13) | 0.60 (0.12) | 0.62 (0.17) | 0.71 (0.24) | 0.72 (0.10) | 0.40 (0.21) | 0.48 (0.26) |
| Median (IQR) | 0.67 (0.26) | 0.74 (0.28) | 0.77 (0.06) | 0.48 (0.07) | 0.75 (0.21) | 0.76 (0.08) | 0.63 (0.25) | 0.61 (0.13) | 0.66 (0.21) | 0.74 (0.28) | 0.72 (0.07) | 0.43 (0.25) | 0.56 (0.28) |
| ***DLUM 7: Ratio of land area of 5 land uses to residential land area weighted by area-based land use mix index [DLUM 1 * (1 + DLUM 5)]*** | | | | | | | | | | |  |  |  |
| M (SD) | 109.6 (1515.9) | 2.11 (3.58) | 1.35 (1.51) | 2.32 (2.25) | 423.2 (2977.5) | 2.33 (2.74) | 0.92 (0.99) | 4.76 (6.34) | 1.64 (1.54) | 4.32 (7.81) | 1.06 (1.06) | 2.33 (10.04) | 1.03 (2.25) |
| Median (IQR) | 1.38 (2.25) | 1.13 (1.12) | 0.87 (1.48) | 1.66 (1.39) | 3.07 (4.53) | 1.56 (1.63) | 0.34 (1.62) | 2.61 (4.19) | 1.23 (1.28) | 2.07 (3.03) | 0.57 (1.04) | 0.46 (1.10) | 0.48 (0.85) |
| ***DLUM 8: Ratio of parcel counts of 5 land uses to dwelling unit count weighted by parcel-count-based land use mix index [DLUM 2 * (1 + DLUM 6)]*** | | | | | | | | | | |  |  |  |
| M (SD) | 0.08 (0.12) | 0.11 (0.07) | 0.07 (0.02) | 0.25 (0.08) | 0.06 (0.16) | 0.05 (0.04) | 0.07 (0.05) | 0.20 (0.11) | 0.08 (0.15) | 0.06 (0.09) | 0.02 (0.04) | 0.05 (0.09) | 0.03 (0.04) |
| Median (IQR) | 0.04 (0.09) | 0.09 (0.08) | 0.06 (0.03) | 0.23 (0.12) | 0.01 (0.02) | 0.04 (0.03) | 0.07 (0.06) | 0.18 (0.11) | 0.05 (0.03) | 0.04 (0.05) | 0.01 (0.01) | 0.03 (0.05) | 0.03 (0.04) |
| ***DLUM 9a: Parcel counts of 5 land uses weighted by parcel-count-based land use mix index (parcels/km^2^) [DLUM 3a * (1 + DLUM 6)]*** | | | | | | | | | | |  |  |  |
| M (SD) | 225.3 (318.0) | 291.1 (350.0) | 281.2 (332.1) | 851.0 (345.6) | 123.0 (148.5) | 158.0 (165.1) | 172.0 (204.5) | 375.4 (491.4) | 59.2 (37.3) | 71.0 (74.6) | 280.8 (171.6) | 59.2 (81.6) | 63.3 (99.7) |
| Median (IQR) | 99.6 (231.4) | 190.6 (242.7) | 142.2 (365.6) | 764.3 (502.5) | 69.3 (115.1) | 107.5 (122.8) | 77.4 (197.8) | 194.0 (238.9) | 55.7 (39.9) | 47.0 (66.1) | 265.6 (173.4) | 31.7 (73.8) | 20.9 (87.6) |
| ***DLUM 9b: Gross density of parcels of 5 land uses weighted by parcel-count-based land use mix index (parcels/km^2^) [DLUM 3b * (1 + DLUM 6)]*** | | | | | | | | | | |  |  |  |
| M (SD) | 265.3 (272.2) | 308.3 (249.3) | 281.6 (232.9) | 829.8 (268.5) | 240.4 (186.1) | 158.3 (129.5) | 227.0 (205.7) | 323.5 (299.0) | 167.2 (105.9) | 175.1 (190.0) | 235.4 (87.5) | 73.0 (81.6) | 64.1 (75.3) |
| Median (IQR) | 178.9 (228.5) | 237.9 (225.5) | 205.5 (279.6) | 783.8 (310.1) | 196.4 (179.8) | 119.5 (119.1) | 146.6 (254.7) | 215.7 (174.2) | 156.1 (92.4) | 125.5 (162.0) | 229.4 (89.9) | 48.3 (83.6) | 37.2 (88.8) |
| ***DLUM 10: Net density of parcels of 5 land uses weighted by parcel-count-based land use mix index (parcels/km^2^) [DLUM 4 * (1 + DLUM 6)]*** | | | | | | | | | | | |  |  |
| M (SD) | 708.9 (1294.5) | 508.2 (459.9) | 805.6 (541.8) | 1154.4 (569.5) | 770.0 (1492.4) | 761.1 (542.2) | 1910.1 (641.5) | 422.7 (368.1) | 222.8 (192.9) | 194.9 (287.0) | 1171.1 (814.6) | 552.3 (2857) | 183.5 (266.6) |
| Median (IQR) | 426.2 (792.3) | 374.0 (467.6) | 698.1 (752.0) | 1119.9 (721.9) | 465.2 (547.9) | 740.8 (514.4) | 1877.5 (695.7) | 308.0 (316.4) | 169.4 (189.7) | 85.3 (178.2) | 1094.0 (723.9) | 142.7 (326.1) | 89.4 (210.8) |
| ***Gross density of commercial and office land use parcels (parcels/km^2^)*** | | | | | |  |  |  |  |  |  |  |  |
| M (SD) | 89.6 (141.3) | 102.1 (91.1) | 47.4 (38.1) | 440.2 (143.1) | 37.1 (77.7) | 46.0 (45.3) | 69.4 (58.6) | 139.6 (137.1) | 62.4 (43.0) | 31.4 (54.2) | 45.3 (24.0) | 36.6 (51.7) | 23.8 (38.5) |
| Median (IQR) | 40.4 (77.7) | 77.7 (89.2) | 39.0 (38.2) | 422.7 (184.5) | 4.1 (42.5) | 31.5 (39.4) | 57.3 (56.4) | 93.2 (66.7) | 57.9 (39.6) | 6.9 (44.6) | 43.3 (34.6) | 17.3 (49.3) | 8.3 (34.7) |
| ***Gross density of institutional/civic land use parcels (parcels/km^2^)*** | | | | | |  |  |  |  |  |  |  |  |
| M (SD) | 27.6 (33.8) | 28.1 (22.0) | 39.1 (29.1) | 21.7 (9.6) | 57.1 (45.7) | 12.6 (7.8) | 13.3 (13.8) | 29.5 (31.5) | 6.9 (7.2) | 21.0 (45.4) | 14.7 (5.5) | 9.1 (9.2) | 8.3 (6.8) |
| Median (IQR) | 16.4 (26.7) | 22.6 (25.3) | 28.6 (43.3) | 19.0 (8.4) | 48.7 (46.9) | 10.6 (10.9) | 7.2 (17.0) | 18.1 (11.7) | 5.4 (7.2) | 10.8 (14.8) | 13.5 (5.5) | 6.5 (10.2) | 7.5 (8.1) |
| ***Gross density of food-related land use parcels (parcels/km^2^)*** | | | | | |  |  |  |  |  |  |  |  |
| M (SD) | 27.9 (34.1) | 14.4 (17.8) | 62.2 (63.5) | 72.5 (28.9) | 25.0 (17.7) | 8.6 (9.7) | 45.8 (7.8) | 9.2 (16.3) | 5.9 (11.3) | 12.8 (16.1) | 63.0 (30.6) | 2.8 (4.6) | 5.7 (7.7) |
| Median (IQR) | 14.9 (40.4) | 9.8 (13.8) | 37.4 (70.5) | 68.6 (36.5) | 21.3 (22.4) | 6.1 (6.7) | 30.0 (53.1) | 4.2 (6.5) | 3.4 (7.7) | 7.7 (15.8) | 58.1 (19.9) | 0.8 (4.0) | 2.3 (9.4) |
| ***Gross density of entertainment land use parcels (parcels/km^2^)*** | | | | | |  |  |  |  |  |  |  |  |
| M (SD) | 7.9 (13.9) | 15.9 (22.7) | 1.8 (3.1) | 21.1 (6.7) | 9.6 (6.2) | 3.3 (5.5) | 4.4 (7.8) | 6.9 (7.3) | 6.9 (7.2) | 21.0 (45.4) | 0.4 (1.0) | 0.1 (0.3) | 0.2 (0.6) |
| Median (IQR) | 4.0 (11.7) | 9.8 (14.4) | 0.8 (2.0) | 20.2 (9.8) | 9.5 (6.8) | 1.1 (3.6) | 1.4 (7.6) | 5.1 (5.6) | 5.2 (7.2) | 10.8 (14.8) | 0.0 (0.6) | 0.0 (0.0) | 0.0 (0.0) |
| ***Density of parks (parks/km^2^)*** | | | | | |  |  |  |  |  |  |  |  |
| M (SD) | 11.3 (9.9) | 18.2 (13.0) | 8.6 (5.9) | 8.4 (7.5) | 11.8 (8.7) | 19.3 (10.4) | 2.9 (4.6) | 18.0 (8.7) | 19.8 (9.8) | 15.3 (8.6) | 13.4 (7.1) | 2.5 (2.9) | 2.7 (2.6) |
| Median (IQR) | 9.4 (12.8) | 15.1 (15.2) | 7.3 (8.6) | 6.3 (6.8) | 10.6 (11.7) | 19.9 (15.9) | 0.0 (5.3) | 16.4 (12.1) | 19.4 (10.8) | 14.5 (8.6) | 11.6 (6.3) | 1.5 (4.0) | 2.4 (3.8) |
| ***Net residential density (dwelling units/km^2^)*** | | | | | |  |  |  |  |  |  |  |  |
| M (SD) | 22774.4 (38296.6) | 2703.9 (735.5) | 7020.9 (5661.7) | 5196.6 (2729.7) | 67657.9 (52253.9) | 13883.9 (9421.1) | 12012.5 (9725.3) | 5741.0 (6955.4) | 2781.3 (1295.9) | 4556.3 (5285.2) | 26522.0 (8926.8) | 2894.1 (14582.2) | 1633.8 (2742.0) |
| Median (IQR) | 4230.1 (27987.8) | 2616.2 (816.2) | 4538.3 (10518.7) | 4174.2 (3531.2) | 68745.9 (57631.3) | 12434.1 (7945.6) | 8193.5 (14841.9) | 3026.8 (4037.9) | 2970.7 (1819.4) | 3536.5 (2901.6) | 29842.1 (6079.5) | 1622.3 (1148.7) | 1109.3 (1231.6) |
| ***Street-intersection density (intersections/km^2^)*** | | | | | |  |  |  |  |  |  |  |  |
| M (SD) | 134.6 (49.0) | 114.9 (22.2) | 155.5 (57.0) | 144.0 (39.6) | 121.3 (45.9) | 106.9 (17.4) | 86.5 (21.4) | 174.7 (41.2) | 146.9 (47.2) | 150.6 (42.3) | 196.7 (48.1) | 117.3 (36.9) | 114.6 (28.4) |
| Median (IQR) | 126.3 (58.4) | 114.1 (21.9) | 152.6 (81.7) | 133.4 (31.0) | 112.7 (58.1) | 108.8 (19.5) | 87.3 (32.0) | 176.8 (53.6) | 138.3 (50.4) | 143.2 (48.5) | 194.7 (32.1) | 114.2 (45.4) | 114.8 (37.1) |

*Notes.* AUS = Australia; BEL = Belgium; BRA = Brazil; CHN = China; CZE = Czech Republic; DNK = Denmark; NZL = New Zealand; ESP = Spain; USA = United States of America; M = mean; SD=standard deviation; IQR = interquartile range.

**Table S2.** Net residential and street intersection densities as mediators of between-site differences in destination and land-use mix indicators

| ***Between-site differences in net residential and street intersection density (associations between exposures and mediators)*** | | | | | |
| --- | --- | --- | --- | --- | --- |
|  | | **Net residential density (dwellings/km^2^)** | | **Street intersection density (intersections/km^2^)** | |
| **Site** [reference category: Melbourne (AUS)] | | **e*^b^* (95% CI)** | ***p*-value** | **e*^b^* (95% CI)** | ***p*-value** |
| Ghent (BEL) | | 1.82 (1.57, 2.07) | <.001 | 1.26 (1.20, 1.33) | <.001 |
| Curitiba (BRA) | | 1.63 (1.45, 1.83) | <.001 | 1.22 (1.17, 1.28) | <.001 |
| Hong Kong (CHN) | | 15.87 (12.4, 17.7) | <.001 | 1.14 (1.09, 1.19) | <.001 |
| Hradec Kralove (CZE) | | 4.08 (3.19, 5.22) | <.001 | 0.91 (0.83, 0.99) | .041 |
| Olomouc (CZE) | | 3.65 (3.05, 4.38) | <.001 | 0.82 (0.76, 0.87) | <.001 |
| Odense (DNK) | | 1.58 (1.33, 1.89) | <.001 | 1.50 (1.41, 1.61) | <.001 |
| Auckland (NZL) | | 0.92 (0.82, 1.03) | .143 | 1.25 (1.19, 1.30) | <.001 |
| Wellington (NZL) | | 1.34 (1.17, 1.54) | <.001 | 1.31 (1.24, 1.39) | <.001 |
| Valencia (ESP) | | 10.75 (9.50, 12.16) | <.001 | 1.80 (1.72, 1.89) | <.001 |
| Baltimore (USA) | | 0.64 (0.57, 0.71) | <.001 | 1.03 (0.99, 1.07) | .205 |
| Seattle (USA) | | 0.44 (0.39, 0.49) | <.001 | 1.00 (0.96, 1.04) | .882 |
|  | |  |  |  |  |
| ***Site-adjusted associations of net residential and street intersection density with destination and land-use mix indicators (exposure-adjusted associations between mediators and outcomes)*** | | | | | |
|  | | **Mediators** | | | |
|  | | **Net residential density (1000 dwellings/km^2^)** | | **Street intersection density (100 intersections/km^2^)** | |
| **Destination and land-use mix indicators (outcomes)** | **% site-level variance explained by mediators** | **e*^b^* or *b* (95% CI)** | ***p*-value** | **e*^b^* or *b* (95% CI)** | ***p*-value** |
| DLUM 1: Ratio of land area of 5 land uses to residential land area | 6.1% | 1.004 (1.002, 1.207) | <.001 | 1.082 (0.970, 1.207) | .157 |
| DLUM 2: Ratio of parcel counts of 5 land uses to 100 dwelling unit count | 39.0% | 0.993 (0.992, 0.994) | <.001 | 1.023 (0.961, 1.089) | .468 |
| DLUM 3a: Parcel counts of 5 land uses | 59.7% | 1.000 (0.999, 1.001) | .959 |  | <.001 |
| DLUM 3b: Gross density of parcels of 5 land uses (parcels/km^2^) | 8.5% | 1.000 (0.999, 1.000) | .299 |  | <.001 |
| DLUM 4: Net density of parcels of 5 land uses (parcels/km^2^) | 1.5% | 0.972 (0.968, 0.976) | <.001 |  | <.001 |
| DLUM 5: Area-based land use mix index (5 land uses)^#^ | 17.1% increase in city-level variance following adjustment for mediators | 0.0001 (-0.0001, 0.0004) | .252 |  | <.001 |
| DLUM 6: Parcel-count-based land use mix index (5 land uses)^#^ | 2.8% | 0.0004 (0.0002, 0.0005) | <.001 |  | <.001 |
| DLUM 7: Ratio of land area of 5 land uses to residential land area weighted by area-based land use mix index | 18.9% | 1.004 (1.002, 1.006) | <.001 | 1.170 (1.052, 1.301) | .004 |
| DLUM 8: Ratio of parcel counts of 5 land uses to 100 dwelling unit count weighted by parcel-count-based land use mix index | 47.0% | 0.993 (0.992, 0.994) | <.001 | 1.072 (1.006, 1.144) | .031 |
| DLUM 9a: Parcel counts of 5 land uses weighted by parcel-count-based land use mix index | 3.7% increase in city-level variance following adjustment for mediators | 1.004 (1.002, 1.005) | <.001 |  | <.001 |
| DLUM 9b: Gross density of parcels of 5 land uses weighted by parcel-count-based land use mix index (parcels/km^2^) | 0.7% increase in city-level variance following adjustment for mediators | 1.000 (0.999, 1.000) | .631 |  | <.001 |
| DLUM 10: Net density of parcels of 5 land uses weighted by parcel-count-based land use mix index (parcels/km^2^) | 4.6% increase in city-level variance following adjustment for mediators | 0.989 (0.987, 0.991) | <.001 | 0.977 (0.995, 1.010) | .171 |

*Notes.* AUS = Australia; BEL = Belgium; BRA = Brazil; CHN = China; CZE = Czech Republic; DNK = Denmark; NZL = New Zealand; ESP = Spain; USA = United States of America. ^#^ = for these destination and land-use mix indicators, values represent regression coefficients *b* and their respective 95% CIs (confidence intervals). For other destination and land-use mix indicators, values represent exponentiated regression coefficients (e*^b^*) and their respective 95% CIs. All models included study sites and neighbourhood types as covariates and accounted for neighbourhood and school level clustering effects arising from the participant recruitment strategies. Models used Gaussian, Gamma or negative binomial variance functions and identity or logarithmic link functions as appropriate.

**Table S3.** Associations between GIS-based destination and land-use mix (DLUM) indicators and parental perceived proximity of (overall) destinations from home

| Indicator | Note | Statistics | *p*-value |
| --- | --- | --- | --- |
| DLUM 1: Ratio of land area of 5 land uses to residential land area | Linear association moderated by site  [see Table S4] | Δ AIC = -134.0 | NA |
| DLUM 2: Ratio of parcel counts of 5 land uses to 100 dwelling units | Curvilinear association  [see Figure 11 – panel A] | F (6.2, 4392.8) = 13.4 | <.001 |
| DLUM 3a: Parcel counts of 5 land uses | Curvilinear association  [see Figure 12 – panel A] | F (8.2, 4390.8) = 111.4 | <.001 |
| DLUM 3b: Gross density of parcels of 5 land uses (parcels/km^2^) | Curvilinear association  [see Figure 12 – panel C] | F (8.0, 4391.0) = 109.1 | <.001 |
| DLUM 4: Net density of parcels of 5 land uses (parcels/km^2^) | Curvilinear association moderated by site [see Figure S2] | Δ AIC = -62.5 | NA |
| DLUM 5: Area-based land use mix index (5 land uses) | Linear association moderated by site [see Table S5] | Δ AIC = -26.7 | NA |
| DLUM 6: Parcel-count-based land use mix index (5 land uses) | Linear association moderated by site [see Table S5] | Δ AIC = -12.1 | NA |
| DLUM 7: Ratio of land area of 5 land uses to residential land area weighted by area-based land use mix index | Linear association moderated by site  [see Table S4] | Δ AIC = -50.0 | NA |
| DLUM 8: Ratio of parcel counts of 5 land uses to 100 dwelling units weighted by parcel-count-based land use mix index | Curvilinear association  [see Figure 11 – panel A] | F (6.5, 4392.5) = 16.8 | <.001 |
| DLUM 9a: Parcel counts of 5 land uses weighted by parcel-count-based land use mix index | Curvilinear association  [see Figure 12 – panel B] | F (8.4, 4390.6) = 114.4 | <.001 |
| DLUM 9b: Gross density of parcels of 5 land uses weighted by parcel-count-based land use mix index (parcels/km^2^) | Curvilinear association  [see Figure 12 – panel D] | F (7.9, 4391.1) = 118.6 | <.001 |
| DLUM 10: Net density of parcels of 5 land uses weighted by parcel-count-based land use mix index (parcels/km^2^) | Curvilinear association moderated by site [see Figure S3] | Δ AIC = -64.0 | NA |

*Notes.* All models included study sites, neighbourhood types, adolescent sex and age, parental marital status, persons in the household and highest education in the household as covariates and accounted for neighbourhood and school level clustering effects arising from the participant recruitment strategies. Models used Gaussian variance and identity link functions. NA = not applicable; Δ AIC = difference in Akaike information criterium values between regression models with and without interaction terms of site by DLUM. F = F-ratio values of smooth term (modelling curvilinear relationships).

**Table S4.** Associations of ratio of land area of 5 land uses to residential land area indicators with parental perceived proximity of destinations from home by study site

| City (country) | DLUM 1: ratio of land area of 5 land uses to residential land area unweighted for area-based land use mix index | | DLUM 7: ratio of land area of 5 land uses to residential land area weighted for area-based land use mix index | |
| --- | --- | --- | --- | --- |
|  | *b* (95% CI) | *p*-value | *b* (95% CI) | *p*-value |
| Melbourne (AUS) | -0.03 (-0.06, -0.001) | .046 | -0.02 (-0.04, 0.01) | .136 |
| Ghent (BEL) | 0.15 (0.06, 0.24) | .001 | 0.17 (0.09, 0.24) | <.001 |
| Curitiba (BRA) | -0.002 (-0.06, 0.05) | .946 | 0.002 (-0.03, 0.04) | .920 |
| Hong Kong (CHN) | -0.0004 (-0.001, 0.0001) | .119 | -0.0004 (-0.001, 0.0001) | .132 |
| Hradec Kralove (CZE) | 0.02 (-0.06, 0.10) | .606 | 0.03 (-0.04, 0.11) | .362 |
| Olomouc (CZE) | 0.68 (0.36, 1.01) | <.001 | 0.37 (0.20, 0.55) | <.001 |
| Odense (DNK) | 0.03 (0.003, 0.05) | .029 | 0.03 (0.01, 0.05) | .006 |
| Valencia (ESP) | 0.06 (-0.04, 0.17) | .239 | 0.06 (-0.02, 0.14) | .157 |
| Baltimore (USA) | 0.004 (-0.003, 0.01) | .221 | 0.005 (-0.002, 0.01) | .172 |
| Seattle (USA) | -0.01 (-0.05, 0.03) | .562 | 0.01 (-0.02, 0.04) | .575 |

*Notes.* AUS = Australia; BEL = Belgium; BRA = Brazil; CHN = China; CZE = Czech Republic; DNK = Denmark; ESP = Spain; USA = United States of America. *b* = regression coefficient; CI = confidence intervals. All models included cities, neighbourhood types, adolescent sex and age, parental marital status, persons in the household and highest education in the household as covariates and accounted for neighbourhood and school level clustering effects arising from the participant recruitment strategies. Models used Gaussian varianceand identity link functions.

**Table S5.** Associations of ratio of area-based and parcel-count-based land use mix indices with parental perceived proximity of destinations from home by study site

| City (country) | DLUM 5: Area-based land use mix index | | DLUM 6: Parcel-count-based land use mix index | |
| --- | --- | --- | --- | --- |
|  | *b* (95% CI) | *p*-value | *b* (95% CI) | *p*-value |
| Melbourne (AUS) | 0.85 (0.45, 1.15) | <.001 | 1.05 (0.86, 1.23) | <.001 |
| Ghent (BEL) | 0.81 (0.30, 1.31) | .002 | 1.33 (1.15, 1.51) | <.001 |
| Curitiba (BRA) | 0.03 (-0.56, 0.63) | .930 | 1.43 (1.18, 1.68) | <.001 |
| Hong Kong (CHN) | 0.79 (0.62, 0.95) | <.001 | 1.45 (1.31, 1.59) | <.001 |
| Hradec Kralove (CZE) | 0.93 (0.20, 1.66) | .013 | 1.10 (0.80, 1.40) | <.001 |
| Olomouc (CZE) | 2.09 (1.11, 3.07) | <.001 | 1.34 (1.04, 1.64) | <.001 |
| Odense (DNK) | 1.20 (0.64, 1.76) | <.001 | 1.41 (1.15, 1.66) | <.001 |
| Valencia (ESP) | 0.43 (0.03, 0.84) | .036 | 2.52 (2.35, 2.70) | <.001 |
| Baltimore (USA) | 1.26 (0.98, 1.54) | <.001 | 1.08 (0.86, 1.30) | <.001 |
| Seattle (USA) | 1.41 (1.15, 1.66) | <.001 | 1.07 (0.89, 1.26) | <.001 |

*Notes.* AUS = Australia; BEL = Belgium; BRA = Brazil; CHN = China; CZE = Czech Republic; DNK = Denmark; ESP = Spain; USA = United States of America. *b* = regression coefficient; CI = confidence intervals. All models included cities, neighbourhood types, adolescent sex and age, parental marital status, persons in the household and highest education in the household as covariates and accounted for neighbourhood and school level clustering effects arising from the participant recruitment strategies. Models used Gaussian variance and identity link functions.

**Table S6.** Associations of GIS-based destination and land-use mix (DLUM) indicators with adolescents’ non-school active transport (weekly frequency) by study site (only significant moderation effects reported)

| City (country) | DLUM 2: Ratio of parcel counts of 5 land uses to 100 dwelling unit count | | DLUM 8: Ratio of parcel counts of 5 land uses to 100 dwelling unit count weighted by parcel-count-based land use mix index | | DLUM 4: Net density of parcels of 5 land uses (parcels/km^2^) | | DLUM 10: Net density of parcels of 5 land uses weighted by parcel-count-based land use mix index (parcels/km^2^) | |
| --- | --- | --- | --- | --- | --- | --- | --- | --- |
|  | e*^b^* (95% CI) | *p*-value | e*^b^* (95% CI) | *p*-value | e*^b^* (95% CI) | *p*-value | e*^b^* (95% CI) | *p*-value |
| Melbourne (AUS) | 1.004 (0.988, 1.020) | .645 | 1.003 (0.994, 1.012) | .523 | 1.000 (0.9997, 1.0002) | .785 | 1.000 (0.9998, 1.0001) | .842 |
| Ghent (BEL) | 1.023 (0.958, 1.092) | .497 | 1.014 (0.977, 1.052) | .469 | 1.000 (0.9998, 1.0002) | .614 | 1.000 (0.9999, 1.0002) | .592 |
| Curitiba (BRA) | NA |  | NA |  | NA |  | NA |  |
| Hong Kong (CHN) | 1.003 (0.997, 1.009) | .290 | 1.002 (0.998, 1.006) | .303 | 1.000 (0.999, 1.001) | .294 | 1.000 (0.999, 1.001) | .362 |
| Hradec Kralove (CZE) | 0.945 (0.894, 0.999) | .046 | 0.969 (0.940, 0.999) | .047 | 1.000 (0.999, 1.000) | .142 | 1.000 (0.999, 1.000) | .154 |
| Olomouc (CZE) | 0.998 (0.962, 1.036) | .931 | 0.999 (0.977, 1.020) | .901 | 1.000 (0.999, 1.000) | .317 | 1.000 (0.999, 1.000) | .291 |
| Odense (DNK) | 0.992 (0.978, 1.005) | .221 | 0.995 (0.986, 1.004) | .284 | 1.000 (0.999, 1.001) | .187 | 1.000 (0.999, 1.000) | .207 |
| Auckland (NZL) | 1.003 (0.996, 1.010) | .471 | 1.001 (0.997, 1.006) | .521 | 1.000 (0.999, 1.001) | .868 | 1.000 (0.999, 1.001) | .921 |
| Wellington (NZL) | 0.999 (0.983, 1.016) | .934 | 1.000 (0.990, 1.010) | .990 | 0.999 (0.999, 1.000) | .688 | 0.999 (0.999, 1.000) | .662 |
| Valencia (ESP) | NA |  | NA |  | NA |  | NA |  |
| Baltimore (USA) | 0.994 (0.986, 1.002) | .152 | 0.997 (0.990, 1.003) | .307 | 1.000 (0.999, 1.000) | .899 | 1.000 (0.999, 1.000) | .986 |
| Seattle (USA) | 1.070 (1.041, 1.100) | <.001 | 1.048 (1.030, 1.066) | <.001 | 1.0010 (1.0006, 1.0014) | <.001 | 1.0006 (1.0004, 1.001) | <.001 |

*Notes.* AUS = Australia; BEL = Belgium; BRA = Brazil; CHN = China; CZE = Czech Republic; DNK = Denmark; NZL = New Zealand; ESP = Spain; USA = United States of America. e*^b^* = exponentiated values of regression coefficient; CI = confidence intervals; NA = not applicable as data on this physical activity outcome was not collected. All models included cities, neighbourhood types, adolescent sex and age, parental marital status, persons in the household and highest education in the household as covariates and accounted for neighbourhood and school level clustering effects arising from the participant recruitment strategies. Models used negative binomial variance and logarithmic link functions.

**Table S7.** Associations of GIS-based destination and land-use mix (DLUM) indicators with adolescents’ accelerometer-based total moderate-to-vigorous physical activity (MVPA) (average min/day) by study site (only significant moderation effects reported)

| City (country) | DLUM 3a: Parcel count of 5 land uses | | DLUM 9a: Parcel counts of 5 land uses weighted by parcel-count-based land use mix index | | DLUM 3b: Gross density of parcels of 5 land uses (parcels/km^2^) | | DLUM 9b: Gross density of parcels of 5 land uses weighted by parcel-count-based land use mix index (parcels/km^2^) | |
| --- | --- | --- | --- | --- | --- | --- | --- | --- |
|  | e*^b^* (95% CI) | *p*-value | e*^b^* (95% CI) | *p*-value | e*^b^* (95% CI) | *p*-value | e*^b^* (95% CI) | *p*-value |
| Melbourne (AUS) | 1.001 (1.000, 1.001) | <.001 | 1.001 (1.000, 1.001) | <.001 | 1.001 (1.000, 1.001) | <.001 | 1.001 (1.000, 1.001) | <.001 |
| Ghent (BEL) | 1.000 (0.999, 1.001) | .548 | 1.000 (0.999, 1.003) | .548 | 1.000 (0.999, 1.001) | .395 | 1.000 (0.999, 1.001) | .407 |
| Curitiba (BRA) | 1.000 (0.999, 1.001) | .560 | 1.000 (0.999, 1.001) | .537 | 1.000 (0.999, 1.001) | .811 | 1.000 (0.999, 1.001) | .790 |
| Hong Kong (CHN) | 0.999 (0.998, 1.000) | .078 | 0.999 (0.999, 1.000) | .054 | 0.999 (0.999, 1.000) | .880 | 1.000 (0.999, 1.001) | .361 |
| Hradec Kralove (CZE) | 1.000 (0.999, 1.001) | .909 | 1.000 (0.999, 1.001) | .895 | 1.000 (0.998, 1.002) | .968 | 1.000 (0.999, 1.001) | .987 |
| Olomouc (CZE) | 0.999 (0.998, 1.000) | .120 | 0.999 (0.999, 1.000) | .130 | 0.999 (0.998, 1.000) | .129 | 0.999 (0.999, 1.000) | .141 |
| Odense (DNK) | 1.000 (0.999, 1.001) | .605 | 1.000 (0.999, 1.000) | .588 | 1.000 (0.999, 1.001) | .504 | 1.000 (0.999, 1.001) | .489 |
| Auckland (NZL) | 1.000 (0.998, 1.002) | .634 | 1.000 (0.999, 1.001) | .749 | 1.000 (0.999, 1.001) | .781 | 1.000 (0.999, 1.001) | .917 |
| Wellington (NZL) | 1.000 (0.999, 1.002) | .634 | 1.000 (0.999, 1.001) | .731 | 1.000 (0.999, 1.001) | .247 | 1.000 (0.999, 1.001) | .323 |
| Valencia (ESP) | 1.000 (0.999, 1.000) | .431 | 1.000 (0.999, 1.000) | .509 | 0.999 (0.999, 1.001) | .292 | 0.999 (0.999, 1.001) | .324 |
| Baltimore (USA) | 1.001 (1.000, 1.002) | .035 | 1.001 (1.000, 1.002) | .030 | 1.001 (0.999, 1.001) | .142 | 1.001 (0.999, 1.001) | .104 |
| Seattle (USA) | 1.001 (1.000, 1.002) | .003 | 1.001 (1.000, 1.002) | .003 | 1.001 (1.000, 1.002) | .002 | 1.001 (1.000, 1.002) | .003 |

*Notes.* AUS = Australia; BEL = Belgium; BRA = Brazil; CHN = China; CZE = Czech Republic; DNK = Denmark; NZL = New Zealand; ESP = Spain; USA = United States of America. e*^b^* = exponentiated value of regression coefficient; CI = confidence intervals. All models included cities, neighbourhood types, adolescent sex and age, parental marital status, persons in the household and highest education in the household, valid days and time of accelerometer wear as covariates and accounted for neighbourhood and school level clustering effects arising from the participant recruitment strategies. Models used Gamma variance and logarithmic link functions.

**Table S8.** Associations between area-based land-use mix index (DLUM 5) and regular active transport to/from school by distance from home to school

| Distance from home to school | OR | 95% CI | *p*-value |
| --- | --- | --- | --- |
| 1-5 minute walk | 6.144 | (2.396, 15.756) | <.001 |
| 6-10 minute walk | 4.239 | (2.152, 8.349) | <.001 |
| 11-20 minute walk | 2.924 | (1.851, 4.618) | <.001 |
| 21-30 minute walk | 2.017 | (1.397, 2.912) | <.001 |
| 31+ minute walk | 1.392 | (0.855, 2.266) | .184 |

*Notes.* OR = odds ratio; CI = confidence intervals. All models included cities, neighbourhood types, adolescent sex and age, parental marital status, persons in the household and highest education in the household as covariates and accounted for neighbourhood and school level clustering effects arising from the participant recruitment strategies. Models used binomial variance and logit link functions.

**Table S9.** Associations of gross parcel densities of specific land uses (parcels/km^2^) with adolescents’ physical activity (PA)

| Land use | Total non-school PA^1^ | Regular walking to/from school (Y/N) ^2^ | Regular active transport to/from school (Y/N) ^2^ | Non-school active transport  (weekly frequency) | Total MVPA (average min/day) | MVPA during non-school periods (average min/day) |
| --- | --- | --- | --- | --- | --- | --- |
|  | e*^b^*  (95% CI) | OR  (95% CI) | OR  (95% CI) | e*^b^*  (95% CI) | e*^b^*  (95% CI) | e*^b^*  (95% CI) |
| Commercial / office | 1.000  (0.999, 1.002) | 0.999  (0.998, 1.001) | 0.999  (0.998, 1.001) | 1.001^*^  (1.000, 1.002) | 1.001^*^  (1.000, 1.001) | 1.000  (0.999, 1.000) |
| Institutional / civic | 1.001^*^  (1.000, 1.002) | 1.001  (0.997, 1.004) | 1.002  (0.998, 1.005) | Curvilinear^**^  (Figure S5A) | 1.001^*^  (1.000, 1.002) | 0.999  (0.998, 1.001) |
| Entertainment | 0.999  (0.998, 1.001) | 1.001  (0.994, 1.008) | 1.003  (0.994, 1.012) | 0.999  (0.997, 1.001) | 1.000  (0.999, 1.001) | 1.000  (0.999, 1.000) |
| Food-related | 1.000  (0.999, 1.001) | Moderated by DTS  (Table S10) | Moderated by DTS  (Table S10) | Curvilinear^*^  (Figure S5B) | 0.999  (0.998, 1.000) | 1.000  (0.999, 1.000) |
| Parks | 0.998  (0.997, 1.000) | 0.987^*^  (0.977, 0.998) | 0.994  (0.984, 1.004) | Moderated by site (Table S11) | 0.998  (0.996, 1.000) | 1.000  (0.999, 1.000) |

*Notes.* MVPA = moderate-to-vigorous PA; DTS = distance to school (from home); OR = odds ratio; CI = confidence intervals; e*^b^* = exponentiated value of regression coefficient. ^1^Average number of days per week with at least 60 min/day of PA (excluding PE & gym classes at school). ^2^ 5+ trips per week. ^*^*p*<.05; ^**^*p*<.01

All models included cities, neighbourhood types, adolescent sex and age, parental marital status, persons in the household and highest education in the household as covariates and accounted for neighbourhood and school level clustering effects arising from the participant recruitment strategies. All gross parcel density variables were included in the models (mutual adjustment). Models of MVPA were also adjusted for valid days and time of accelerometer wear.

**Table S10.** Associations of gross density of food-related parcels (parcels/km^2^) with adolescents’ active transport to/from school outcomes by distance from home to school

| Distance from home to school | Regular walking to/from school (Y/N) ^1^ | | | Regular active transport to/from school (Y/N) ^1^ | | |
| --- | --- | --- | --- | --- | --- | --- |
|  | OR | 95% CI | *p*-value | OR | 95% CI | *p*-value |
| 1-5 minute walk | 1.014 | (1.006, 1.022) | <.001 | 1.015 | (1.004, 1.026) | .008 |
| 6-10 minute walk | 1.011 | (1.004, 1.017) | .001 | 1.010 | (1.001, 1.019) | .023 |
| 11-20 minute walk | 1.007 | (1.002, 1.013) | .008 | 1.005 | (0.998, 1.012) | .135 |
| 21-30 minute walk | 1.004 | (0.999, 1.009) | .140 | 1.000 | (0.995, 1.006) | .942 |
| 31+ minute walk | 1.000 | (0.995, 1.006) | .891 | 0.995 | (0.989, 1.001) | .122 |

*Notes.* OR = odds ratio; CI = confidence intervals. ^1^ 5+ trips per week. All models included cities, neighbourhood types, adolescent sex and age, parental marital status, persons in the household and highest education in the household as covariates and accounted for neighbourhood and school level clustering effects arising from the participant recruitment strategies. All gross parcel density variables were included in the models (mutual adjustment).

**Table S11.** Associations of gross density of parks (parks/km^2^) with adolescents’ non-school active transport (weekly frequency) by study site

| City (country) | e*^b^* (95% CI) | *p*-value |
| --- | --- | --- |
| Melbourne (AUS) | 1.002 (0.997, 1.007) | .524 |
| Ghent (BEL) | 0.996 (0.980, 1.012) | .646 |
| Curitiba (BRA) | NA |  |
| Hong Kong (CHN) | 1.001 (0.996, 1.006) | .574 |
| Hradec Kralove (CZE) | 0.983 (0.972, 0.994) | .002 |
| Olomouc (CZE) | 0.979 (0.952, 1.006) | .129 |
| Odense (DNK) | 0.998 (0.985, 1.011) | .750 |
| Auckland (NZL) | 0.995 (0.988, 1.001) | .124 |
| Wellington (NZL) | 0.992 (0.980, 1.003) | .160 |
| Valencia (ESP) | NA |  |
| Baltimore (USA) | 1.024 (1.003, 1.046) | .027 |
| Seattle (USA) | 1.038 (1.012, 1.064) | .004 |

*Notes.* e*^b^* = exponentiated value of regression coefficient; CI = confidence intervals. All models included cities, neighbourhood types, adolescent sex and age, parental marital status, persons in the household and highest education in the household as covariates and accounted for neighbourhood and school level clustering effects arising from the participant recruitment strategies. All gross parcel density variables were included in the models (mutual adjustment).

***
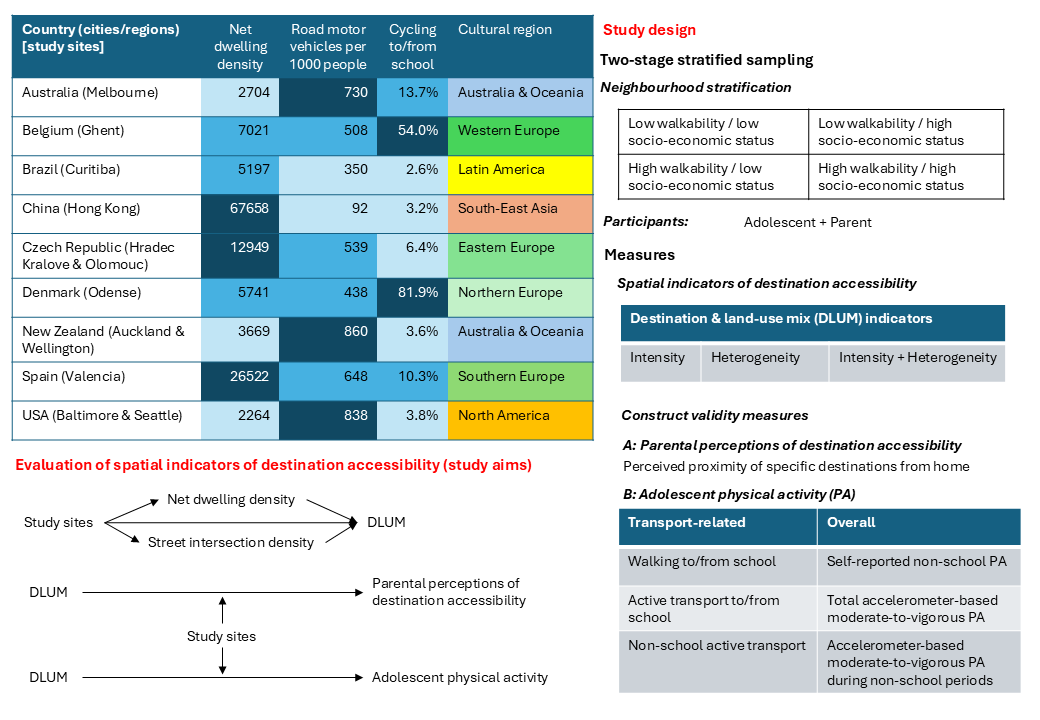
***

**Figure S1.** Overview of study sites, design and aims

**Figure S2.** Correlation matrix of destination and land-use mix indicators (Pearson’s correlation coefficients). DLUM1 = Ratio of land area of 5 land uses to residential land area; DLUM2 = Ratio of parcel counts of 5 land uses to dwelling unit count; DLUM3a = Parcel counts of 5 land uses; DLUM3b = Gross density of parcels of 5 land uses (parcels/km^2^); DLUM4 = Net density of parcels of 5 land uses (parcels/km^2^); DLUM5 = Area-based land use mix index (5 land uses); DLUM6 = Parcel-count-based land use mix index (5 land uses); DLUM7 = Ratio of land area of 5 land uses to residential land area weighted by area-based land use mix index; DLUM8 = Ratio of parcel counts of 5 land uses to dwelling unit count weighted by parcel-count-based land use mix index; DLUM9a = Parcel counts of 5 land uses weighted by parcel-count-based land use mix index; DLUM9b = Gross density of parcels of 5 land uses weighted by parcel-count-based land use mix index (parcels/km^2^); DLUM10 = Net density of parcels of 5 land uses weighted by parcel-count-based land use mix index (parcels/km^2^).


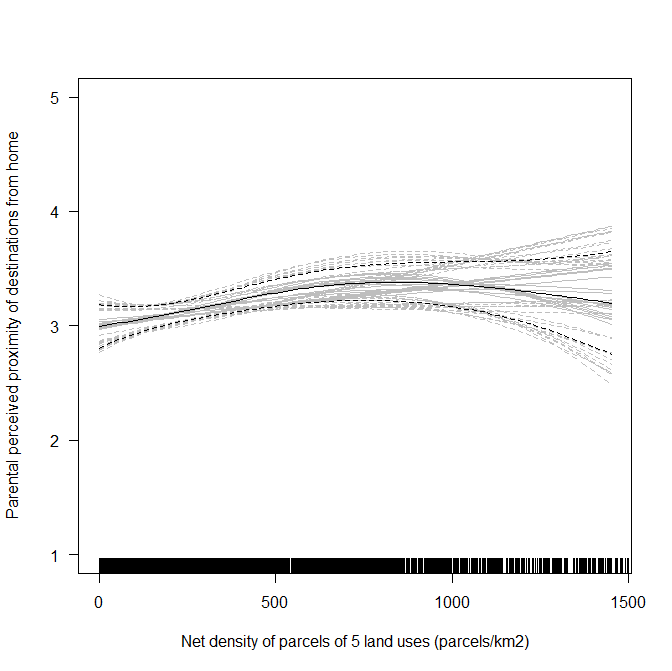

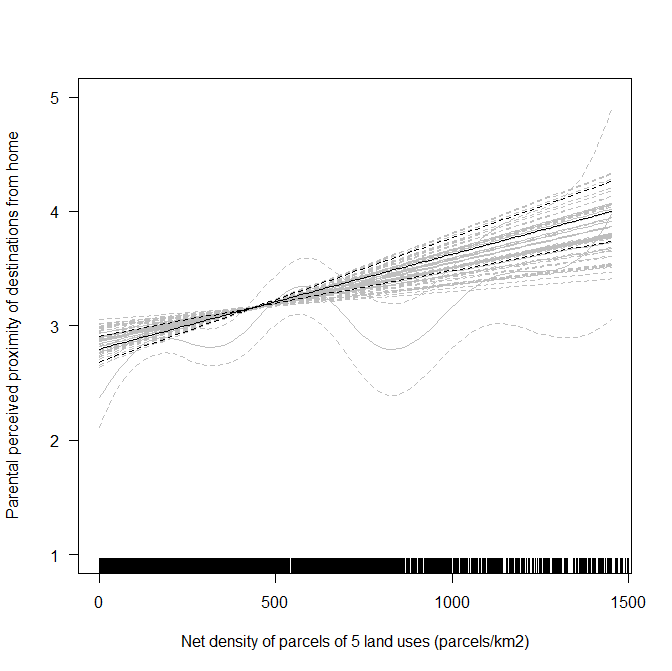


Ghent (BEL)

Melbourne (AUS)


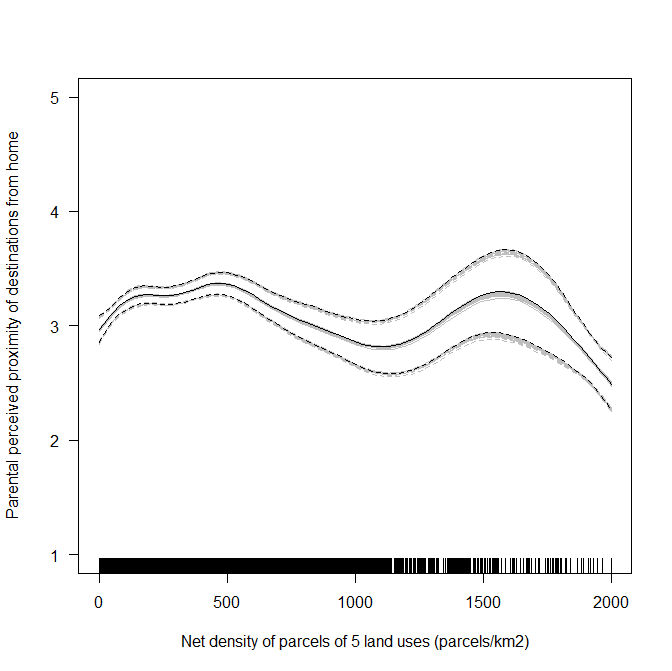

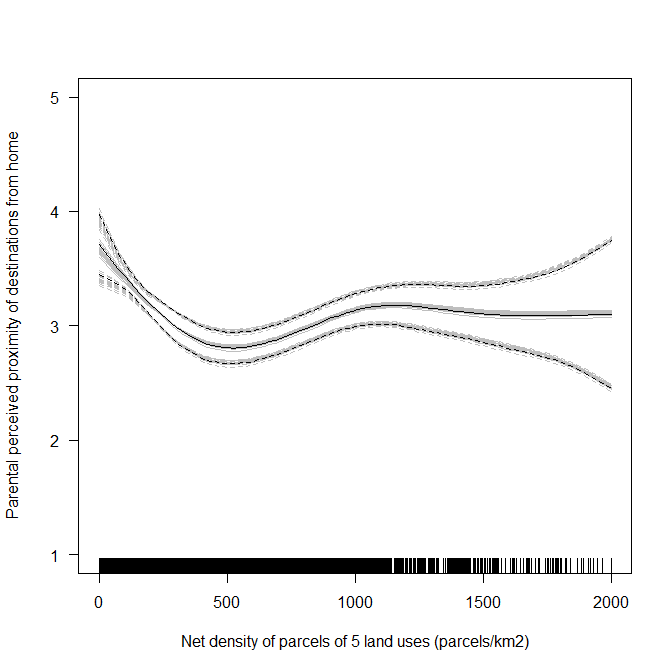


Hong Kong (CHN)

Curitiba (BRA)


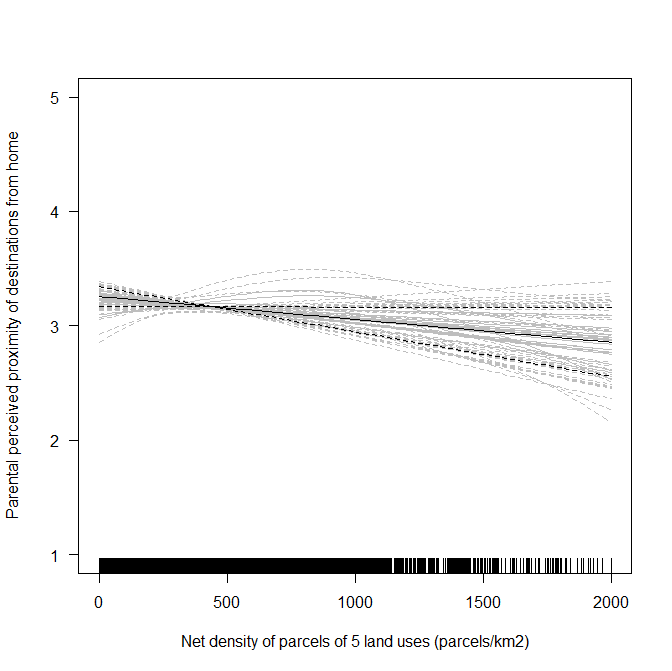

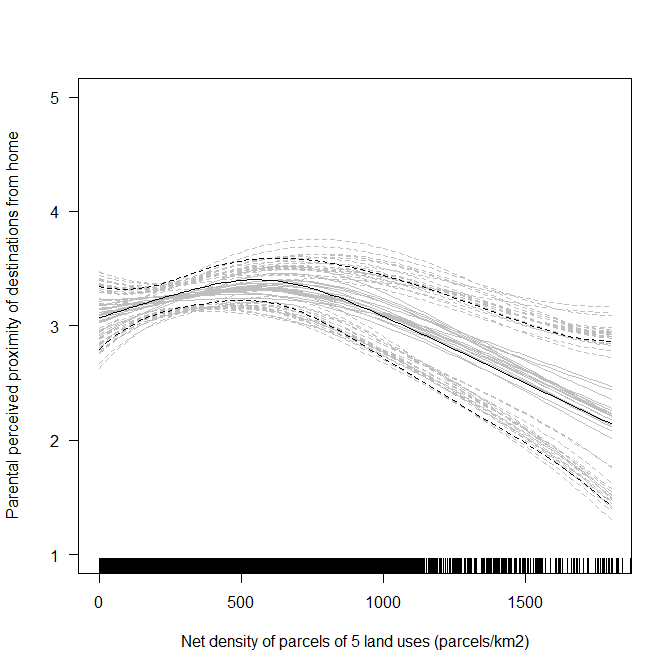


Olomouc (CZE)

Hradec Kralove (CZE)

**Figure S3.** Associations of net density of parcels of 5 land uses indicators (parcels/km^2^) unweighted for parcel-count-based land use mix index (DLUM 4) with parental perceived proximity of destinations from home by study site (continued on next page).


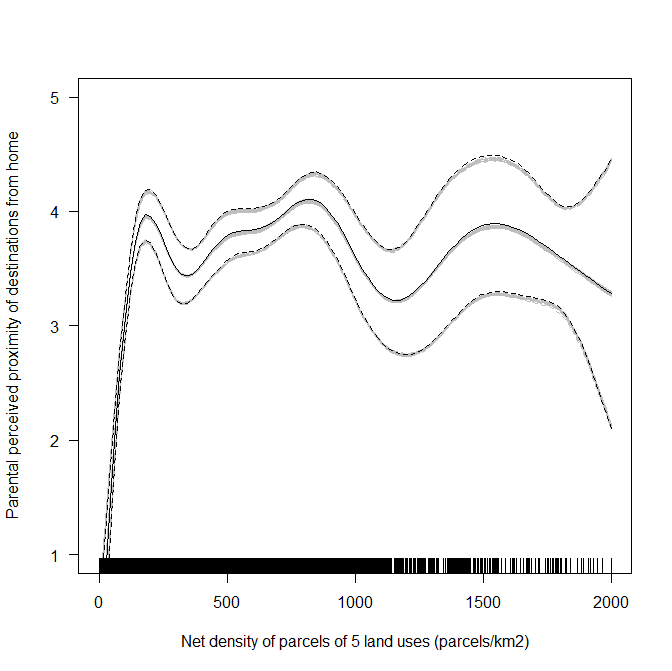

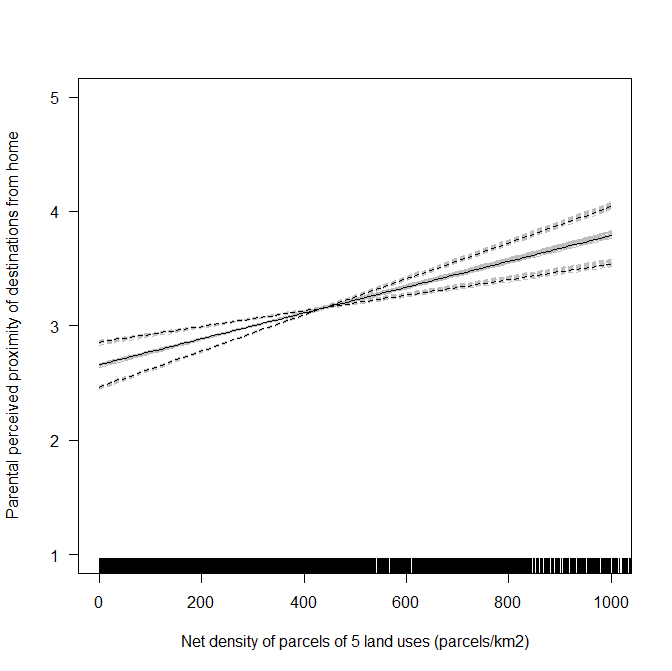


Valencia (ESP)

Odense (DNK)


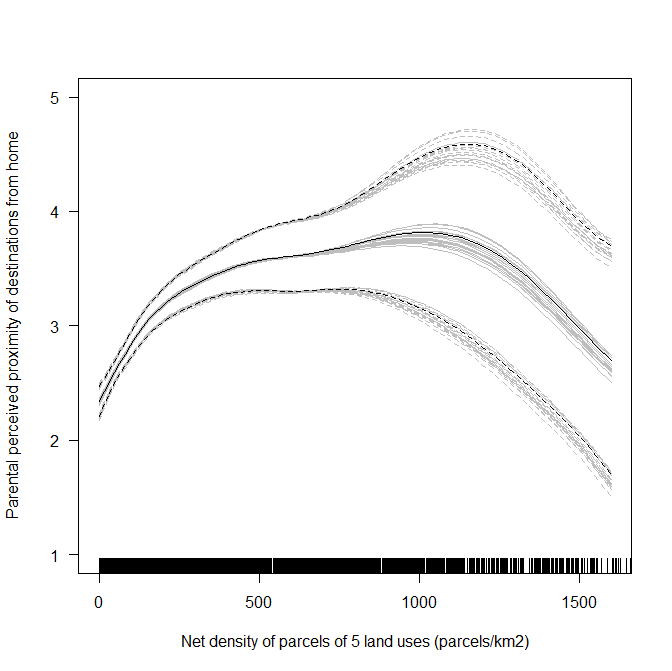


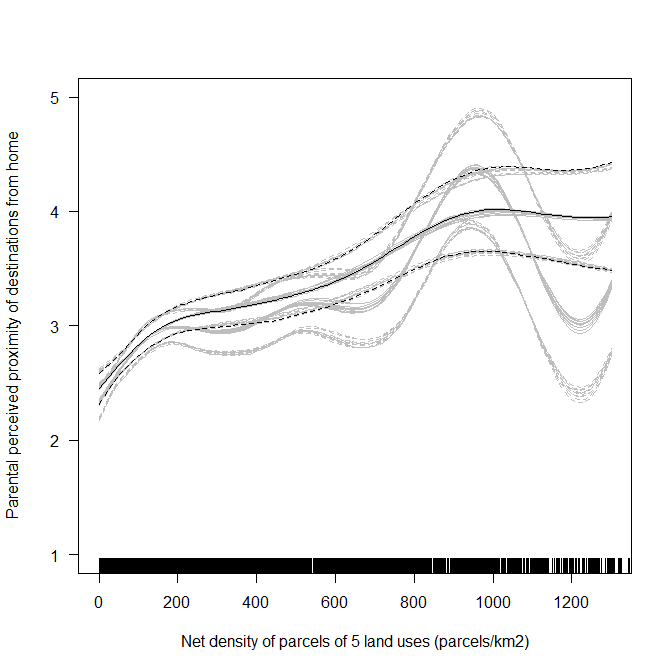


Seattle (USA)

Baltimore (USA)

**Figure S3.** Associations of net density of parcels of 5 land uses indicators (parcels/km^2^) unweighted for parcel-count-based land use mix index (DLUM 4) with parental perceived proximity of destinations from home by study site


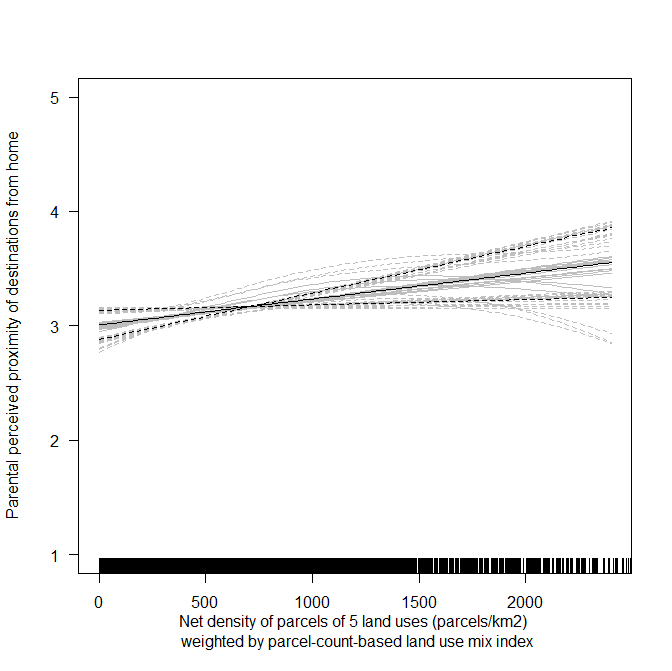

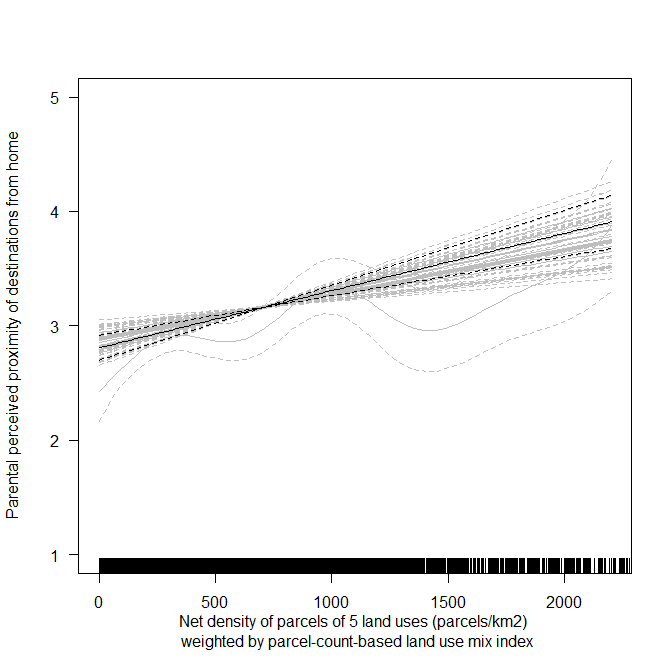


Ghent (BEL)

Melbourne (AUS)


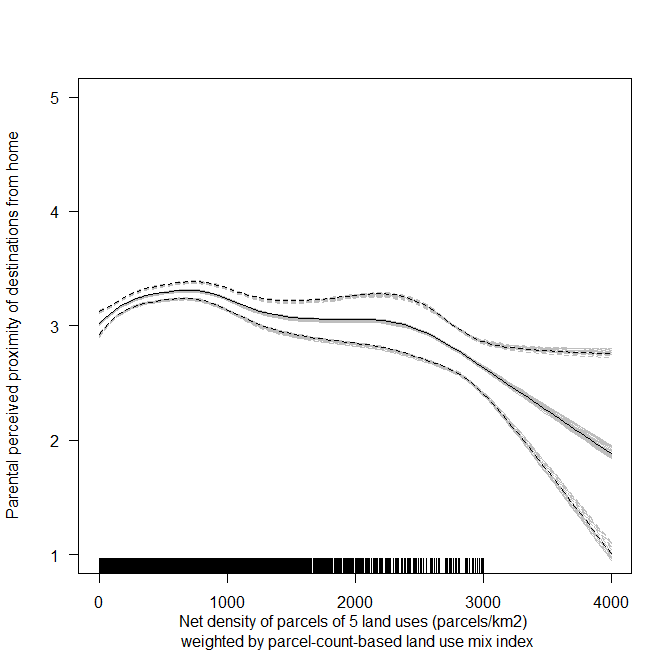

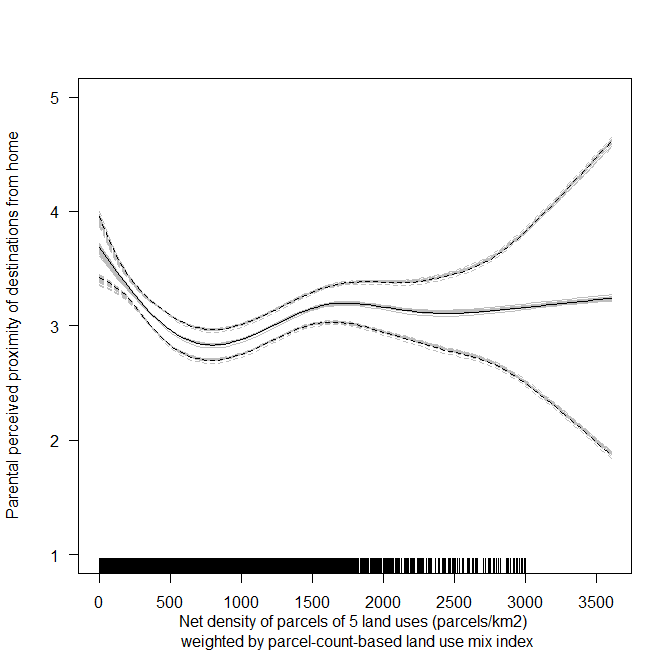


Hong Kong (CHN)

Curitiba (BRA)


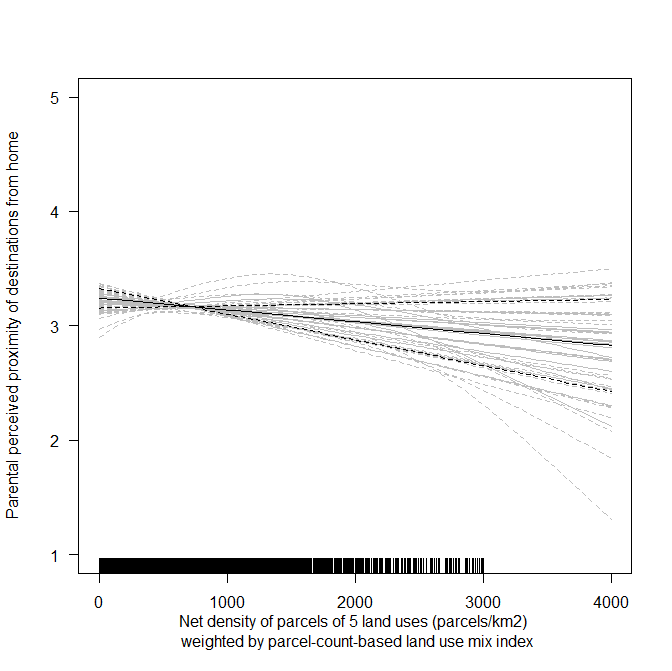

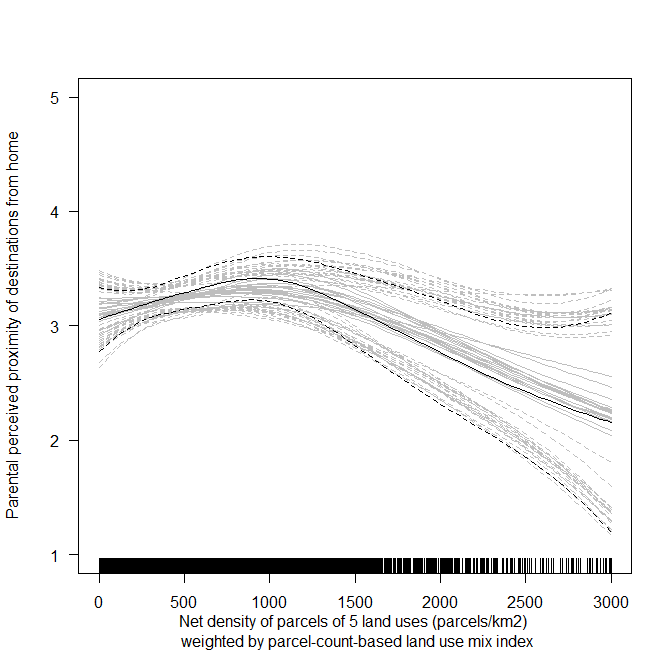


Olomouc (CZE)

Hradec Kralove (CZE)

**Figure S4.** Associations of net density of parcels of 5 land uses indicators (parcels/km^2^) weighted for parcel-count-based land use mix index (DLUM 10) with parental perceived proximity of destinations from home by study site (continued on next page).


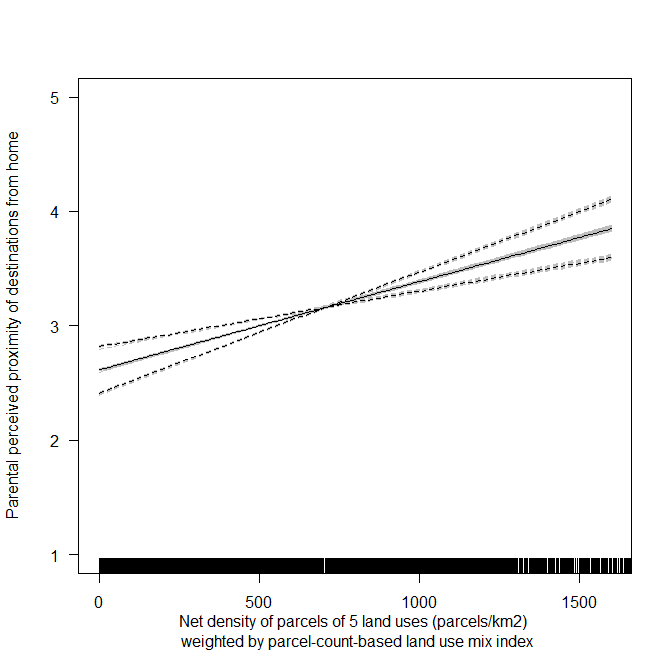


Valencia (ESP)

Odense (DNK)


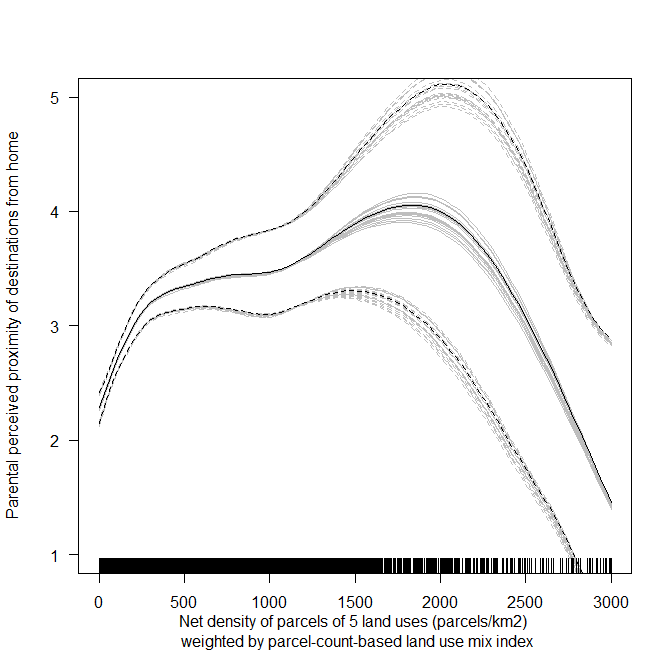

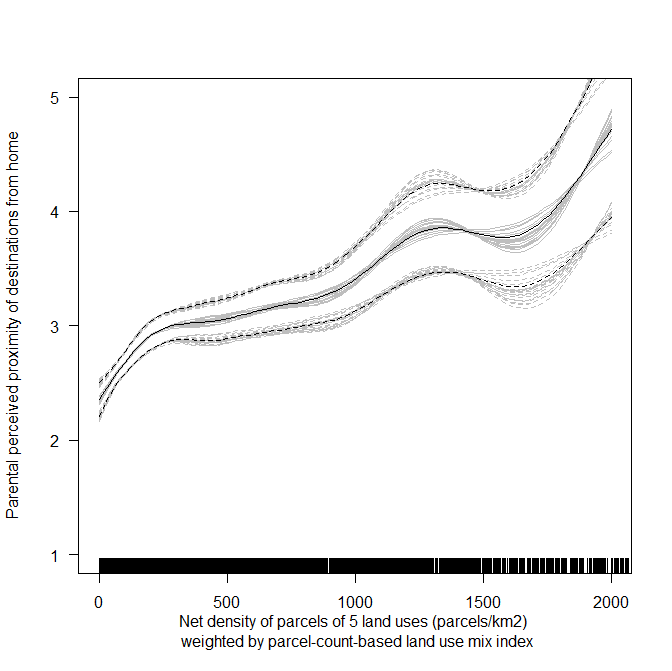


Seattle (USA)

Baltimore (USA)

**Figure S4.** Associations of net density of parcels of 5 land uses indicators (parcels/km^2^) weighted for parcel-count-based land use mix index (DLUM 10) with parental perceived proximity of destinations from home by study site


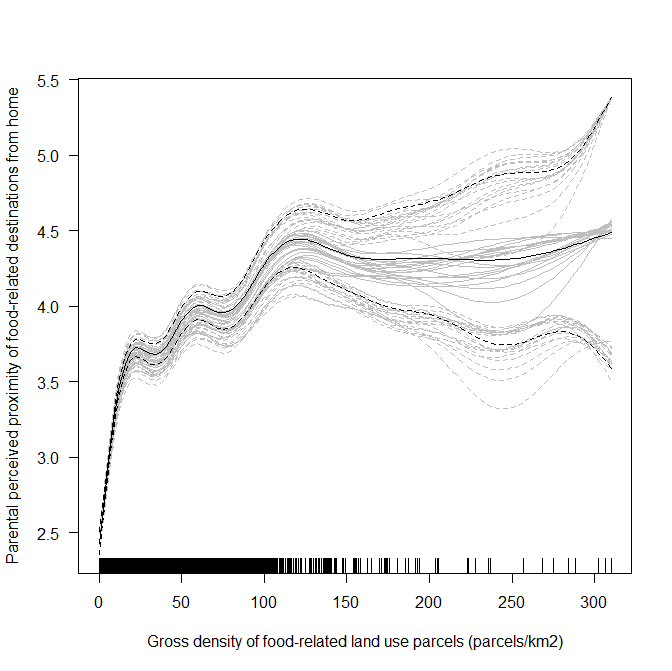

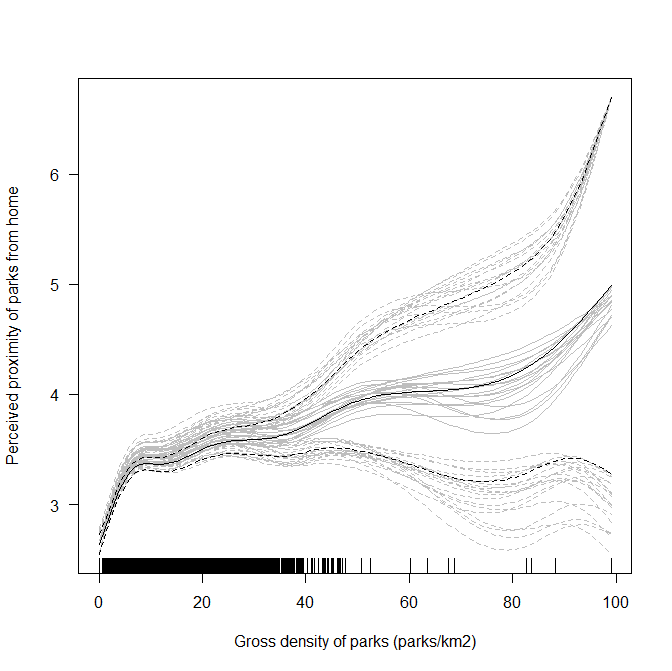

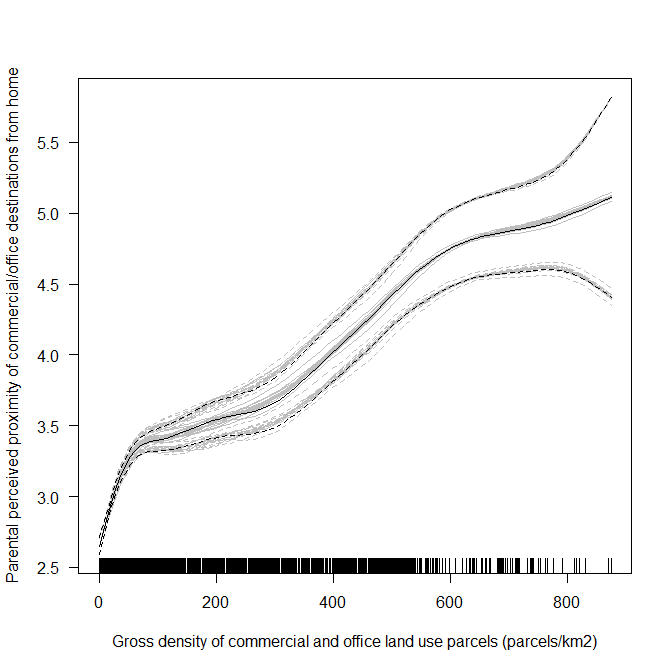

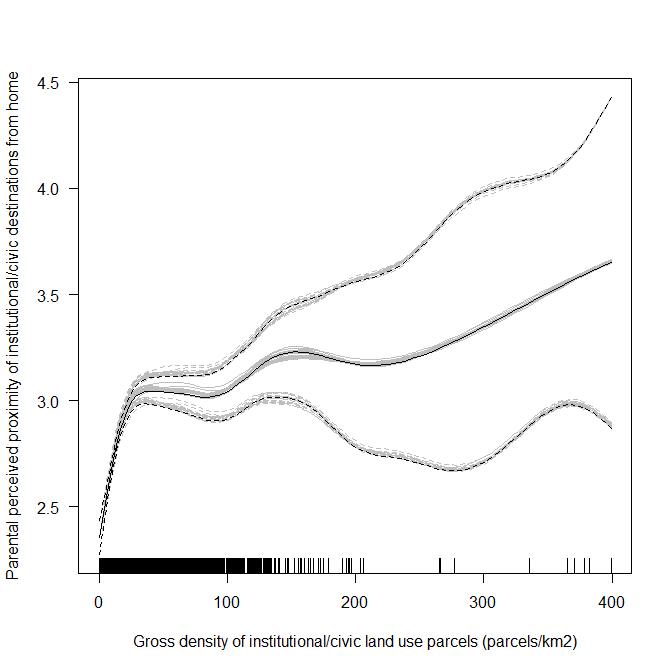


**Figure S5.** Associations of GIS-based gross densities of land use parcels with parental perceived proximities of corresponding destinations. Upper left panel: commercial/office destinations; upper right panel: institutional/civic parcels; lower left panel: food-related parcels; lower right panel: parks.


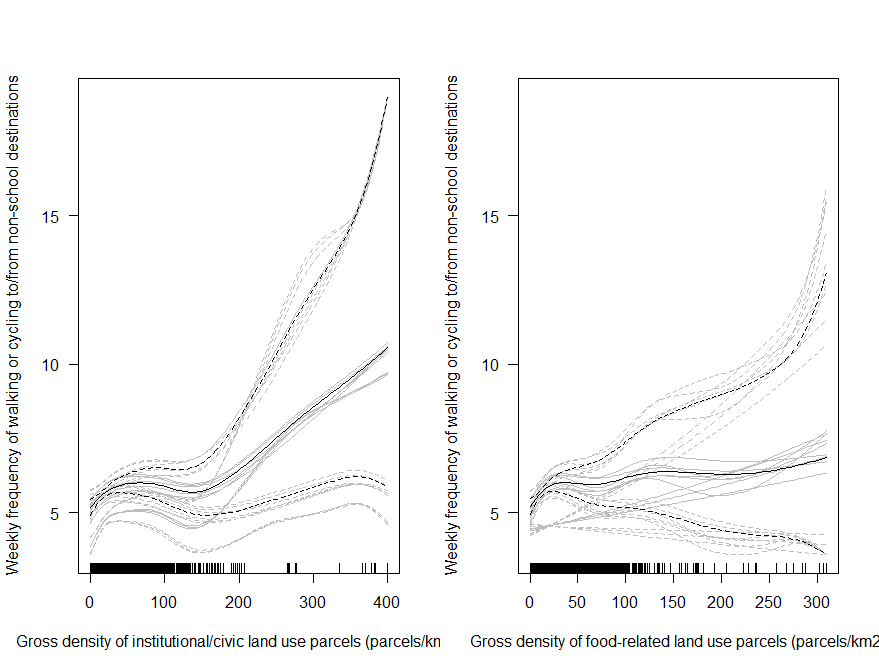


**B**

**A**

**Figure S6.** Associations of GIS-based gross densities of land use parcels with adolescents’ non-school active transport (weekly frequency)
